# A comparison of marker gene selection methods for single-cell RNA sequencing data

**DOI:** 10.1186/s13059-024-03183-0

**Published:** 2024-02-26

**Authors:** Jeffrey M. Pullin, Davis J. McCarthy

**Affiliations:** 1 Bioinformatics and Cellular Genomics, St Vincent’s Institute of Medical Research, 9 Princes St, Fitzroy, 3065 VIC Australia; 2 School of Mathematics and Statistics, University of Melbourne, Parkville, 3010 VIC Australia; 3 Melbourne Integrative Genomics, University of Melbourne, Parkville, 3010 VIC Australia

**Keywords:** Single-cell, Bioinformatics, Benchmarking

## Abstract

**Background:**

The development of single-cell RNA sequencing (scRNA-seq) has enabled scientists to catalog and probe the transcriptional heterogeneity of individual cells in unprecedented detail. A common step in the analysis of scRNA-seq data is the selection of so-called marker genes, most commonly to enable annotation of the biological cell types present in the sample. In this paper, we benchmark 59 computational methods for selecting marker genes in scRNA-seq data.

**Results:**

We compare the performance of the methods using 14 real scRNA-seq datasets and over 170 additional simulated datasets. Methods are compared on their ability to recover simulated and expert-annotated marker genes, the predictive performance and characteristics of the gene sets they select, their memory usage and speed, and their implementation quality. In addition, various case studies are used to scrutinize the most commonly used methods, highlighting issues and inconsistencies.

**Conclusions:**

Overall, we present a comprehensive evaluation of methods for selecting marker genes in scRNA-seq data. Our results highlight the efficacy of simple methods, especially the Wilcoxon rank-sum test, Student’s *t*-test, and logistic regression.

**Supplementary Information:**

The online version contains supplementary material available at 10.1186/s13059-024-03183-0.

## Background

Single-cell RNA sequencing (scRNA-seq) has enabled the high-throughput measurement of gene expression in single cells, enabling the interrogation of cell-type-specific changes in gene expression and regulation. Recently, decreases in cost and advances in protocol efficacy have led to a rapid increase in the number of scRNA-seq datasets used in biological research [1]. Over the same period, there has been a corresponding increase in the number of methods available to analyze scRNA-seq data. As of July 2023, there were over 1500 tools available to perform various steps of an scRNA-seq data analysis [2, 3].

**Fig. 1:**
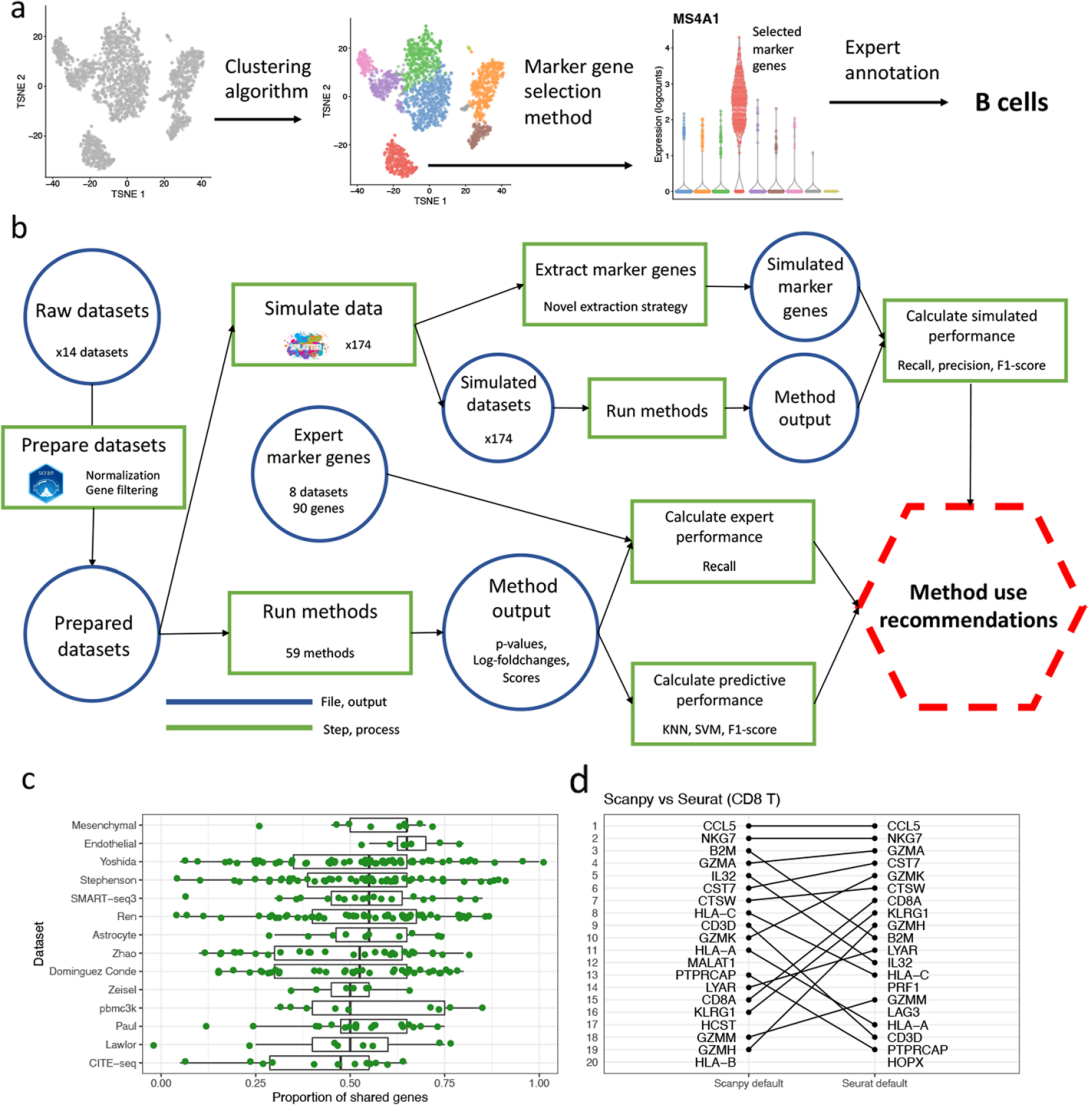
Overview of marker genes usage and benchmarking. **a** A visual overview of the use of marker genes to annotate clusters. First, a clustering algorithm is performed to separate cells into putative clusters. Then, for each cluster, a marker gene selection method is used extract a small number of marker genes. This gene list is inspected and the expression of the genes visualized to give an expert-annotation of cell type for each cluster. **b** A visual overview of the benchmarking performed in this paper. First, the real datasets are processed and the marker gene selection methods are run on the processed datasets. The output of the methods is extracted and used to calculate the methods’ predictive performance and ability to recover expert-annotated marker genes. The processed datasets are also used to simulate additional datasets, on which the methods are run and their ability to recover true simulated marker genes calculated. **c** The proportion of shared genes in the top 20 genes selected by the default methods implemented by Scanpy and Seurat for each cluster across 10 real datasets (127 clusters in total). **d** A visual comparison of the rankings of the top 20 selected genes by the default Scanpy and Seurat methods in the CD8 T cell cluster in the pbmc3k dataset

A ubiquitous step in the analysis of scRNA-seq data is the selection, by a computational method, of so-called marker genes. These marker genes are a small (typically $\le 20$) subset of genes that have expression profiles able to distinguish the sub-populations of cells present in the data. Most commonly, marker genes are selected with respect to a specific computational clustering of the data and are used to annotate, and understand, the biological cell type of the defined clusters (Fig. 1a). Annotation of the cell-type of clusters is critical both to guide the clustering and to interpret the results of downstream analyses performed with respect to the clustering, such as differential expression testing [4] or single-cell eQTL mapping [5]. In addition to cluster annotation, some authors treat the marker genes as new biological discoveries in their own right [6], as candidates for further perturbation or differential analyses [7], or as evidence for the quality of a particular clustering [8]. As well as their direct use, marker genes are a component of computational approaches that aim to annotate clusters automatically [9–11]. Ultimately, marker genes provide a “human interpretable” summary of the transcriptomic profiles of cell sub-populations.

Today, many marker gene selection methods are available. These methods range from relatively simple differential expression based methods to those based on modern machine learning ideas. The most commonly used methods are implemented as part of the Seurat [12] and Scanpy [13] analysis frameworks, which both implement a variety of method options. More recently, bespoke tools have also been developed, as marker gene selection continues to be an active area of method development [7, 14, 15]. However, unlike other areas of scRNA-seq data analysis such as normalization, differential expression analysis or trajectory inference [16–18], no impartial and comprehensive comparison of the different marker gene selection methods is available. The lack of such a comparison leaves analysts unaware of the relative performance of different methods. Furthermore, because the most commonly used methods are implemented in larger analysis frameworks, no publications exist that describe in detail or justify their marker gene selection methods. Therefore, their widely used methods are not supported by publicly scrutinized evidence of their effectiveness.

The concept of the marker gene is closely related to that of the differentially expressed (DE) gene, but the two concepts are not synonymous. Strictly, marker gene selection is a subset of the identification of DE genes, but effective and useful marker genes have specific characteristics that are not shared by all DE genes. Broadly, we define a marker gene as a gene that can be used to distinguish between sub-populations of cells. Good marker genes typically exhibit a large difference in expression between cell types and, canonically, are strongly up-regulated in a cell type of interest, exhibiting high expression in that cell type and no or low expression in other cell types. Thus, a marker gene is a narrower, more specific concept than that of a DE gene, which is context dependent. In the analysis of scRNA-seq data, the term DE gene does not have a unique meaning as it refers generally to a gene that shows a statistically significant difference in expression in a specific comparison. DE genes can therefore refer to genes found in a comparison between cells in different clusters in the same sample [17, 19] or in the same cluster between different samples [4, 20, 21], or to a comparison between arbitrary groups of cells [22]. When DE methods are used to identify marker genes a decision has to be made about how to map the idea (and desirable characteristics) of marker genes to a concrete between-groups comparison. Indeed, different methods use different concrete comparisons: Seurat and Scanpy use a “one-vs-rest” cluster comparison strategy, while scran employs a “pairwise” approach (see the “Methods” section for details). Significantly, both of these strategies are different to those compared in previous benchmarks of single-sample DE methods [17, 23] that focused on comparing two specific cell sub-populations. For example, the “one-vs-rest” strategy creates a situation with highly imbalanced sample sizes and increased biological heterogeneity in the pooled “other” group, both of which we would expect to pose significant challenges to DE methods. As such, marker gene selection used for distinguishing between sub-populations of cells is conceptually and practically a distinctly different task from the more general challenge of identification of all genes with statistically significant differences in expression in a given comparison context, with no consideration for whether or not detected DE genes are useful for distinguishing a given group of cells from others.

The realization that marker gene selection and DE gene detection are distinctly different tasks motivated the current study. We identified that the conclusions from previous, often excellent, benchmarking studies of methods to detect DE genes from scRNA-seq data [17, 23] are not directly portable to the task of marker gene selection. Thus, dedicated benchmarking of marker gene selection methods is necessary given the centrality of marker genes in scRNA-seq analyses and the fact previous DE gene identification benchmarking efforts do not provide actionable advice for marker gene selection. Of course, many methods for marker gene selection are also more general DE gene detection methods, but while many methods can be used for both DE gene detection and marker gene selection we observe that their performance and suitability can differ greatly on the two tasks. Here, we designed and conducted a rigorous benchmarking of methods for the specific and distinct task of the selection of marker genes from scRNA-seq data, taking inspiration and some important lessons from previous DE gene detection benchmarking studies [17, 23].

In this paper, we compared 59 methods for selecting marker genes in scRNA-seq data. The methods were benchmarked using 14 scRNA-seq datasets, encompassing a range of protocols and biological samples, as well as over 170 additional simulated datasets. We benchmark a variety of methods (applying both traditional statistical and modern machine learning approaches) that have been designed specifically for the task of marker gene selection and are not applicable to more general DE gene detection. This study has a substantially different simulation setup compared to DE gene benchmarking studies, one that we designed specifically for the distinct context and challenges of marker gene selection. More than half of the datasets used in this study are bolstered by expert identification of marker genes to which we can compare the marker genes selected by computational methods. This work uses new and specific analyses for assessing marker gene selection methods, compared with prior work benchmarking general DE gene detection methods. Specifically, methods were compared on their ability to recover simulated and expert-annotated marker genes, the predictive performance and characteristics of the gene sets they select, their memory usage and speed, and their implementation quality (Fig. 1b). To scrutinize the methodologies of the most commonly used methods, we created case studies based on specific observations made during benchmarking. These case studies highlight several notable issues with, and inconsistencies between, the methods implemented in the Scanpy and Seurat packages. Importantly, we make different recommendations about preferred methods for marker gene selection compared with recommendations for the more general DE gene detection task from prior DE gene detection benchmarking work, providing immediate, actionable information for researchers relying on marker gene selection in their scRNA-seq data analyses.

Our comparisons and case studies demonstrated that while most methods perform well there is still variability in the quality of the marker genes selected. Significantly, more recent methods were not able to comprehensively outperform older methods. The case studies emphasized that there are large but unappreciated methodological differences even between similar methods, which can have large effects on their output in some scenarios. Overall, our results highlight the efficacy of simple methods, such as the Wilcoxon rank-sum test, for selecting marker genes.

## Results

### Scope of benchmarking

In this paper, we use the phrase “marker gene” as shorthand for “cell-sub-population-specific marker gene”; that is, a gene whose expression distinguishes a particular cell sub-population in a given dataset. As described above, marker genes can be used for a range of purposes, but in this paper, we focus on their use in the annotation of the biological cell-type of clusters. This use of marker genes is by far their most common application in scRNA-seq data analysis. This focus, however, precludes an assessment of other types of methods that also select so-called marker genes. We do not consider methods that aim to select a small set of genes maximally informative for a given full clustering, rather than for each identified cluster [23–25]. We also do not consider the ability of selected marker genes to be used in designing spatial transcriptomics analyses [7, 25]. (See the “Methods” section for more details on these out-of-scope methods.) Finally, we only directly consider the quality of the marker genes in their use for annotation, rather than for other purposes, such as to gauge the quality of a clustering [8] or to provide markers that can be used in wet-lab applications [6]. We believe, however, that methods that select high quality marker genes for annotation will likely also perform well at these other tasks.

### Method characteristics

**Fig. 2:**
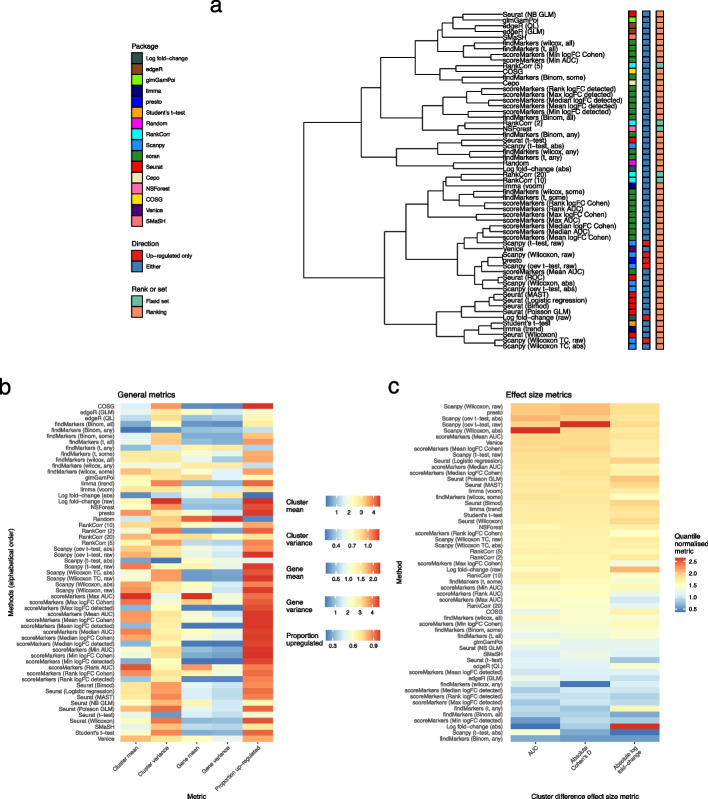
Method concordance and output characteristics. **a** A dendrogram representation of the hierarchical clustering of methods based on the proportion of shared genes in the at most top 20 genes they select. Methods are labeled by the package which implements them, whether they select only up-regulated marker genes or both up- and down-regulated marker genes, and whether they output a set of marker genes or a ranking of genes by strength of marker gene status. **b** A variety of features summarizing (averaging over datasets) the characteristics of the at most top 5 marker genes methods select. Methods are sorted by alphabetical order. **c** Three features (Area under the curve, log fold-change and Cohen’s *d*) summarizing (averaging over datasets) the one-vs-rest effect size of the at most top 5 marker genes that methods select. The three features are quantile normalized and methods are ranked by the median score across datasets

The methods benchmarked in this paper (summarized in Table 1) differ in a number of important ways. First, they differ in the methodology they use to quantify the extent to which each gene can ‘distinguish’ cell types and is therefore a strong marker gene. Most methods use some form of differential expression (DE) testing (Seurat, Scanpy, scran findMarkers(), presto, edgeR, limma). Conversely, other methods use ideas of feature selection (RankCorr), predictive performance (NSForest, SMaSH) or alternative statistics (Cepo, scran scoreMarkers(), Venice) to select marker genes. Within methods based on DE testing, most (Scanpy, Seurat) use one-vs-rest testing while scran uses an alternative pairwise strategy (see the “Methods” section for details). Among DE-based methods a variety of multiple-hypothesis correction methods are used (see the “Methods” section for details). Most DE-based methods compare the overall expression difference between clusters for each cell. This comparison takes into account both the size of any expression difference and the number of cells in which an expression difference exists. The scran-binomial method, however, discretizes expression values as either expressed or not expressed, and therefore only takes the number of cells expressing a gene into account. In addition to differences in methodology there are substantive differences in the outputs of the methods: RankCorr and NSForest return only a specific set of genes determined to be marker genes, while other methods return a ranking of all genes for strength of marker-gene status, generally by *p*-value. While these methods do provide a strategy for selecting a set of marker genes, say taking those with a *p*-value below a given threshold, in practice the strategy is not feasible due to the small size of the returned *p*-values even after multiple testing correction (Additional file 1: Fig. S3, see below), which makes selecting a principled threshold challenging. To account for the difficulty of selecting a fixed set of markers we follow convention and select only a fixed-size set of the top *n* marker genes (for $n = 5, 10, 20$ say) as ranked by the method. In addition, another difference between methods is the direction of regulation of genes the method is designed to select. In this context, a gene is up-regulated (down-regulated) if it is more (less) highly expressed in the cluster of interest relative to other clusters. Up-regulated marker genes are likely to be more interpretable as the presence of gene expression is easier to reason about, and historical use of marker genes has favored selection of up-regulated markers for tasks such as cell sorting. Some methods, including Scanpy by default, will only select up-regulated marker genes, while other methods can select genes that are either up- or down-regulated (Fig. 2b). The marker gene methods also differ greatly in the log fold-changes between the cluster of interest and all other clusters. In particular, Scanpy’s methods select large positive log fold-changes, while most methods select marker genes with log fold-changes around 1. Marker genes with large log fold-change are easier to visualize and interpret. Finally, a notable feature of the space of methods is duplication in methodology amongst the most commonly used Seurat, Scanpy, and scran methods. We explore the relationship between Scanpy and Seurat methods in particular in case studies in this paper.

**Table 1:** Marker gene selection methods benchmarked in this paper

Package	Version	Language	Parameters	Description	Citation
Seurat	4.0.5	R	test.use = t	Welch’s *t*-test	[12]
-	-	-	test.use = “wilcox”	Wilcoxon rank-sum test (with tie-correction)	-
-	-	-	test.use = “LR”	Genewise Logistic regression	-
-	-	-	test.use = “negbinom”	Two group Negative Binomial GLM	-
-	-	-	test.use = “poisson”	Two group Poisson GLM	-
-	-	-	test.use = “roc”	ROC assessment of gene expression as classifier	-
-	-	-	test.use = “bimod”	Bimodal likelihood ratio test	-, [27]
-	-	-	test.use = “MAST”	MAST	-, [19]
COSG	0.9.0	R	None	Cosine score	[15]
Scanpy	1.8.1	Python	test_use = “t-test” rankby_abs = True	Welch *t*-test ranking by the absolute value of the score	[13]
-	-	-	test_use = “t-test_over_estimvar” rankby_abs = True	Welch *t*-test with overestimated variance ranking by the absolute value of the score	-
-	-	-	test_use = “t-test” rankby_abs = False	Welch *t*-test ranking by the raw score	-
-	-	-	test_use = “t-test_over_estimvar” rankby_abs = False	Welch *t*-test with overestimated variance ranking by the raw score	-
-	-	-	test_use = “wilcoxon” rankby_abs = True tie_correct = True	Wilcoxon rank-sum test ranking by absolute value of the score with tie-correction	-
-	-	-	test_use = “wilcoxon” rankby_abs = False tie_correct = True	Wilcoxon rank-sum test ranking by the raw score with tie-correction	-
-	-	-	test_use = “wilcoxon” rankby_abs = True tie_correct = False	Wilcoxon rank-sum test ranking by absolute value of the score without tie-correction	-
-	-	-	test_use = “wilcoxon” rankby_abs = False tie_correct = False	Wilcoxon rank-sum test ranking by the raw score without tie-correction	-
scran	1.22.1	R	findMarkers() test.type = “t”, pval.type = “any”	Pairwise *t*-test up-ranking genes with “any” small *p*-values	[28]
-	-	-	findMarkers() test.type = “t”, pval.type = “all”	Pairwise* t*-test up-ranking genes with “all” small *p*-values	-
-	-	-	findMarkers() test.type = “t”, pval.type = “some”	Pairwise *t*-test up-ranking genes with “some” small *p*-values	-
-	-	-	findMarkers() test.type = “wilcox”, pval.type = “any”	Pairwise Wilcoxon rank-sum test up-ranking genes with “any” small *p*-values	-
-	-	-	findMarkers() test.type = “wilcox”, pval.type = “all”	Pairwise Wilcoxon rank-sum test up-ranking genes with “all” small *p*-values	-
-	-	-	findMarkers() test.type = “wilcox”, pval.type = “some”	Pairwise Wilcoxon rank-sum test up-ranking genes with “some” small *p*-values	-
-	-	-	findMarkers() test.type = “binom”, pval.type = “any”	Pairwise Binomial test up-ranking genes with “any” small *p*-values	-
-	-	-	findMarkers() test.type = “binom”, pval.type = “all”	Pairwise Binomial test up-ranking genes with “all” small *p*-values	-
-	-	-	findMarkers() test.type = “binom”, pval.type = “some”	Pairwise Binomial test up-ranking genes with “some” small *p*-values	-
scran	1.22.1	R	scoreMarkers(), mean.logFC.cohen	Pairwise Cohen’s *d*, mean across genes	NA
-	-	-	scoreMarkers(), min.logFC.cohen	Pairwise Cohen’s *d*, minimum across genes	-
-	-	-	scoreMarkers(), median.logFC.cohen	Pairwise Cohen’s *d*, median across genes	-
-	-	-	scoreMarkers(), max.logFC.cohen	Pairwise Cohen’s *d*, maximum across genes	
-	-	-	scoreMarkers(), rank.logFC.cohen	Pairwise Cohen’s *d*, min rank across genes	-
-	-	-	scoreMarkers(), mean.AUC	Pairwise AUC, mean across genes	-
-	-	-	scoreMarkers(), median.AUC	Pairwise AUC, median across genes	-
-	-	-	scoreMarkers(), min.AUC	Pairwise AUC, minimum across genes	-
-	-	-	scoreMarkers(), max.AUC	Pairwise AUC, maximum across genes	-
-	-	-	scoreMarkers(), rank.AUC	Pairwise AUC, min rank across genes	-
-	-	-	scoreMarkers(), mean.logFC.detected	Pairwise lfc in detection proportion, mean across genes	-
-	-	-	scoreMarkers(), min.logFC.detected	Pairwise lfc in detection proportion, minimum across genes	-
-	-	-	scoreMarkers(), median.logFC.detected	Pairwise lfc in detection proportion, median across genes	-
-	-	-	scoreMarkers(), max.logFC.detected	Pairwise lfc in detection proportion, maximum across genes	-
-	-	-	scoreMarkers(), rank.logFC.detected	Pairwise lfc in detection proportion, min rank across genes	-
presto	1.0.0	R	None	Optimized Wilcoxon rank-sum test	NA
edger	3.36.0	R	GLM	Negative Binomial GLM with empirical Bayes (EB) shrinkage	[29, 30]
edger	3.36.0	R	QL	- With quasi-likelihood fitting	[29, 30]
RankCorr	NA	Python	lambda = 2	Sparse seperating hyperplane selection	[26]
RankCorr	NA	Python	lambda = 5	-	[26]
RankCorr	NA	Python	lambda = 10	-	[26]
glmGamPoi	1.6.0	R	None	Negative Binomial QL GLM with EB shrinkage	[31]
limma	3.50.0	R	Standard	Linear model with EB shrinkage	[32, 33]
limma	3.50.0	R	Voom	- With weighting	[32, 33]
limma	3.50.0	R	Trend	-	[32, 33]
Cepo	1.0.0	R	None	Stability statistic	[34]
NSForest	3.0	Python	None	Random forest classifier	[14]
Venice	0.0.11	R	None	Classification scoring	[35]
SMaSH	0.1.2	Python	None	Deep neural net classifier	[7]

### Benchmarking strategy

To compare the performance of the different methods, we use a variety of metrics intended to provide a holistic assessment of a method’s performance. The diversity of techniques used by the methods to select marker genes means it is important that the metrics used to benchmark methods are not biased towards a particular set of techniques. For example, a simulation-based comparison could employ a similar generative probabilistic model to those used to perform statistical testing, potentially leading to overoptimistic conclusions. For this reason, we compare methods based on both their ability to recover simulated marker genes and the predictive performance of the gene sets they select. In addition, whether a gene is a marker gene depends on whether it is “known” to be a marker gene [26]. Methods may be most useful if they find known marker genes, even if genes which better distinguish cell subpopulations are present in a dataset. To benchmark the methods’ ability to select “known” marker genes we compare their ability to select expert-annotated marker genes in 8 datasets. In addition, we compare the methods based on their speed and memory efficiency and the quality of their software implementations. Overall, we benchmarked the performance, computational efficiency, and output characteristics of 59 methods (Table 1) across 14 real datasets (Table 2) and over 170 additional simulated datasets. The real datasets were chosen to represent a range of protocols—including 10X Chromium, Smart-seq3, CITE-seq, and MARS-seq—and a range of sizes, from approximately 3000 to 60,000 cells.

**Table 2:** scRNA-seq datasets used in this paper

Dataset	*N*. cells	*N*. cell populations	Description	Technology	Citation
Zeisel	3005	7	Mouse brain	10X Chromium	[72]
pbmc3k	2638	9	PBMC	10X Chromium	[37]
Lawlor	604	8	Human pancreas	SMARTer v1	[38]
Endothelial	30,641	7	Human lung endothelium	10X Chromium	[39]
Astrocyte	3013	10	Mouse brain astrocytes	Single-nucleus RNA-seq	[8]
Paul15	2730	19	Mouse hematopoietic stem cells	MARS-seq	[40]
Zhao	60,672	29	Human liver resident immune cells	10X Chromium	[41]
SS3 PBMC	3128	17	PBMC	Smart-seq3	[42]
CITE-seq	8617	12	CBMC	CITE-seq	[12]
Mesenchymal	11,408	9	Human lung mesenchymal	10X Chromium	[39]
Stephenson	25,000	15	Human blood	10X Chromium	[43, 44]
Ren	25,000	16	Human blood	10X Chromium	[43, 45]
Yoshida	46,431	19	Human blood	10X Chromium	[43, 46]
Dominguez Conde	25,980	11	Human blood	10X Chromium	[43, 47]

### Method concordance and selected marker gene characteristics

First, we compared the extent to which the different methods produced concordant gene sets. To motivate this analysis and the benchmarking more broadly, we compared the concordance of the default methods implemented by Scanpy and Seurat, which are the two most commonly used methods. For the top 20 genes selected by the methods, we calculated the proportion of genes shared between the methods and visualized the difference in their rankings. Despite their similar methodology the methods showed only poor to moderate concordance. Across datasets many clusters showed less than 50% overlap between the two methods (Fig. 1c). Even in clusters with a high proportion of shared genes, such as the CD8 T cluster in the pbmc3k dataset, the methods’ rankings were quite different (Fig. 1d).

To examine the concordance of all the methods more generally, we performed a hierarchical clustering based on the proportion of genes shared between methods averaged across clusters and datasets (Fig. 2a; see the “Methods” section for details). This analysis recapitulated many known features of the space of methods: the dendrogram groups together similar methodologies implemented in different packages, such as the Wilcoxon-based methods in Seurat of Scanpy or methods based on negative binomial regression, and methods that only select up-regulated genes (Fig. 2). In addition, the clustering also revealed that some Seurat methods with different methodologies give similar rankings. Calculating the dendrogram on the results for specific datasets showed qualitatively similar conclusions (Additional file 1: Fig. S1). Also, the proportion of genes shared between methods did not show a relationship to the number of cells in a given cluster (Additional file 1: Fig. S2).

Next, we examined the characteristics of the marker genes produced by the analysis. First, we calculated a series of metrics that summarize general properties of the selected marker genes, including the overall mean expression of the gene selected and the proportion of selected marker genes that were up-regulated (Fig. 2b). In this context, up-regulated means that the gene is more highly expressed in the cluster of interest than in other clusters. These metrics were calculated for the at most top 5 genes selected by each method in the all real datasets and the median over clusters and datasets was visualized. These metrics highlight a number of patterns amongst the marker genes that the different methods select. Most methods select a majority of up-regulated marker genes. Indeed, some methods including including presto, scran’s findMarkers() Wilcoxon and scoreMarkers() methods, and the Scanpy’s raw methods select almost all up-regulated genes, while SMaSH and methods based on negative binomial GLMs, Welch’s *t*-test and binomial tests select more down-regulated marker genes. In addition, the genes that methods select differ in terms of the mean and variance of their cluster-specific expression. For example, scran’s scoreMarkers() methods that binarised expression (“detected” expression) select genes with systematically lower expression. On the other hand, Seurat’s methods show systematically higher variance in the cluster of interest compared to scran’s scoreMarkers() methods.

Second, we calculated measures of effect sizes for the selected marker genes. Ideally, methods would select genes which have a large difference in gene expression in the cluster of interest, compared to expression in all other clusters. To measure the effect size, we used AUC (i.e., area under the receiver operator characteristic curve using the expression data as a classifier for binary cluster status) and absolute values of Cohen’s *d* statistic and the log fold-change, all in a one-vs-rest manner. These metrics were calculated for the at most top 5 genes selected by all methods. For visualization the median was taken over clusters and datasets, and quantile normalization was applied to make the metrics’ distributions comparable. Methods are ranked by their median values over the three metrics (Fig. 2c). Of the three metrics, Cohen’s *d* and AUC are highly concordant. Presto and Scanpy’s methods select genes with the largest overall effect sizes, while scran’s findMarkers() “any” methods, Scanpy’s *t*-test (abs), and ranking by the absolute value of the log-fold change have the lowest effect median effect sizes. The genes selected by ranking by the absolute value of the log fold-change generally have extremely low expression in the cluster of interest and low expression in all other clusters causing large log fold-changes but small differences in other effect sizes.

Finally, for the DE-based methods we extracted and visualized *p*-values calculated by the methods. As noted above, the *p*-values were extremely small even after conventional multiple testing correction (Additional file 1: Fig. S3a, b). The small magnitude of the *p*-values is caused by the large sample sizes in the datasets (where each cell is treated as a replicate) and double dipping inherent in performing clustering and statistical testing on the same data. Surprisingly, many methods showed *p*-values of exactly 0, especially in the larger Zhao and Endothelial datasets (Additional file 1: Fig. S3c). This feature occurred for methods implemented in both R and Python, and has a large impact on Seurat’s methods as described in the case studies section.

### Performance on simulated marker genes

To critically assess the performance of the methods, we first compared their ability to recover true simulated marker genes. We use the splat simulation model implemented in the splatter R package to simulate scRNA-seq datasets [48]. The splat model is the most widely used simulation model in scRNA-seq benchmarking studies [49] and was recently shown to be in the top tier of simulation methods [50]. To simulate marker genes specifically, we designed and implemented a novel marker gene score to select and rank marker genes using the splat model’s cluster-specific differential expression parameters (Methods). A limitation of the splat model is that it does not allow these cluster-specific differential expression parameters to be estimated from data. Indeed, no current simulation scRNA-seq method incorporates this facility [49]. To overcome this limitation in practice we performed analyses comparing simulated and true marker genes to identify values of the parameters that were able to recapitulate true expert-annotated marker genes (Additional file 1: Figs. S8-9: compare with e.g., S5-6). Our simulations also include sensitivity analyses using a range of plausible values for these parameters.

**Fig. 3:**
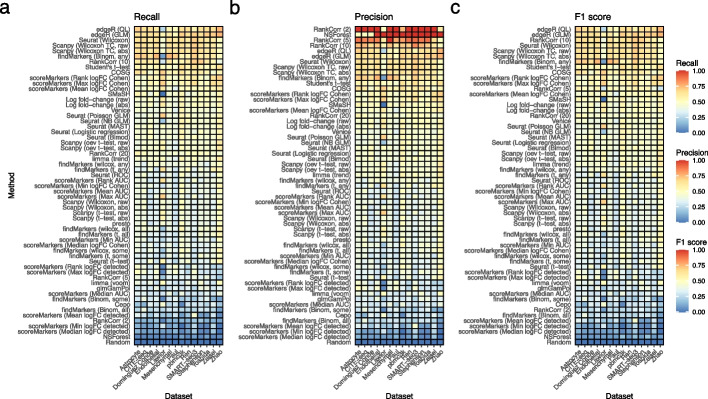
Comparison of methods using simulated datasets. **a** Calculated recall for all methods on simulation scenarios based on all real datasets. The marker genes selected based on the simulation model parameters are used as the ground truth. Methods are ranked top to bottom in the heatmap by median recall across scenarios. **b** As in (**a**) but now with precision. **c** As in (**b**) but calculating F1 score. These results average over simulated clusters and simulation replicates and are conducted with 20 genes selected and a location parameter for the DE factor in the splat simulation model of 3

Ten simulation scenarios were considered, each with parameters estimated from one of the real datasets (Table 2). All simulations used to compare performance had 2000 genes, 2000 cells, and 5 clusters. In this analysis we only simulate up-regulated marker genes and the outputs of the methods are also filtered to include only up-regulated marker genes (see the “Methods” section for details). Each simulation scenario was repeated 3 times to gauge variability. For each method in each simulated cluster we select the top 20 simulated marker genes and the (at most) top 20 marker genes selected by each method. If a method returns less than 20 marker genes per cluster, we use only the genes the method returns. With the top sets of genes we computed the precision, recall, and F1 score of each method in each cluster and replicate (Additional file 1: Fig. S12). This analysis showed little replicate- and cluster-specific variability so we summarized the performance of each method in each scenario by taking the median across clusters and replicates (Fig. 3).

Overall, we found concordant results across simulation scenarios. Assessed by recall (the proportion of all true marker genes that are selected as marker genes by the method), the best performing methods were edgeR, scran’s Binomial-any method, Wilcoxon rank-sum based methods (Seurat and Scanpy), Student’s t-test, and RankCorr with the lambda parameter set to 10. The worst methods were NSForest, Cepo and RankCorr ($\lambda$ = 2) (Fig. 3a). Despite the strong performance of scran’s Binomial-any method the rest of scran’s methods showed relatively weak performance. Assessed by precision (the proportion of marker genes a method selects that are true marker genes), the best performing methods were RankCorr and NSForest as well as scran Binomial-any (Fig. 3b). The discrepancy between precision and recall for RankCorr and NSForest occurs because RankCorr and NSForest only select a very small number of genes. To provide an overall ranking of the methods we calculated the F1 score (a combination of recall and precision) for each method and dataset and then ranked the methods by their median performance over datasets (Fig. 3c). In this ranking, the best performing methods were RankCorr ($\lambda$ = 10), the Wilcoxon rank-sum based methods, and edgeR’s methods. NSForest, Cepo, and scran’s other Binomial methods showed the worst performance. While the results were generally concordant between scenarios the Lawlor dataset-based scenarios showed some differences: here the log fold-change methods and Seurat’s Poisson GLM were more effective, while scran Binomial-any was less effective. This difference is likely caused by the higher mean counts of the Lawlor dataset relative to other datasets (Additional file 1: Fig. S4).

Further analyses assessed the sensitivity of these results to different analysis parameters. Using different values ($n = 5, 10, 20, 40$) for the number of genes had little effect on the overall ranking, though edgeR showed improved performance when 20 and 40 genes were used (Additional file 1: Fig. S10). Using different values of the cluster-specific differential expression parameters also had little effect, except that the COSG method performed better when smaller values were used (Additional file 1: Fig. S11).

### Expert-annotated marker gene recovery

Next, we compared the ability of the methods to recover expert-annotated sets of marker genes. This analysis is intended to assess the ability of methods to select genes which are *known* marker genes. We used expert-annotated marker gene sets for four datasets: the Lawlor, Smart-seq3, pbmc3k, and Zeisel datasets, derived mainly from the papers and tutorials describing these datasets (see the “Methods” section for details). In addition, for the four harmonized blood datasets (Ren, Stephenson, Yoshida and Dominguez Conde) we used marker genes drawn from a recent immune-cell atlas, created by the same group that harmonized those datasets [43, 47]. Initially, we hoped to perform a comparison based on genes available in online databases such as CellMarker [51] and PangloDB [52], however we found that there were substantial differences between databases, and that marker-gene coverage for specific cell types was typically sparse. In addition, it was difficult to harmonize cell-type labels between databases.

**Fig. 4:**
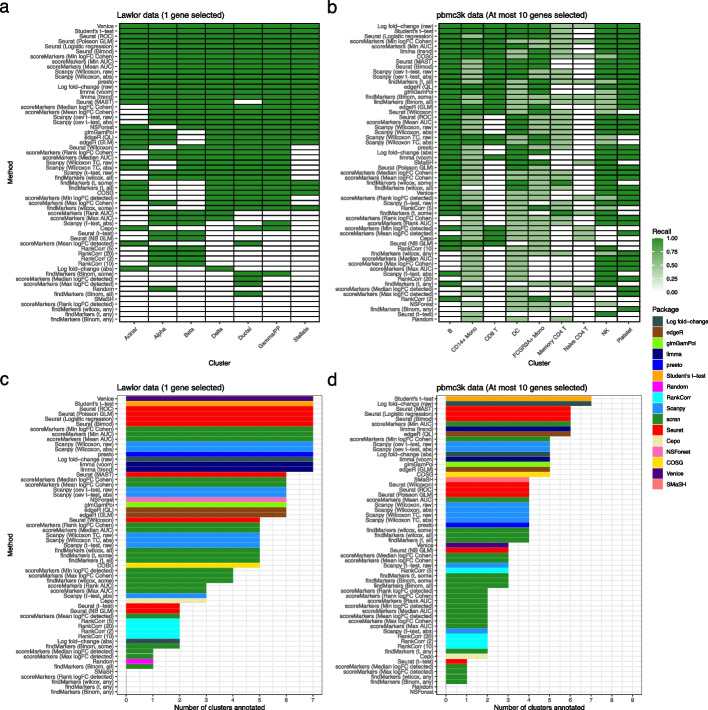
Comparison of methods based on expert-annotated marker genes. **a** Recall of methods when selecting marker genes in the Lawlor dataset using a set of expert-annotated marker genes as the ground truth. The marker genes used to annotate the clustering in the original publication describing the Lawlor dataset were used as the set of expert-annotated marker genes. The top gene was selected from the output of each method. **b** As in (**a**) but for the pbmc3k dataset, using the (at most) top 10 marker genes from each method and taking the set of expert-annotated marker genes to be those used in the Seurat package’s “Guided Clustering Tutorial.” **c** The number of clusters that are successfully annotated using the selected marker genes in the Lawlor datasets (other details as in **a**). A specific cluster is defined as successfully annotated if the selected marker genes include all the expert-annotated marker genes for that cluster. **d** The same success of annotation analysis as in (**c**) but for the pbmc3k dataset, with the details of the expert-annotated marker genes and number of selected marker genes as in (**b**)

Concretely, for each method and cluster we computed recall using the expert-annotated marker genes as the ground truth. Furthermore, we calculated the number of clusters that could be successfully annotated using the method’s selected marker genes. For this calculation we considered a cluster to be successfully annotated if all its associated expert-annotated marker genes were selected by the method. In these analyses, the number of selected marker genes used was based on the number of expert-annotated marker genes per cluster. First, for the Lawlor dataset, we used the canonical markers used for classification in the paper describing the dataset [38] and selected the top gene for each method. In this dataset, Venice, Seurat’s ROC, logistic regression, Poisson GLM and Bimod methods, presto, limma, ranking by the raw log fold-change, Scanpy’s Wilcoxon methods, scoreMarkers()’s min-cohen, min-AUC and mean-AUC methods and Student’s *t*-test successfully selected all expert-annotated marker genes while SMaSH and again scran’s findMarkers() methods and Cepo showed poor performance (Fig. 4a, c). Second, for the pbmc3k dataset we used the marker genes given in the Seurat Guided Clustering Tutorial and used the (at most) top 10 selected genes. In this dataset, the best performing methods were the log fold-change (raw), and Student’s *t*-test, while the worst performing methods were the *t*-test-based scran methods, Cepo, and RankCorr (Fig. 4b, d). For example, the Student’s *t*-test selected marker genes could be used to annotate 7 clusters, while those selected by Cepo could only annotate 2 (Fig. 4d). Strikingly, all methods struggled to select the expert-annotated marker genes for the Memory and Naive CD4 T cell clusters. This effect occurs because the expert-annotated marker genes for these clusters, especially *IL7R*, have expression profiles that separate all T cells from other clusters, not the specific T cell subtypes of interest (Additional file 1: Fig. S7).

Similarly, we calculated the performance of the methods on the expert-marker genes for the Smart-seq3 and Zeisel data. In these comparisons the relative performance of the methods was similar to the pbmc3k and Lawlor datasets but the absolute performance of methods was generally worse, although some scran methods did show improved performance in the Smart-seq3 dataset (Additional file 1: Figs. S13-14). Examination of the results suggested that the poor absolute performance was mainly due to marker genes being difficult to select or because the methods selected other strong marker genes. For example, *PECAM1*, the marker of Naive/Memory CD8 T cells in the Smart-seq3 PBMC dataset, is not found because its expression profile means that it is only a good marker relative to other T cells and not globally, which is the implicit frame of reference of the methods (Additional file 1: Fig. S17). On the other hand, in the Zeisel dataset’s oligodendrocyte cluster Seurat’s Wilcoxon method’s top 10 selected marker genes are all strong markers, but the expert-annotated marker gene *HAPLN2* is simply not selected (Additional file 1: Fig. S6). In addition, examining the genes selected by poor performing methods for these datasets reveals reasons for their poor performance. For the interneuron cluster of the Zeisel dataset the top 2 genes selected by Seurat’s *t*-test method are strongly up-regulated in the interneuron cluster but are also up-regulated in most other clusters (Additional file 1: Fig. S6a). In contrast, the top 2 genes selected by scran’s *t*-test-any method for the interneuron cluster of the Zeisel dataset show strong up-regulation only in a different (i.e., not interneuron) cluster, making them poor marker genes of the interneuron cluster (Additional file 1: Fig. S6b).

In the harmonized blood datasets, most methods failed to select all marker genes across clusters (Additional file 1: Figs. S15-16). The best performing methods across the four datasets were Wilcoxon methods, SMaSH, RankCorr with $\lambda = 2$, Student’s *t*-test, and limma. The scoreMarkers() methods in scran performed poorly (Additional file 1: Figs. S15-16).

### Predictive performance

**Fig. 5:**
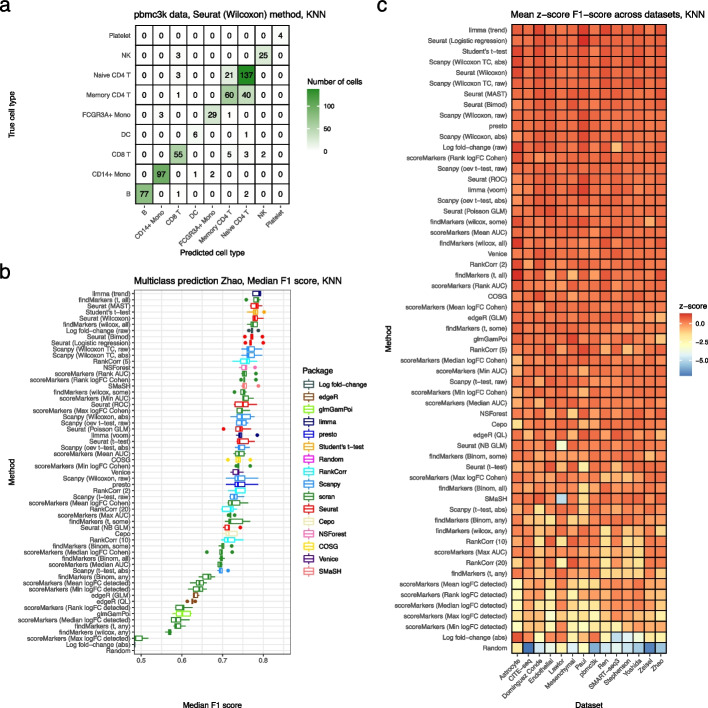
Comparison of methods using predictive performance. **a** A confusion matrix representation of the performance of a KNN (three nearest neighbors) classifier using the set of genes selected by the Seurat Wilcoxon method in the pbmc3k dataset. **b** Median F1 score of the KNN classifier using genes selected by all marker gene selection methods in the Zhao dataset. Each point is the F1 score in one of the 5 folds. **c** The *z*-score of the median F1 score of the KNN classifier (averaging across folds) in each dataset. Methods are ranked top to bottom by their mean *z*-score across datasets

To gain a different perspective on marker gene selection, we compared the predictive accuracy of the gene sets selected by competing methods. The idea of this comparison is that better gene sets should capture more “information” about which cells are in each cluster. We quantify the amount of information by measuring the predictive performance of a classifier for multi-class cluster status trained on only the gene sets selected by the methods. Specifically, we select the top 5 marker genes for each method, dataset, cluster combination. Three different classifiers were tested: a KNN classifier, an SVM classifier, and a more direct alternative: classifying each cell as the cluster with the highest summed expression across that cluster’s marker genes. (In general, the methods gave highly concordant results so we only visualize the results from the KNN classifier in the main text.) The KNN and SVM classifiers were trained using the marker genes pooled across cell types and assessed using 5-fold cross validation, while the summed max classifier was tested using the 5-fold test sets as it had no tunable parameters. While conceptually distinct from other comparison methods applied in this paper, this comparison was able to reproduce features seen in other comparisons, such as the difficulty of distinguishing between the Naive CD4 T and Memory CD4 T cells in the pbmc3k dataset (Fig. 5a). To assess the performance of the methods we calculated a median F1 score across clusters for each fold and dataset (Fig. 5b). The classifiers gave similar F1 scores, though the summed maximum expression classifier had generally lower performance (Fig. 5b, Additional file 1: Figs. S18a, S19a). To summarize each method’s performance across datasets, we computed the *z*-score transformation of median F1 score within datasets, noting the low between-fold variability, and then ranked the methods by their mean *z*-scores (lowest first) across datasets (Fig. 5c, Additional file 1: Figs. S18b, S19b). Using *z*-scores accounts for the wide variability in F1 scores observed across datasets, likely due to differences in the quality of their clusterings. The three classifiers gave similar rankings. Across datasets and classifiers the best performing methods are limma (trend), Seurat logistic regression, Wilcoxon-test methods, and t-test methods other than Seurat’s *t*-test; the worst performing methods included Cepo, scran’s scoreMarkers() methods, Seurat’s t-test method, NSForest, absolute value log fold-change ranking, and scran’s binomial test methods.

### Computational performance and implementation quality

**Fig. 6:**
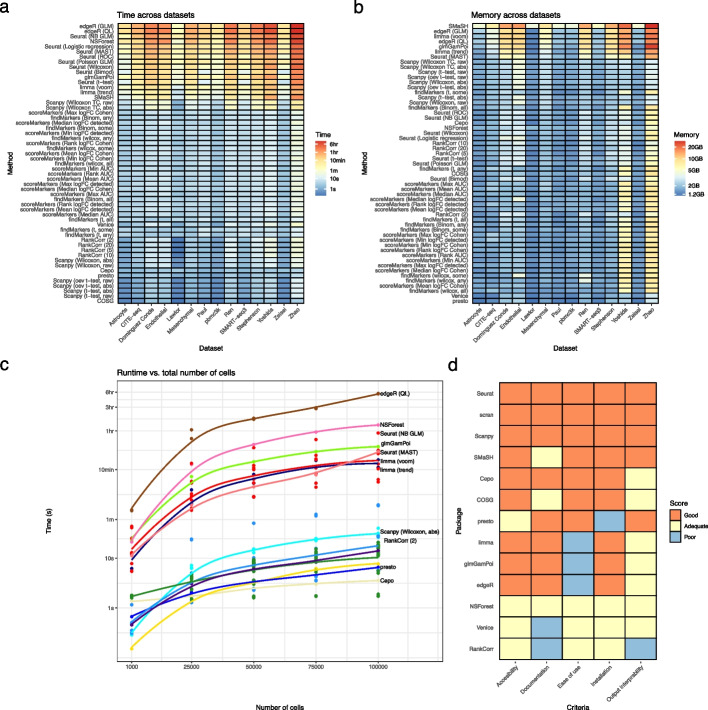
Comparisons of methods' computational performance and implementation quality. **a** Heatmap displaying the time taken for all methods to run across all 10 real datasets. Methods are ranked top to bottom in the heatmap by median time taken over datasets, largest at the top. Note that the color scale of the heatmap is a log scale. **b** Heatmap displaying the memory usage of all methods across all datasets. Methods are ranked top to bottom in the heatmap by median memory usage over datasets, largest at the top. **c** Time taken for all methods on simulated datasets with increasing numbers of total cells. Points are averages over 3 simulation replicates. All simulations had parameters estimated from the pbmc3k dataset and a location parameter for the DE factor of 3. **d** Assessment of the implementation quality of packages which implement methods for selecting marker genes based on 5 criteria

Finally, we compared the computational performance of the methods and the quality of their implementations. First, the methods were compared based on their speed and memory consumption (see the “Methods” section for details of how these were measured). Speed is particularly important for marker gene selection methods given the common need to run them multiple times when iterating through different clusterings of the data. The methods were run on all datasets as well as additional simulated datasets where the total number of cells and number of clusters were varied. Running the methods on all datasets highlighted that the methods had a very wide range of speeds (Fig. 6a). For example, on the large Zhao dataset (60,672 cells) edgeR runs in over 10 h while Scanpy’s default method runs in less than 15 s. Overall, the slowest methods were the edgeR methods, Seurat’s NB GLM and MAST methods, and NSForest, while the fastest were most of Scanpy’s methods, presto, Cepo RankCorr, and COSG. Furthermore, we observed that Seurat’s methods are substantially slower than the scran and Scanpy methods even when implementing the same statistical tests. For example, on the Zhao dataset Seurat’s *t*-test method took over 90 minutes to run, while the equivalent Scanpy method took 14 seconds. These results were strongly concordant across simulations with varying numbers of total cells and number of clusters, which showed little change in the ordering of methods as the number of cells or clusters increased (Fig. 6c, Additional file 1: Fig: S20a). Next, we compared the memory usage of all methods across datasets (Fig. 6b). These measurements showed that memory usage was substantially higher across methods for the larger datasets (Endothelial, Mesenchymal, Zhao) studied. The SMaSH method used the most memory, while edgeR, limma (voom), and glmGamPoi methods were also memory intensive, while scran methods, presto and Venice used the least. Simulations with varying numbers of total cells and clusters particularly highlighted the high memory usage of edgeR, glmGamPoi, and limma when the total number of cells was high (Additional file 1: Figs. S21, S20b)

For a final assessment of usability, we compared the quality of the software implementing each method. Although this analysis is somewhat subjective, how easy a method is to install and run is one of the strongest determinants of its practical utility. For this analysis, we consider methods on the level of packages. To compare the packages, we assessed their performance as either good, adequate or poor for five criteria: accessibility, installation, documentation, ease of use and the interpretability of output (Fig. 6d; see the “Methods” section for details). Overall, the Seurat, Scanpy, and scran packages have excellent implementations. The SMaSH method’s implementation is good, though with limited documentation. Cepo’s implementation is very good except for difficult-to-use output that mangles cluster names. COSG has a good implementation, though with somewhat limited documentation and output interpretability. Next, edgeR, limma, glmGamPoi, and presto all have good implementations with particular issues: the general purpose edgeR, limma and glmGamPoi methods need additional code to be used to select marker genes, while presto is difficult to install and is not available from standard repositories. Finally, NSForest and RankCorr are implemented only in the form of Python scripts in GitHub repositories with little documentation.

### Impact of dataset characteristics

To better understand the performance of different methods, we also assessed the impact of various dataset characteristics on method performance and concordance. First, we visualized the predictive-performance of methods broken down by cell type (Additional file 1: Fig. S22). The effect of cell type was larger than differences between methods in many datasets. Methods performed better on cell types that were phenotypically very distinct from other cells in the dataset, such as the platelet or B cell clusters in the pbmc3k dataset. Next, we visualized the predictive performance of all methods by the number of cells in the cluster (Additional file 1: Fig. S23) and numbers of clusters (Additional file 1: Fig. S24). Generally, methods performed better on smaller clusters, possibly because they represent better defined biological cell types. However, unlike most methods, the SMaSH method performed much worse when the cluster size was less than 100. As the number of clusters in the datasets increase methods generally performed worse, though the effect was small. This trend suggests that methods perform worse when datasets are more diverse. However, when methods were run on simulated datasets with different numbers of clusters, no effect of cluster size was observed (Additional file 1: Fig. S25). Taken together, these results suggest that diversity, at least as measured by the number of clusters, does not have a large impact on method performance. No methods displayed particularly strong performance in the high resolution scenarios where there are many clusters.

Next, to assess the effect of dataset characteristics on method stability we performed simulations where the number of clusters and cells were reduced by down-sampling the pbmc3k dataset (Additional file 1: S26). Method stability is assessed (see the “Methods” section for details) by calculating the proportion of at most top 10 genes selected that are the same as the genes selected when the methods are run on the full dataset. These simulations demonstrate that as both the numbers of cells and clusters were reduced method performance decreased compared to performance on the full dataset. These perturbations show that as both the number of cells and clusters decrease method performance decreases, but the decrease is larger when the number of clusters decreases, highlighting methods’ sensitivity to the cluster structure. Methods that had generally better performance in the benchmarking were more stable when the number of cells was reduced, but scran’s scoreMarkers() methods showed good stability when the number of clusters was reduced, likely due to its use of a pairwise comparison approach.

Finally, we assessed the concordance of methods when run on the same cell type in multiple datasets. To perform this analysis we used four Human Cell Atlas blood datasets harmonized using recent automatic methods [43]. Methods were run, returning the (at most) top 10 genes, on all clusters that appeared in all 4 datasets. To summarize concordance, we calculated the proportion of all selected genes that were selected in all 4 datasets (Additional file 1: Fig. S27). Overall, methods were moderately concordant across datasets, with substantial variability between methods and clusters. The most concordant methods were the Student’s *t*-test method and the scoreMarkers() methods that used Cohen’s *d*. The three T cell subtypes showed substantially less concordance across methods. As methods for harmonization of cell types are still emerging, these results may be sensitive to the details of the harmonization strategy used.

### Case studies

During the overall benchmarking we noticed several undocumented features of the methods implemented in Scanpy and Seurat, which can lead both to inconsistencies between Scanpy and Seurat and substantive issues with their methods’ output.

The major differences between Seurat and Scanpy’s methods are the strategies they use to rank genes after differential expression testing has been performed. Seurat ranks genes based first on their corresponding *p*-value (smallest first) and secondly (i.e., to break ties in the *p*-values) by the raw value of the log fold-change calculated for each gene (largest first). On the other hand, Scanpy ranks genes based on a test statistic or “score” used in the calculation of the differential expression for each gene. By default, genes are ranked by the raw value of the statistic, such as a *z*-score, but they can be optionally ranked by the absolute value of the statistic. For the statistics implemented in Scanpy, the sign of the statistic is the same as the log fold-change calculated for each gene.

These differences cause several issues. First, it creates a situation whereby the two methods select qualitatively different sets of marker genes: as noted above Scanpy by default only selects marker genes that are up-regulated, while Seurat by default can select both up- and down- regulated marker genes (Fig. 2b). While both options are sensible, it is unlikely that many analysts recognize this difference or check that default method behavior matches their expectations for marker gene selection.

**Fig. 7:**
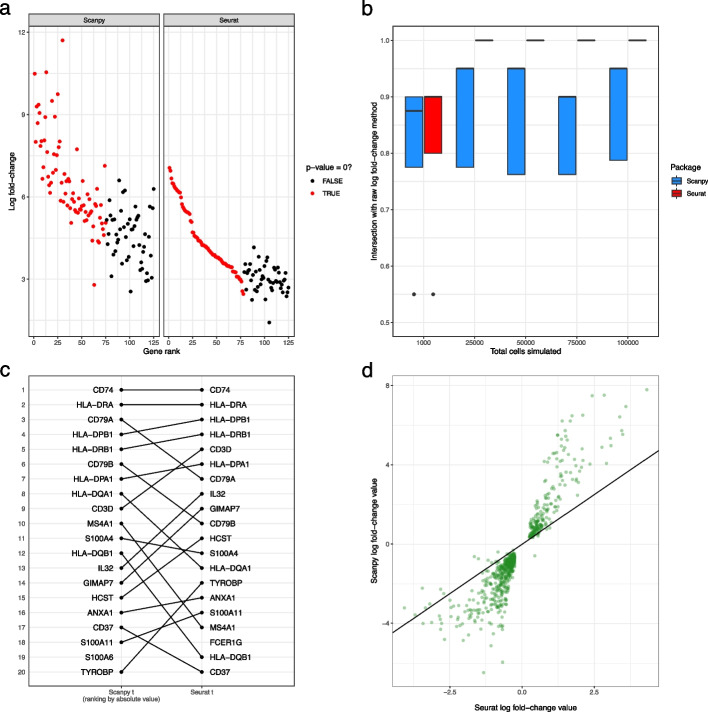
Case studies scrutinizing Scanpy and Seurat. **a** Gene rank vs log fold-change values for the Scanpy Wilcoxon (with tie correction, ranking by the absolute value of the score) and Seurat Wilcoxon methods for the Oligodendrocyte cell type cluster in the Zeisel dataset. The color of the point indicates whether or not the gene has an exactly zero* p*-value. **b** The proportion of top 20 genes shared between methods implemented in Scanpy and Seurat and the method of ranking genes by the raw log fold-change calculation on simulated datasets with increasing number of total cells. These simulations have parameters estimated from the pbmc3k dataset and a location parameter for the DE factor of 3. **c** Visualization of difference in rankings between the Scanpy *t* method (ranking by the absolute value of the score) and Seurat’s *t*-test method on the B cell cluster in the pbmc3k dataset. **d** Scatter plot between the log fold-change values calculated by Seurat and Scanpy on the B cell cluster in the pbmc3k dataset

Next, returning many *p*-values exactly equal to zero, as observed to occur for large datasets (Fig. 2c), leads to an unexpected and undocumented change in the output of Seurat’s methods. The ranking of genes with zero *p*-values, of which there can often be more than the number of marker genes selected, is completely determined by the gene’s log fold-change value. (Fig. 7a). Therefore, for large datasets, the output from Seurat’s methods will be the same as ranking by the (raw) log-fold change, while Scanpy’s methods are different (Fig. 7b).

Finally, Scanpy’s ranking by score means that its default methods output a mathematically incorrect ranking of genes. Scanpy uses both a *z*-score (Wilcoxon rank-sum test) and a *t*-value (Welch’s *t*-test) to rank genes. Due to the monotonicity of the cumulative density function, ranking by the absolute value of the *z*-score is equivalent to ranking by the *p*-value (see the “Methods” section). However, this equivalence does not hold for the *t*-value calculated from Welch’s *t*-test (Methods). This difference can be seen when comparing the output between Seurat and Scanpy Welch *t*-test methods: some genes, including those in the top 5, change their rankings substantially (Fig. 7c).

In addition, there are other differences between Scanpy and Seurat, beyond their ranking strategies. First, by default they use different versions of the Wilcoxon rank-sum test. By default, Scanpy does not implement the conventional correction for ties (although it will with certain options set), while Seurat does. Second, a more serious difference is that the methods calculate different values for the same gene’s log fold-change in the same dataset (Fig. 7d). This discrepancy is caused by the methods using different formulas to calculate the log-fold change values (see the “Methods” section for details). Extreme care should be taken when comparing the log fold-changes returned by Seurat and Scanpy.

Finally, both methods produce spurious results when a gene has exactly zero expression (i.e., zero counts in all cells) in a cluster. When a gene has zero expression, Scanpy’s calculated log-fold change values become spuriously large (Additional file 1: Fig. S28a), while Seurat’s *t*-test method will preferentially up-rank genes that have zero expression even if better candidate marker genes are available (Additional file 1: Fig. S28b).

## Discussion

Marker gene selection is a ubiquitous component of the analysis of scRNA-seq data. However, despite its importance and the large number of methods available, to date there has been no impartial and systematic comparison of competing methods. In this paper, we provided the first comprehensive benchmarking of the available methods. We compared the methods’ performance on a range of metrics and scrutinized the most commonly used methods implemented in the Scanpy and Seurat analysis frameworks. While related to previous work comparing methods for selecting between-cluster DE genes [17], our work focused explicitly on the challenges of finding marker genes and used both a different simulation setting and other different metrics to compare methods.

Overall, across comparisons the best performing methods were those based on the Wilcoxon rank-sum test, Student’s *t*-test or logistic regression. Our results also highlighted that several methods showed systematically worse performance: scran’s findMarkers() and scoreMarkers() methods (especially using the default p-value any method), Cepo, and Welch t-test based methods when both up- and down- regulated genes are selected. In addition, while methods that only selected a subset of genes (RankCorr and NSForest) had excellent specificity they did not show strong predictive performance. Our results also highlighted large differences between the speed and memory consumption of different methods: SMaSH and methods designed for bulk RNA-seq data were particularly memory intensive, while Seurat’s methods were unexpectedly slow. Taken together, these results highlight that more complex methods are not required to select marker genes in scRNA-seq data.

Furthermore, our scrutinisation of Scanpy and Seurat highlighted issues and inconsistencies with their implemented methods. Most significantly, we found that due to an interaction between the surprising existence of zero *p*-values and Seurat’s ranking strategy most of Seurat’s methods will give identical results to ranking by the raw log fold-change values when the number of cells is large. In addition, we identified inconsistencies between how Seurat and Scanpy calculate the log fold-change values that make comparing their output fraught. The existence of these issues highlights the challenges of implementing large analysis frameworks which implement many methodological details. While such frameworks are extremely useful for analysts and contribute greatly to the field, the size of their impact magnifies the downstream effects of any issues in their implementations.

The analysis in this paper suggests a number of directions for further research into improving methodology for selecting marker genes. First, it suggests that attention should be refocused on improving the performance of simple Wilcoxon rank-sum and Student’s *t* tests, which show strong performance even without substantial adaptation to the problem of marker gene selection. Second, our analysis of the DE-based methods’ *p*-values highlights the need for more extensive work on correcting *p*-values for double-dipping. Such work would enable marker genes to be selected based on well-calibrated *p*-value thresholds rather than an ad hoc cutoff to select a given number of genes. Recent work on post-selection inference after clustering represents a promising direction for addressing this issue [53]. Third, specific examples in our analysis highlight that no methods can select marker genes at multiple levels of resolution, for example finding marker genes both for all T cells and specific T cell subsets. Selecting multi-resolution marker genes, likely in tandem with advances in multi-resolution clustering, would allow more precise characterization of cell types in scRNA-seq data.

## Conclusions

We present a systematic and comprehensive comparison of methods for selecting marker genes in scRNA-seq data. Our results highlight a lack of high concordance between methods, large differences in computational demands and large differences in performance, which occur across simulation, expert-annotated gene and prediction-based analyses. Overall, our results suggest that methods based on logistic regression, Student’s *t*-test and the Wilcoxon rank-sum test all have strong performance. On the other hand, scran’s findMarkers(), scoreMarkers(), Cepo and NSForest methods showed generally poor performance across comparisons. Our discovery of unexpected and undocumented behavior in several cases highlights the importance of continued scrutinisation of widely-used methods for scRNA-seq data analysis.

## Methods

### Marker gene selection methods

In this section, we provide more details about the marker gene selection methods compared in this paper.

#### Method parameters

*Seurat*

We change the test.use parameter that controls which two sample statistical test is performed between the cluster of interest and all other clusters. The parameters currently can take the following values:

- wilcox: A Wilcoxon rank-sum test (**Default**);
- t: Welch’s *t*-test ;
- bimod: Likelihood ratio test for single-cell gene expression [27];
- DESeq2: The R package DESeq2 (excluded from comparisons, see below);
- LR: A logistic regression is fit for each gene with a binary cluster of interest indicator as the response;
- MAST: The R package MAST package [19];
- negbinom: A two-group negative binomial GLM;
- poisson: A two-group Poisson GLM;
- roc: For each gene a classifier is built based on that gene’s ability to discriminate between clusters and evaluated using the AUC. The method ranks genes by predictive power $2|\text {AUC} - 0.5|$.

Seurat corrects the *p*-values returned by its methods for multiple testing using Bonferroni’s correction.

*Scanpy*

We change: the test.use parameter, which controls which test is performed, rankby_abs which controls whether genes are ranked by the absolute value of, or the raw score, and tie_correct which controls whether tie correction is performed in the Wilcoxon rank-sum test.

- t-test: Welch’s two-sample t-test (**Default**);
- t-test_over_estimvar: Welch’s two sample t-test where the size of the *rest* group is set to the size of the cluster of interest;
- wilcoxon: A Wilcoxon rank-sum test;
- logreg: Gene-wise logistic regression (excluded from comparisons, see below).

The rankby_abs and tie_correct parameters both take True/False values.

Scanpy corrects the *p*-values returned by its methods for multiple testing using the Benjamani-Hochberg method [54]

*scran*

The *scran* package has two functions for selecting marker genes. We benchamrk both in this manuscript.

findMarkers()

The findMarkers() function uses a pairwise DE approach to select marker genes.

In the function we alter: the test.type parameter, which controls which two-sample statistical test is performed and pval.type which controls how scran aggregates *p*-values. The test.type parameters take the following values:

- t: Welch’s two-sample t-test (**Default**);
- wilcox: Wilcoxon rank-sum test;
- binom: The counts are binarised and an exact binomial test is performed.

The pval.type parameter takes values:

- any: Allows selection of different sized unions of *p*-values between the individual tests;
- all: Combines the *p*-values using an intersection union test [55]; practically the largest of the *p*-values is taken as the overall *p*-value;
- some: A Holm-Bonferroni correction is applied to the *p*-values is applied and then the middle-most *p*-value is taken as the overall *p*-value.

scoreMarkers()

The scoreMarkers() function uses pairwise DE statistic calculation to select marker genes the following statistics are calculated in a pairwise fashion:

- Cohen’s *d* statistic on log fold-change
- AUC
- Log fold-change in detection proportion (the proption of genes that have non-zero expression)

These statistics are summarized across the pairwise comparisons in five ways:

- Mean
- Median
- Maximum
- Minimum
- Minimum rank

Giving 15 different metrics by which to rank genes for marker gene status and ultimately select marker genes.

### Excluded methods

We exclude the following methods from our benchmarking.

*Scanpy’s Logistic regression method*

The implementation was found to not return what is documented, and it is not tested. Seurat’s logistic regression implementation is also included in the benchmarking.

*Seurat’s DESeq2 method*

Seurat’s DESeq2 method was excluded was the comparison after persistent errors getting it to run on the PBMC datasets, apparently due to a low number of counts.

*COMET*

COMET [56] is a marker gene selection method particularly designed to create panels of markers for functional follow-up. COMET uses the XL-minimal Hyper-Geometric test to rank genes [57]. However, COMET’s implementation has problems which make it difficult to benchmark. First, previous (non-neutral) benchmarking has shown that COMET is extremely computationally intensive [26]. (Vargo and Gilbert [26] exclude COMET from their comparisons.) Second, COMET’s Python implementation does not allow the processing of datasets “in memory,” instead requiring multiple text files as input. This requirement means using COMET is challenging, particularly due to highly parallelised nature of our benchmarking workflow. For these reasons we COMET is not included in this manuscript.

#### Self-implemented methods

In addition to the existing methods we implemented several methods:

- Random: A random sample of genes are selected as marker genes. This method provides a “negative control”; it illustrates the worst possible performance in a given scenario.
- Log fold-change sorted by absolute value. First, for each cluster a one-vs-rest log fold-change value is calculated (using Seurat’s formula) for each gene. Second, genes are ranked by the absolute value of the log fold-change. Finally, the top *n* (i.e., with $n = 20$) genes in the ranking are then selected as the marker genes for that cluster. This method is implemented to assess whether ranking by effect size (log fold-change) rather than *p*-value, as for all DE methods, is sufficient to detect marker genes. Ranking by absolute values ensures that both up- and down-regulated genes can be selected as marker genes.
- Log fold-change sorted by absolute value. As above, expect that the genes are ranked by the absolute values of the log-fold change. Ranking by the absolute values means that both up-regulated and down-regulated genes can be selected as marker genes.
- Student’s *t*-test: A one-vs-rest two sample Student’s *t*-test is performed between the cluster of interest and all other clusters for each gene. The top *n* marker genes ranked by the test’s *p*-value are selected as marker genes. This method is implemented to assess whether Student’s *t*-test may be more suitable for selecting marker genes compared to Welch’s *t*-test. The *t*-tests implemented in scran, Scanpy, and Seurat are Welch’s *t*-test. Briefly, Student’s *t*-test assumes that the two samples have the same variance, while Welch’s *t*-test allows the variances to be unequal.

For the log fold-change methods, we implement Seurat’s formula for the log fold-change using the log-normalized counts as input.

#### Other method implementation details

*DE methods*

The edgeR, limma and glmGamPoi methods are all generic methods for DE testing, as such they need additional code to be used to select marker genes. In this paper, all these methods are implemented in a one-vs-rest manner for marker genes. This decision was made based on the poor performance of scran’s methods which implement pairwise testing in early versions of the benchmarking.

*SMaSH*

The implementation of SMaSH uses TensorFlow to train the deep learning models it uses. During training, the weights of the model are written to disk in a file with a fixed name. When the method is run in parallel using Snakemake, multiple instances try to write and read from the same file. To circumvent this issues we forked SMaSH to use a random name for the weights files (https://github.com/jeffreypullin/smash-fork). No other method or implementation details were changed.

#### Out of scope methods

A small number of methods perform analysis tasks similar to, but not the same as, the task of selecting cell-type specific marker genes. These methods are therefore out of scope for the benchmarking performed in this paper.

First, AUCELL [58] analyses geneset activity, not cell-type specific marker genes, in scRNA-seq data. Second, a number of methods, including genesorter [59], scGeneFit [25], SCMarker [60], and scTIM [24], aim to select a single subset of so-called marker genes that best preserve information about the overall cluster structure of the dataset. This aim is generally motivated by the need to reduce the dimensionality of the dataset or to create gene panels for spatial transcriptomics experiments where only a limited number of genes can be assayed. These sets of genes are not cluster-specific and do not distinguish particular cell types, so these methods are out of scope for the benchmarking of cell-type specific marker gene selection methods presented in this paper.

### Computational experiment details

The workflow management tool Snakemake v6.6.1 [61] was used to orchestrate all computational experiments described in the paper. All comparisons were run on the University of Melbourne “Spartan” High Performance Computing cluster which uses the Slurm job scheduler. Analyses were performed in R v4.1.0 [62]. To run methods implemented in Python via R, the reticulate R package [63] and Python v3.8.6 [64] were used. To wrangle data, we used the dplyr [65], tidyr [66] and purrr [67] packages. General purpose visualizations were created using the R package ggplot2 [68] and then assembled into figures using the R package patchwork [69]. The plots that show a comparison of the rankings of multiple methods were created using the rank_rank_plot() function in the topconfects R package [70]. Plots that show the expression of a gene in different clusters were created using the plotExpression() function from the scater R package [71].

### Data processing

All the single-cell datasets were processed in a uniform manner prior to analysis, to remove effects of different preprocessing approaches. All datasets were log-normalized and then subset to the top 2000 highly variable genes (HVGs) using best practices approaches in the Bioconductor ecosystem [28]. For computational reasons, the Ren and Stephenson datasets were down-sampled to 25,000 cells. For all datasets with expert-annotated marker genes, expert-annotated genes that were not selected in the top 2000 HVGs were manually added to the selected genes.

### Concordance measurement and selected marker gene characteristics details

To assess the concordance of the methods for each cluster and dataset we selected the (at most) top 10 selected marker genes for each method. For each cluster and dataset the proportion of genes shared between the result of each method and all other methods was calculated. This proportion was then averaged over cluster and dataset to produce a matrix encoding the average proportion of shared genes between methods. This matrix was converted a distance matrix using dist() function and a hierarchical clustering was calculated with hclust().

To assess the methods generally we calculated the following metrics:

- Cluster mean: the mean of log-normalized expression values in the cluster of interest
- Cluster variance: the variance of log-normalized expression values in the cluster of interest
- Gene mean: the mean of all log-normalized expression values
- Gene variance: the variance of all log-normalized expression values
- Proportion up-regulated the proportion of selected genes that were up-regulated (i.e., had a log fold-change greater than 0)

These metrics were calculated for the at most top 5 genes selected by each method in the all real datasets. To visualize the results the median over clusters and datasets was taken.

To assess the effect size of the selected genes we used the following metrics:

- AUC (i.e., area under the receiver operator characteristic curve using the expression data as a classifier for binary cluster status)
- The absolute value of Cohen’s *d* statistic
- The absolute value of log fold-change

All statistics were calculated in a one-vs-rest manner for the at most top 5 genes selected by each method in the all real datasets. To visualize the results, the median over clusters and datasets was taken and quantile normalization was performed to make the distributions of the three metrics comparable. In the figure, methods were ranked by the median of the quantile normalized values of the three metrics.

To assess the direction of regulation of the method, we selected the (at most) top 20 genes from each method and computed the proportion of these genes that were up-regulated. We defined a gene to be up-regulated if it had a log fold-change, or statistic proportional to the log fold-change, greater than 0. Note that the RankCorr, NSForest, and Cepo methods do not output either a log fold-change or statistic proportional to the log fold-change. For these methods, we calculate a log fold-change (using Seurat’s formula) and add this to output of the method.

To assess the magnitude of the *p*-values produced by the methods, we select the top 40 genes of each method for every cluster in a given dataset and extract the associated multiple testing corrected *p*-value output by the methods. (Note that different methods do use different multiple testing correction methods.) Those *p*-values which took values of exactly 0 were excluded. Seurat’s ROC method, NSForest, Cepo, COSG, RankCorr, and (self-implemented) log fold-change and random methods were all excluded from this analysis as they do not produce *p*-values. In the Zhao dataset, all top 40 genes for the Seurat (Poisson GLM) method have exactly-zero *p*-values so the method was excluded from plotting.

To assess the exactly-zero *p*-values produced by the methods we calculated the number of exactly-zero *p*-values produced by each method in every cluster in all datasets. To plot the data, the we took the median over clusters for each method and dataset. No limit was set on the number of top marker genes taken as it was observed that in some datasets methods could produce hundreds of exactly-zero *p*-values. Again, methods that do not produce *p*-values (see above) were excluded from the analysis.

### Simulated performance assessment

Datasets were simulated using the splatter model implemented in the Splatter package [48]. The splat model is designed to be a general purpose simulator of scRNA-seq data. In this paper, we adapt it to simulate marker genes by designing and implementing a gene-wise marker gene selection and ranking strategy based on the simulation parameters of the model. This method is designed to select and up-rank genes which visually appear to be marker genes and match our holistic view of the characteristics of good marker genes.

#### Simulated marker genes selection strategy

A key component of the splatter model is the set of DE factors for each group and each gene. Let $g \in \{1, \dots , G\}$ index genes and $k \in \{1, \dots K \}$ index groups. Then $\beta _{gk}$ is the DE indicator for the *k*th group and *g*th gene. In the splat model $\beta _{gk}$ encodes the amount of DE the *g*th gene shows in the *k*th group relative to a background level of expression. In this context $\beta _{g'k'} = 1$ would indicate background expression for gene $g'$ in group $k'$.To assess whether each gene is a marker gene for a particular cluster we use the following original score, $m_g$. Let $k'$ be the index of the particular cluster of interest, then:

1 $$\begin{aligned} m_g = \frac{1}{K - 1} \sum \limits _{i = 1, i \ne k'} \log \left( \frac{\beta _{gk'}}{\beta _{gi}}\right) . \end{aligned}$$

This score is the mean of the log-ratio of the DE factor in the cluster of interest versus all other clusters. In practice, the score can consistently up-rank genes that have large log fold-changes. However, if the overall mean of the gene is small the genes do not visually appear to be marker genes. We therefore filter to only genes which show sufficient mean expression: in practice we have found that only keeping genes with gene mean $> 0.1$ works well.

Our overall strategy for selecting simulated marker genes for a specific cluster from the simulated data consists of:

1. Filter out genes which have simulated mean expression $< 0.1$;
2. Calculate the marker gene score $m_g$ for each gene;
3. Rank genes by $m_g$;
4. Select the top *n* genes as marker genes;

for some suitable small *n*. Compared to other methods for selecting marker genes such as the strategy used in the original Splatter paper this score is less restrictive, better accommodating cases where multiple DE factors are not 1 but the gene is still a strong marker gene. The strategy described above can, in theory, be used to select both and up- and down-regulated marker genes. In this paper, however, we only use it to select up-regulated marker genes. While we have many examples of true up-regulated marker genes (all of the expert-annotated maker genes used in this paper are up-regulated) it is not clear what the ideal expression profile of a down-regulated marker gene is. Indeed, when we used the above strategy to select down-regulated marker genes we found that their expression patterns did not visually appear to be marker genes.

#### Simulation parameters

To perform simulations all estimable parameters were estimated from all datasets. To account for the fact that the splat model’s parameter estimation does not account for clustering in the data we filtered each dataset to only one cluster and then performed the parameter estimation on those cells. The cluster used for each dataset is recorded in Table S8 in Additional file 1. Clusters were mainly chosen based on size, with larger clusters preferred, and to avoid representing an ‘unusual’ cell type. For the three PBMC datasets a B cell cluster was chosen for consistency. Splatter cannot, however, estimate parameters that control the amount of DE between clusters from data. There are two such parameters: the location and scale parameters for the DE factor. In the experiments presented in the main paper we take these parameters to be 3 and 0.2, respectively. These values were chosen as they produced marker genes with similar expression profiles to those observed in real datasets (Additional file 1: Fig. S9). Additional simulations suggest that the results of method comparison are similar for different values of the location parameter (Additional file 1: Fig. S11). Note that in all of these analyses we fix the value of the scale parameter; this is because the parameter controls the variance of the (lognormal) distribution and so has less impact on the amount of DE. The dropout and outlier gene probabilities in the splat model were both set to zero. Each simulated dataset was replicated 3 times using a different seed for randomization. 

#### Simulation performance measurement

To measure the performance of the methods we used the following strategy for each cluster in each (replicated) simulated dataset. We selected the (at most) top 20 selected marker genes for each method and the top 20 simulated marker genes. A gene was a true positive if it occurs in both lists, a false positive if it occurs in the list of selected genes but not in the list of simulated genes, a false negative if it does not occur in the selected list of genes but does appear in the simulated list, and a true negative if occurs in neither of the lists. Using these definitions we calculate the number of true positives (TP), false positives (FP), and false negatives (FN) and calculate:

$$\begin{aligned} \text {Recall} = \frac{\text {TP}}{\text {TP} + \text {FP}}, \ \text {Precision} = \frac{\text {TP}}{\text {TP} + \text {FN}}, \ \text {F1 score} = 2 \cdot \frac{\text {Recall} \cdot \text {Precision}}{\text {Recall} + \text {Precision}} \end{aligned}$$

Note that with these definitions the number of simulated genes = $\text {TP + FP}$ and the number of selected genes = $\text {TP} + \text {FN}$. To summarize the performance of methods for each simulation scenario (i.e., each dataset), we calculated the median recall, precision and F1 score across clusters and simulation replicates.

### Stability simulations

To assess the stability of methods we performed simulations by down-sampling either the number of clusters or the number of cells in the exemplar pbmc3k dataset. Cells were down-sampled by randomly removing 25%, 50%, and 75% of all cells. Clusters were removed one-by-one until only two clusters remained. The ordering of clusters for removal was: Naive CD4 T, CD14+ Mono, Memory CD4 T, Platelet, CD8 T, FCGR3A+ Mono, NK, DC, B. All methods were run and the at most top 10 marker genes were retained. Method stability was assessed by calculating the proportion of genes that were the same as in the full pbmc3k dataset, taking the median over the clusters.

### Expert-annotated marker gene details

To assess the ability of the methods to recover expert-annotated marker genes we use the following approach. First, we identified sets of expert-annotated marker genes for the Zeisel, Lawlor, pbmc3k, Smart-seq3, Ren, Stephenson, Yoshida, and Dominguez datasets:

- *pbmc3k*: The expert-annotated marker genes were taken from the “Seurat - Guided Clustering Tutorial” (Additional file 1: Table S3);
- *Lawlor*: The expert-annotated marker genes were taken from the paper describing the dataset (see the fifth paragraph) [38]. In the paper, these genes were used to annotate the defined clustering (Additional file 1: Table S4);
- *Zeisel*: The expert-annotated marker genes were taken from the paper describing the dataset (see the fourth paragraph). We exclude the “None/Other” from expert-annotated marker gene analyses as it does not have marker genes in the original paper [72]. (Additional file 1: Table S5);
- *Smart-seq3*: The expert-annotated marker genes were taken from the paper describing the dataset [42]. Specifically, these genes were taken from Extended Data Fig. 10. Note that not all the marker genes identified in this figure are used. Marker genes for clusters which easily mapped to cluster present in the final version of the clustering were preferred (Additional file 1: Table S6)
- *Harmonized blood datasets (Ren, Stephenson, Yoshida and Dominguez Conde datasets)*: The expert marker genes for these datasets were taken from Version 2 of the CellTypist immune cell atlas [43, 47] which was created by the same group that performed the harmonization. Note that each dataset contains a different set of cell types, but with substantial overlap (Additional file 1: Table S7)

Then, to measure the performance of the methods, we select marker genes for all clusters in all dataset with expert-annotated marker genes (the number of top genes used was chosen based on the number of expert-annotated marker genes). For each cluster in each dataset the recall of the method was calculated (see above for more information about how this was defined and calculated).

### Predictive performance measurement

To assess the predictive performance of the methods, we used the following strategy. For each real dataset we selected the (at most) top 5 marker genes for every cluster, and combined them to give an overall set of genes for each dataset. Three classifiers were applied:

- A KNN classifier, with $k = 3$,
- An SVM classifier, with liner kernel, and,
- A ‘maximum sum’ classifier, described below.

Specifically, we used the knn function from the FNN R package [73] to implement the KNN classifier and the function svm from the e1071 [74] package to implement the SVM. The maximum sum classifier was implemented from scratch. For this classifier, the expression values of the marker genes for each cluster were centered and scaled and then summed across genes. This process results in a vector of summed expression values across clusters for each cell. The cell is then classified as the cluster with the largest summed expression value.

To assess the performance of the classifier 5-fold cross-validation was used. Each dataset (for each method) was divided into 5 approximately equal groups (i.e., approximately 20% of the total cells) using a sample stratified by cluster. The stratified sample was used to prevent folds having no examples of rare cell types. The model was then fit 5 times with each of the groups being used as the test set while the other groups were pooled to give the training set. As the maximum summed expression classifier had no tunable parameters it was just assessed on the test sets. Method performance was summarized using the F1 score of the resulting classifier. See above for the mathematical definition of the F1 score. To further summarize the results the median F1 score of the method, dataset pair across clusters was calculated.

### Speed and memory measurement

To measure method speed, we used the R system.time() function, extracting the elapsed time. To measure the memory usage, we used the benchmark functionality from Snakemake, which uses the ps command to measure the memory of the submitted jobs. Specifically, we report max_vss as the memory. Note that for methods implemented in Python both the time and memory measurements take into account the overhead of converting between R and Python using the reticulate package. The memory measurement also includes various data and output wrangling tasks needed to run specific methods.

### Implementation assessment

To compare the quality of the methods’ implementations we assessed implementation as Good, Adequate, or Poor based on five criteria: accessibility (how easy is the software to access, i.e., where is it hosted?), installation (how easy is the software to install?), documentation (what is the quality of documentation?), ease of use (how is easy is the software to use for selecting marker genes?) and interpretability of output (how easy is the output to interpret and use?). For further details of how each criteria was rated as Good Adequate or Poor see the Supplementary Methods.

### Log fold-change calculation methods

Let $Y_{ig}$ be the log-normalized expression value for cell *i* and gene *g*, $G_1$ and $G_2$ be the indices of the cells in the cluster of interest and all other clusters respectively, and $n_1$ and $n_2$ give the number of cells in the two groups. Then, for a given cluster of interest, Scanpy and Seurat calculate the log-fold change for gene *g* as:

#### Seurat

 

$$\begin{aligned} f_g = \log _{2}\left( \frac{1}{n_1}\sum \limits _{i \in G_1} \left( \exp (Y_{ig}) - 1\right) + 1 \right) - \log _{2}\left( \frac{1}{n_2}\sum \limits _{i \in G_2} \left( \exp (Y_{ig}) - 1\right) + 1 \right) . \end{aligned}$$

Note that’s Seurat’s Poisson and Negative Binomial GLM methods calculate the log fold-change based on the raw count, that is the above formula applies with $Y_{ig}$ being the observed count for cell *i* in gene *g*.

#### Scanpy

 

$$\begin{aligned} f_g = \log _{2}\left( \exp \left( \frac{1}{n_1}\sum \limits _{i \in G_1} Y_{ig}\right) - 1 + \epsilon \right) - \log _{2}\left( \exp \left( \frac{1}{n_2}\sum \limits _{i \in G_2} Y_{ig}\right) - 1 + \epsilon \right) . \end{aligned}$$

where $\epsilon = 10^{-9}$. Scanpy’s documentation notes that the log-fold change values are “an approximation calculated from mean-log values” This refers to how the inversion of the $\log (x + 1)$ transformation of the log-normalized counts by $\exp (x) - 1$ is done on the mean of the log-transformed counts, not the log transformed counts themselves. In Scanpy’s implementation the means of the log transformed data are precalculated, so this approximation is probably designed to exploit this feature.

### Relationship between score-based and *p*-value-based ranking

This equivalence follows from the property that cumulative distribution functions are monotonically increasing. For a two-tailed test of the null hypothesis $H_0: \mu = 0$ which produces a *z*-score, *z*, the *p*-value, *p*, is calculated as

$$\begin{aligned} p = 2 (1 - \Phi (|z|)), \end{aligned}$$

where $\Phi (\cdot )$ is the CDF of the standard normal distribution. If there are two scores, $z_1$ and $z_2$ say, with $|z_1| < |z_2|$, then from the fact that $\Phi (\cdot )$ is monotonically increasing it can be derived that:

$$\begin{aligned} |z_1|{} & {} < |z_2| \\ \implies \Phi (|z_1|){} & {} < \Phi (|z_2|) \\ \implies 2(1 - \Phi (|z_1|)){} & {} > 2(1 - \Phi (|z_2|)) \\ \implies p_1{} & {} > p_2 \end{aligned}$$

where the first inequality follows from the increasing monotonic property of the CDF. This argument shows that ranking by the absolute value of the z-score (in decreasing order) will be the same as ranking by the *p*-values (in increasing order) for tests that return a z-score.

This equivalence does not hold, however, for the Welch’s* t*-test (which is the default statistical test used by Scanpy). In Welch’s *t*-test, the degrees of freedom ($\nu$) of the *t*-distribution that the *t*-value is compared is dependent on the variance of the data. Different genes will therefore have different $\nu$ values so each *p*-value is calculated from a different instance of the t-distribution and the mathematical argument presented for ranking by *z*-score does not hold.

### Differential expression testing strategy

Different methods based on DE testing use different strategies to perform the testing. When DE testing is used to identify marker genes we need to assess which genes are different in the cluster of interest (i.e., the cluster we wish to select marker genes for) relative to the other clusters present in the data. The *one-vs-rest* and *pairwise* testing strategies are different approaches for quantifying this difference.

*one-vs-rest*

In the one-vs-rest approach, the cells in all other clusters and pooled and a statistical test is performed between the cluster of interest and the pooled ‘rest’ cluster for each gene. This method is implemented by Scanpy and Seurat and we the approach we use this approach to implement edgeR, limma and glmGamPoi for marker gene selection in this paper.

*pairwise*

In the pairwise approach, a statistical test is performed between the cluster of interest and each other cluster for each gene. This gives $K - 1$ *p*-values (or statistics) for each gene, cluster of interest combination, where *K* is the number of clusters in the dataset. To get an overall measure of significance for each gene the *p*-values (or statistics) need to be combined in some way. This method is implemented in scran. The scran findMarkers() function performs pairwise DE testing, while the scoreMarkers() function calculates a statistic measuring DE in pairwise fashion. The rationale for this more complex DE approach is that the multiple *p*-values provide more information and that it is hypothetically less affected by the relative sizes of different clusters [75].

### Supplementary information

**Additional file 1:** Supplementary text (Method implementation criteria), **Tables S1-5**, (expert annotated marker genes), **Table S6** (cluster for simulation), **Figs. S1-2** (method concordance), **Fig. 3** (*p*-values), **Fig. S4** (gene means across datasets), **Figs. S5-7** (marker genes), **Figs. S8-9** (simulated data), **Figs. S10-12** (simulation comparison) **Figs. S13-16** (expert-annotated comparison), **Fig. 17** (PECAM expression in Smart-seq3 dataset), **Figs. S18-19** (predictive performance comparison). **Figs. 20-21** (speed and memory comparison), **Figs. 22-27** (data characteristics impact comparison), **Fig. 28** (exactly 0 expression case study).

**Additional file 2.** Review history.

## Data Availability

All code used in the analyses presented in this paper is available on GitLab, under a MIT licence, https://gitlab.svi.edu.au/biocellgen-public/mage_2020_marker-gene-benchmarking ([76]) with a snapshot on Zenodo [77]. The raw datasets were accessed from the scRNAseq R package (Lawlor, Zhao and Zeisel) [78], scanpy (Paul), the TENxPBMCData R package (pbmc3k) [37], the Gene Expression Omnibus (Endothelial, Mesenchymal, CITE-seq), ArrayExpress (Astrocyte, Smart-seq3), and the Cell Typist Blood organ atlas (Yoshida, Ren, Stephenson and Dominguez-Conde). Specifically, data from the following studies and accession numbers was used: Zeisel et al. [72] (GSE60361 [36]), Lawlor et al. [38] (GSE86469 [79]), Natri et. al. [39] (Endothelial and Mesenchymal datasets in text) (GSE227136 [80]), Kleshchevnikov et al. [8] (Astrocyte in text) (E-MTAB-11115 [81]), Paul et al. [40](GSE72857 [82]), Hagemann-Jensen et al. (Smart-seq3 in text) [42] (E-MTAB-8735 [83]) Hao et al. [12] (CITE-seq in text) (GSE164378, [84]), Zhao et al. [41] (GSE125188, [85]), Dominguez-Conde et al. ([47]) (E-MTAB-11536 [86]) Stephenson et al. [44] (E-MTAB-10026 [87]), Ren et al. [45] (GSE158055 [88]) and Yoshida et al. [46] (GSE168215 [89]). Processed data files (in .rds format) are available from Zenodo (10.5281/zenodo.6513335, [90]).
