# Supplementary material for: A comparison of marker gene selection methods for single-cell RNA sequencing data
Source: Genome Biol. 2024 Feb 26;25:56. doi: 10.1186/s13059-024-03183-0 (PMC10895860; doi:10.1186/s13059-024-03183-0)
Supplement: Supplementary file 1 — Additional file 1: Supplementary text (Method implementation criteria), Tables S1-5, (expert annotated marker genes), Table S6 (cluster for simulation), Figs. S1-2 (method concordance), Fig. 3 (p-values), Fig. S4 (gene means across datasets), Figs. S5-7 (marker genes), Figs. S8-9 (simulated data), Figs. S10-12 (simulation comparison) Figs. S13-16 (expert-annotated comparison), Fig. 17 (PECAM expression in Smart-seq3 dataset), Figs. S18-19 (predictive performance comparison). Figs. 20-21 (speed and memory comparison), Figs. 22-27 (data characteristics impact comparison), Fig. 28 (exactly 0 expression case study). [file 13059_2024_3183_MOESM1_ESM.pdf]

Supplementary material for: A comparison of marker gene selection methods for single-cell RNA sequencing data

Jeffrey M. Pullin[1,2,3], Davis J. McCarthy[1,2,3,*]

[1]*Bioinformatics and Cellular Genomics, St Vincent's Institute of Medical Research, Fitzroy, Australia*
[2]*School of Mathematics and Statistics, Faculty of Science, University of Melbourne, Parkville, Australia*
[3]*Melbourne Integrative Genomics, Faculty of Science, University of Melbourne, Parkville, Australia*
[*]*Corresponding author*

January 1, 2024

Supplementary methods

Implementation criteria

The criteria used to rate the implementations of the methods.

- Accessibility.
  - Good: The software is hosted in standard repository (e.g. Bioconductor, pip)
  - Adequate: The software is only hosted in a standard Git server
  - Poor: The software is hosted in a non-standard way or is not accessible
- Installation. How easy is the software to install?
  - Good: The software can be installed in a standard way.
  - Adequate: The software can be installed in a non-standard way.
  - Poor: The software can only be installed with difficulty or not at all.
- Documentation quality. How well documented is the software?
  - Good: There is substantial documentation
  - Adequate: There is some documentation; enough to run the software.
  - Poor: There is little or no documentation.
- Ease of use. How easy is it to run the software to select marker genes?
  - Good: The package natively supports selecting marker genes and common scRNA-seq data formats are supported.
  - Adequate: The package natively supports selecting marker genes but the passed data is is required in an odd format.
  - Poor: Additional code must be written to support the selection of marker genes
- Quality of output. How easy is it to use and interpret the output?
  - Good: The output is in a sensible format with all expect components
  - Adequate: The output is in a mostly sensible format, possibly with some components mangled.
  - Poor: The output is in an odd, difficult to use format.

Supplementary figures and tables

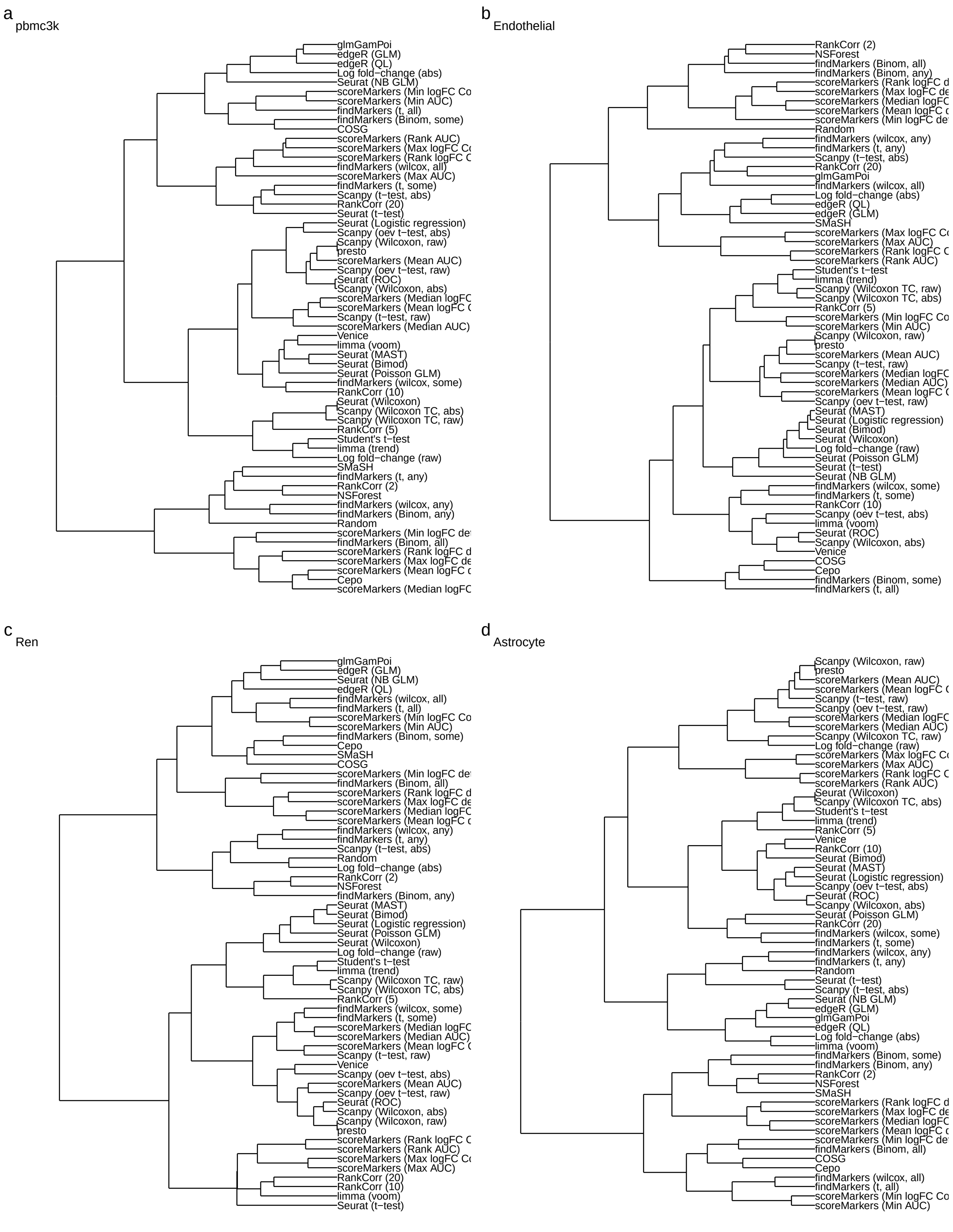

Figure S1: Concordance clustering selected datasets. The dendrograms are created as described in the main text and Methods. a) pbmc3k dataset, b) Endothelial dataset c) Ren dataset d) Astrocyte dataset

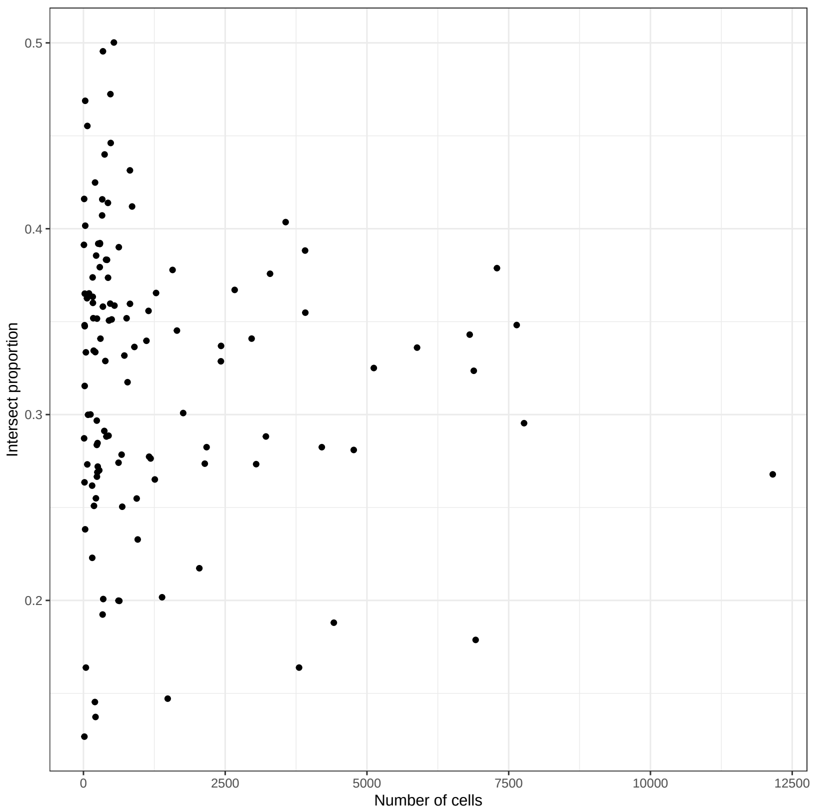

Figure S2: Intersection of methods output proportion by number of cells in cluster. The intersect proportion for a cluster is calculated by averaging over the intersection for each pair of methods

Table S1: Expert marker genes: pbmc3k dataset

Cell type	Marker genes
Naive CD4+ T	IL7R, CCR7
CD14+ Mono	CD14, LYZ
Memory CD4+	IL7R, S100A4
B	MS4A1
CD8+ T	CD8A
FCGR3A+ Mono	FCGR3A, MS4A7
NK	GNLY, NKG7
DC	FCER1A, CST3
Platelet	PPBP

Table S2: Expert marker genes: Lawlor dataset

Cell type	Marker gene
Beta	INS
Stellate	COL1A1
Ductal	KRT19
Alpha	GCG
Acinar	PRSS1
Gamma/PP	PPY
Delta	SST

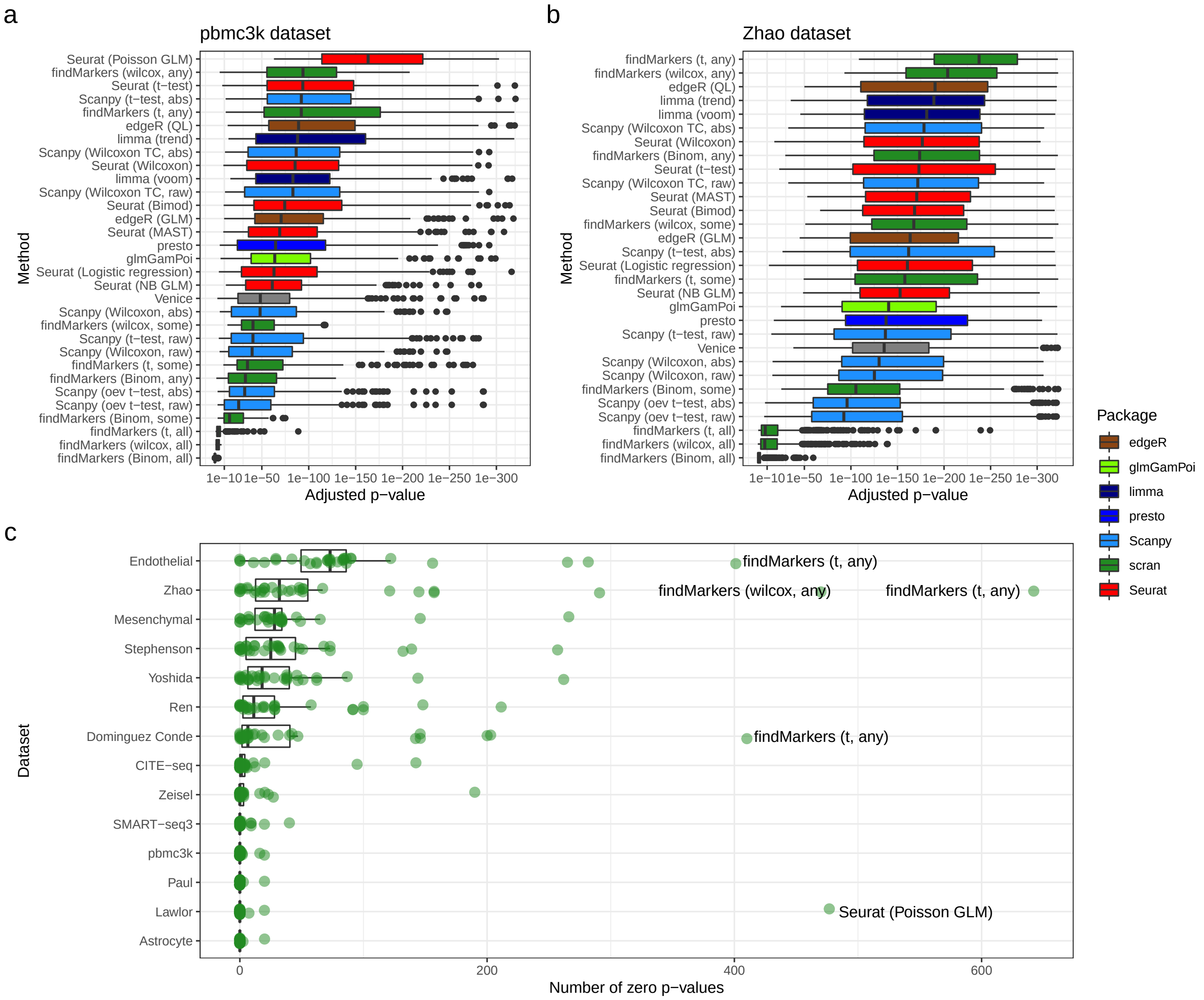

Figure S3: p-value magnitudes and exactly-zero p-values. a) Boxplots of (multiple correction) adjusted p-values for all methods which output p-values run on the pbmc3k dataset (results for all clusters included in each boxplot). b) As in a) for the Zhao dataset. Different methods use different methods to perform multiple testing correction, see Methods. c) The number of genes for each method in each dataset (median over clusters) that returned p-values of exactly 0. Methods that did not return p-values were not included in this analysis.

Table S3: Expert marker genes: Zeisel dataset

Cell type	Marker gene
interneurons	PNOC
pyramidal SS	TBR1
pyramidal CA1	SPINK8
oligodendrocytes	HAPLN2
microglia	AIF1
endothelial-mural	ACTA2
astrocytes_ependymal	ALDOC

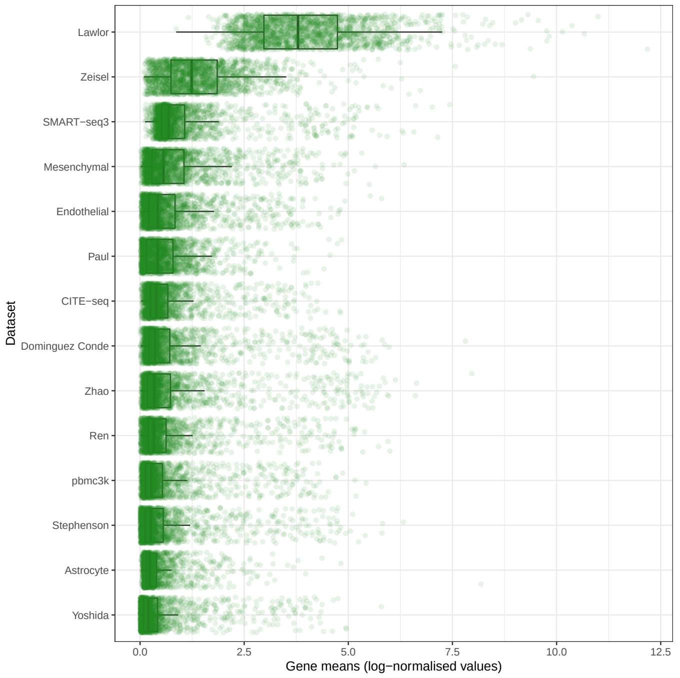

Figure S4: Gene means across datasets. The distribution of gene means across datasets calculated using log-normalised counts. The Lawlor dataset has substantially higher mean expression compared to other datasets.

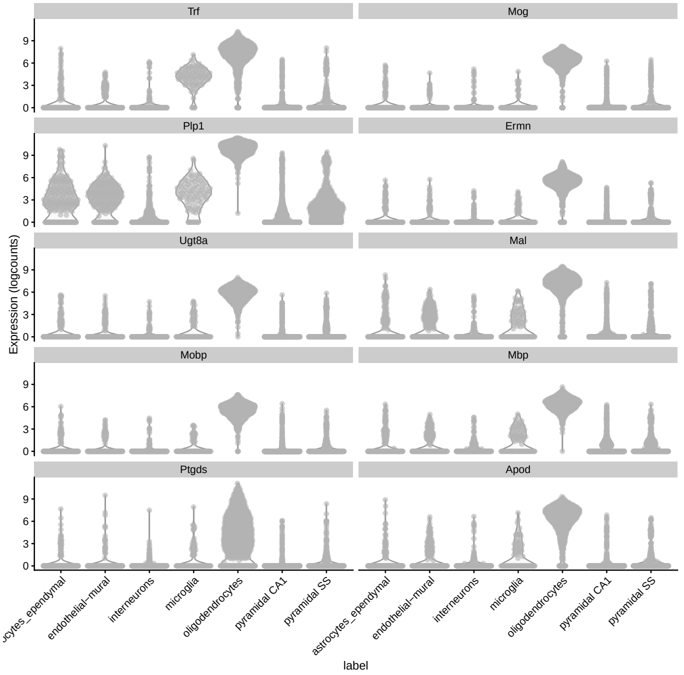

Figure S5: Ten top marker genes selected by the Seurat (Wilcoxon) method in the Oligodendrocyte cluster, Zeisel dataset

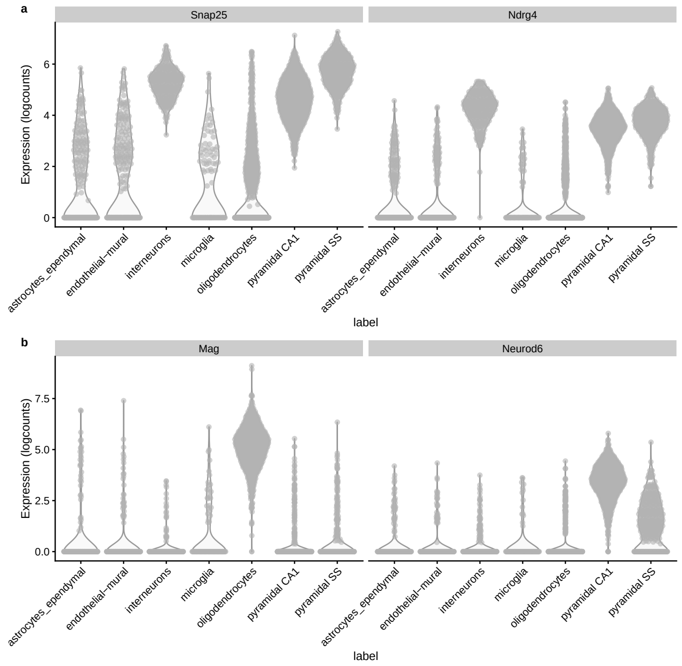

Figure S6: Top two marker genes selected by the Seurat (t-test) and scran t-test-any method in the Interneuron cluster, Zeisel dataset a) Marker genes selected by Seurat t-test. b) scran t-test any

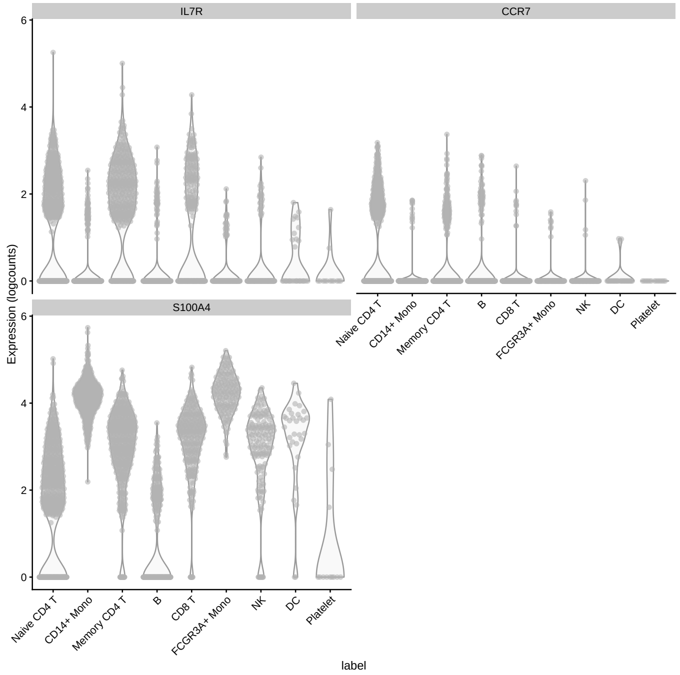

Figure S7: Expert annotated marker genes for the CD4 T Memory and CD4 T Naive cells in the pbmc3k dataset *IL7R* is annotated as a marker gene for both clusters

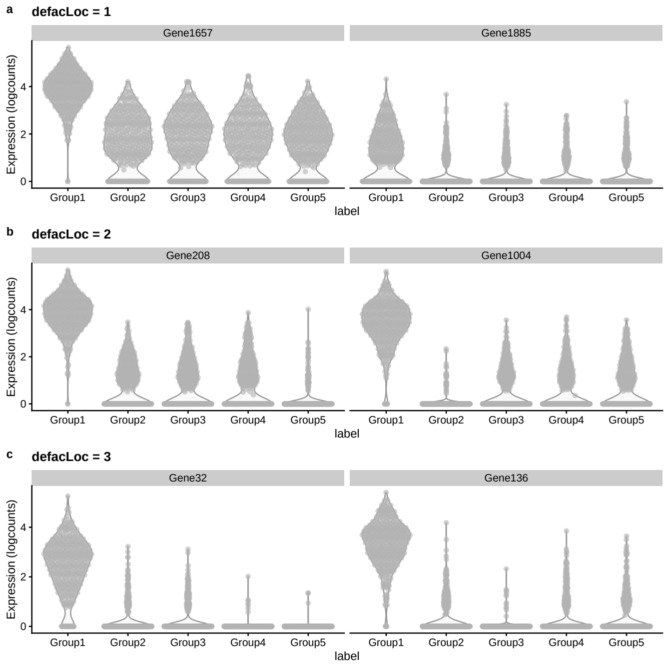

Figure S8: Top 2 'true' marker genes for simulation with different values of `de.facLoc` All simulations have all other parameters estimated from the pbmc3k dataset and have `de.facScale` = 0.2. The `de.facScale` parameter controls the variance of the distribution of DE factors and therefore has less of an effect on the simulations.

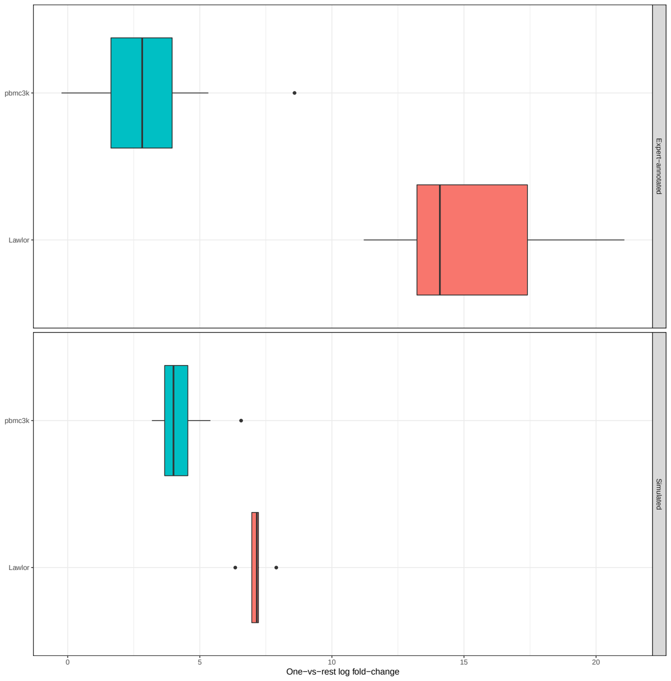

Figure S9: Log fold-change values for the top simulated and expert-annotated genes in the pbmc3k and Lawlor datasets. The simulated and expert-annotated marker genes have similar log fold-change values, and the simulations are able to partially recapitulate the differing magnitude of the log fold-changes in the two datasets. To make the number of simulated and expert-annotated marker genes similar, the top 3 simulated marker genes for the pbmc3k dataset and the top simulated marker genes for the Lawlor dataset are used.

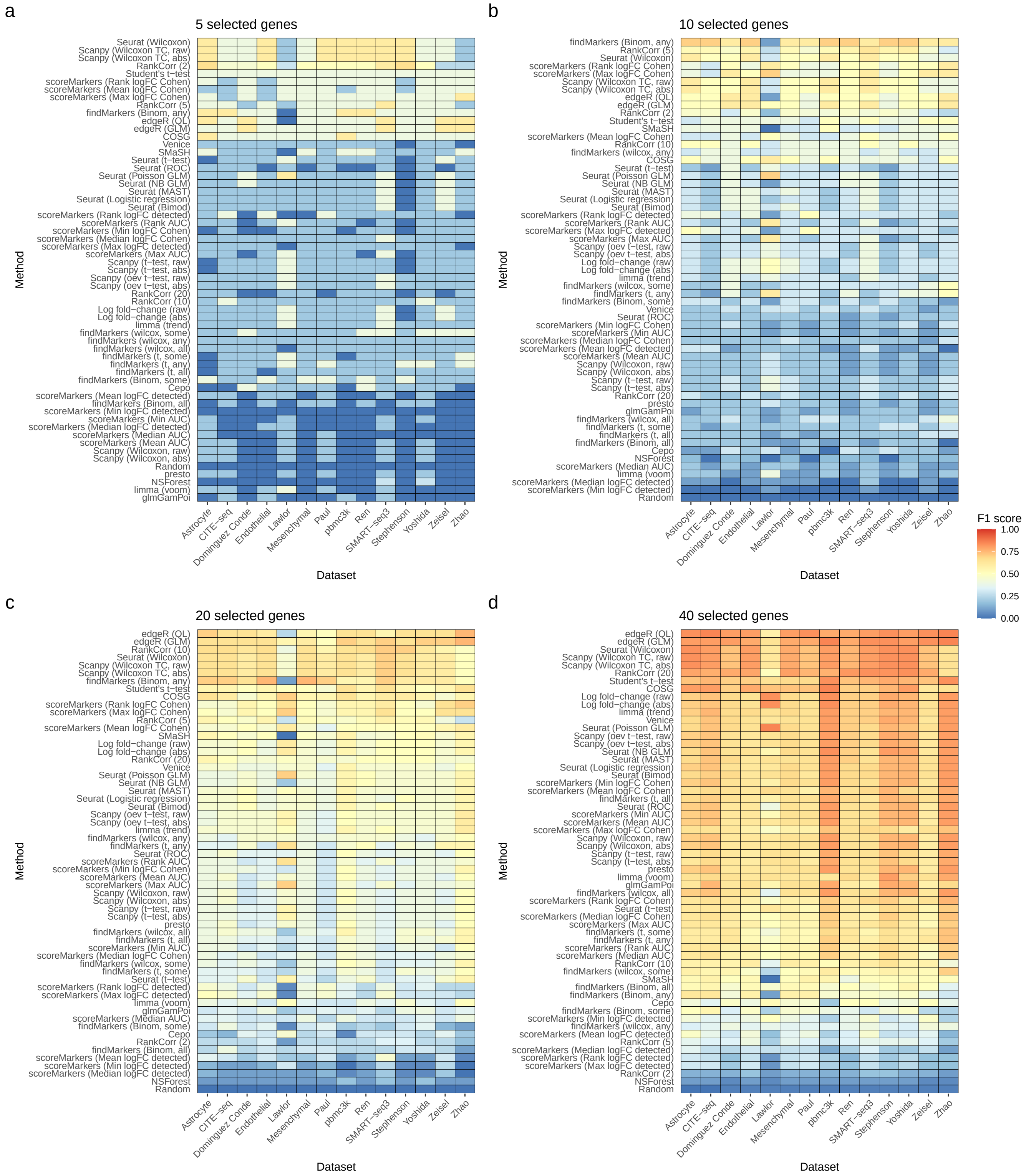

Figure S10: F1 scores across simulations scenarios when the number of selected and true marker genes is varied a) 5 genes are selected and used as true marker genes. b) 10 genes. c) 20 genes. d) 40 genes

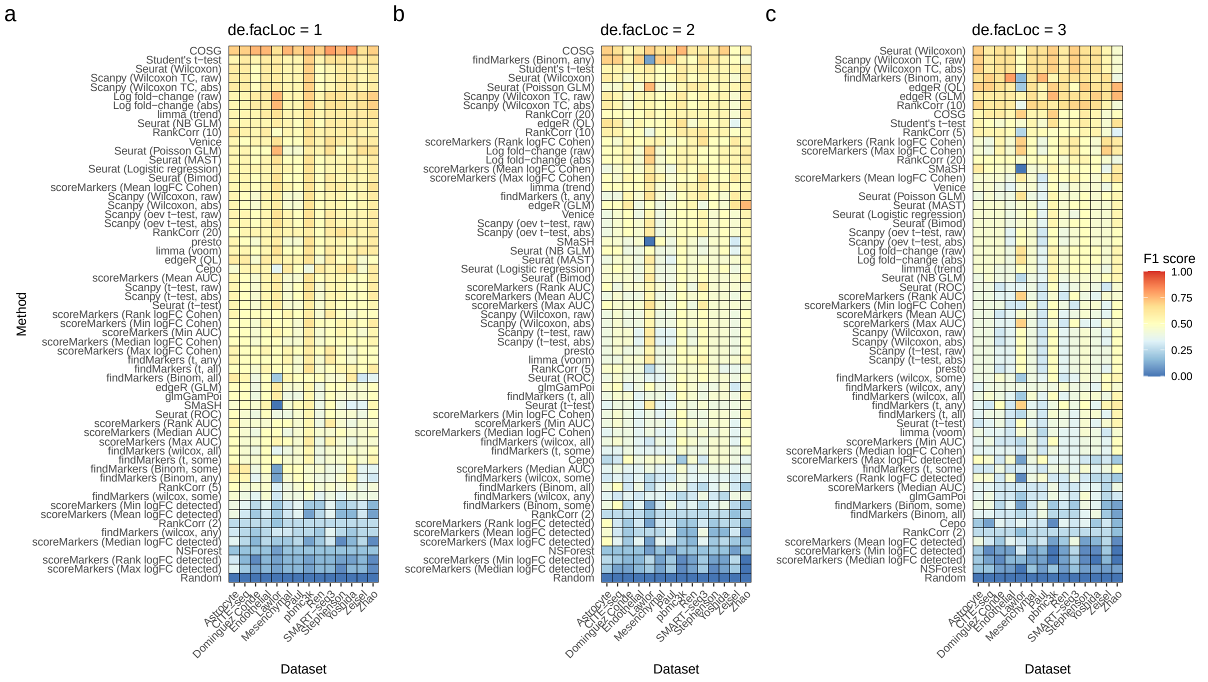

Figure S11: Simulation results using different values of splatter de.facLoc parameters Results are relatively concordant for different values of the parameter. a) de.facLoc = 1, b) de.facLoc = 2, c) de.facLoc = 3. All simulations have `de.facScale` = 0.2

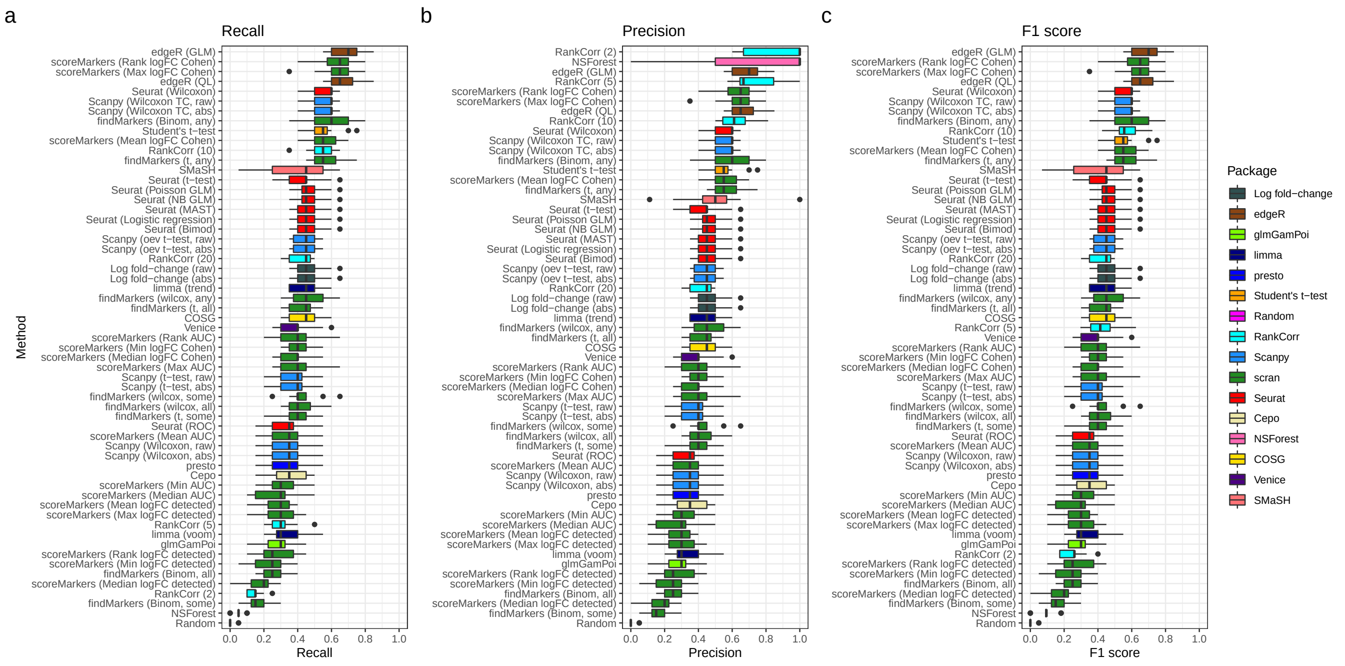

Figure S12: Performance of all methods on simulated data in the Zeisel-based simulation scenarios Boxplots show variations in performance across clusters and simulation replicates. a) Recall. b) Precision. c) F1 score.

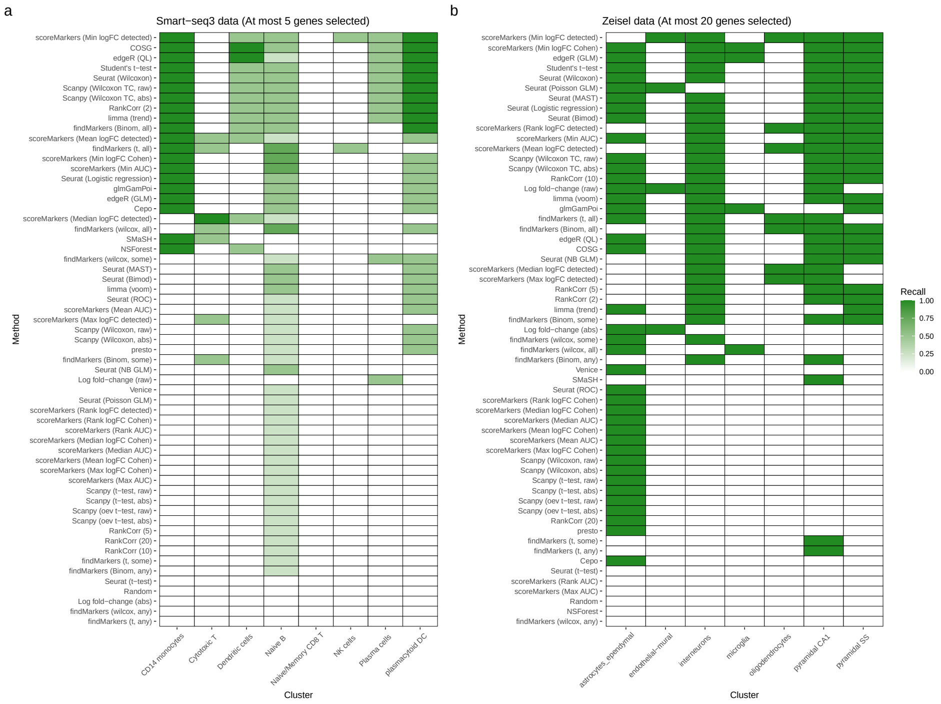

Figure S13: Recall of methods on the Smart-seq3 and Zeisel datasets when recovering expert-annotated marker genes a) Results for the Smart-seq3 dataset. At most 5 genes were selected and the expert-annotated marker genes were taken from the supplementary material of the paper describing the Smart-seq3 method. b) Results for the Zeisel datasets. At most 20 genes were selected and the expert-annotated marker genes were taken from the paper describing the Zeisel dataset.

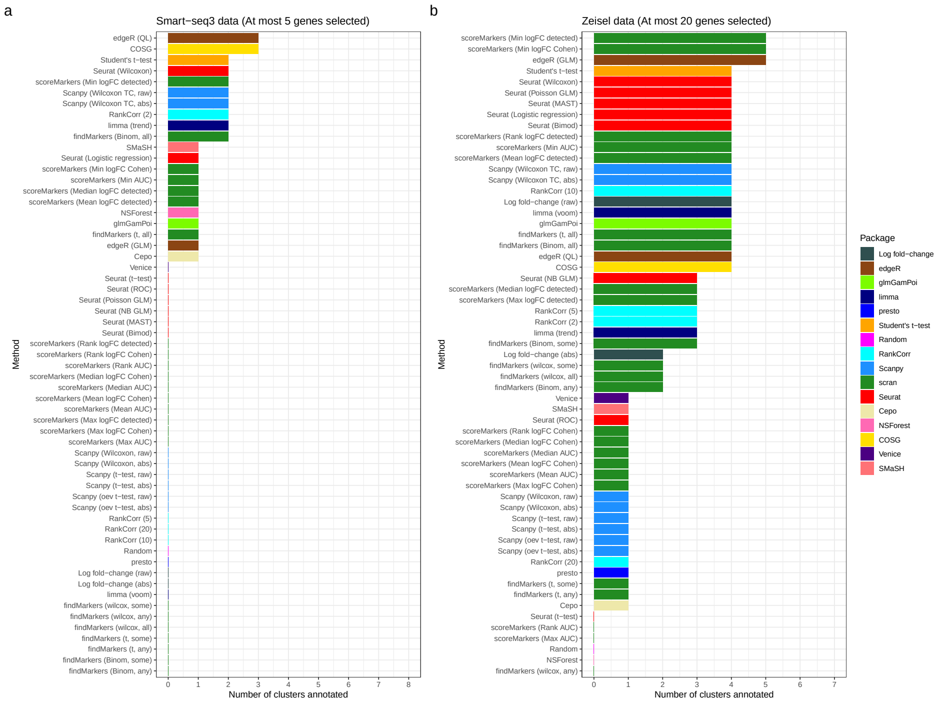

Figure S14: Number of clusters annotated by methods for the Smart-seq3 and Zeisel datasets using expert-annotated marker genes A cluster was taken to be successfully annotated if the method was able to recover all of its associated expert-annotated marker genes. a) Results for the Smart-seq3 dataset. At most 5 genes were selected and the expert-annotated marker genes were taken from the supplementary material of the paper describing the Smart-seq3 method. b) Results for the Zeisel datasets. At most 20 genes were selected and the expert-annotated marker genes were taken from the paper describing the Zeisel dataset.

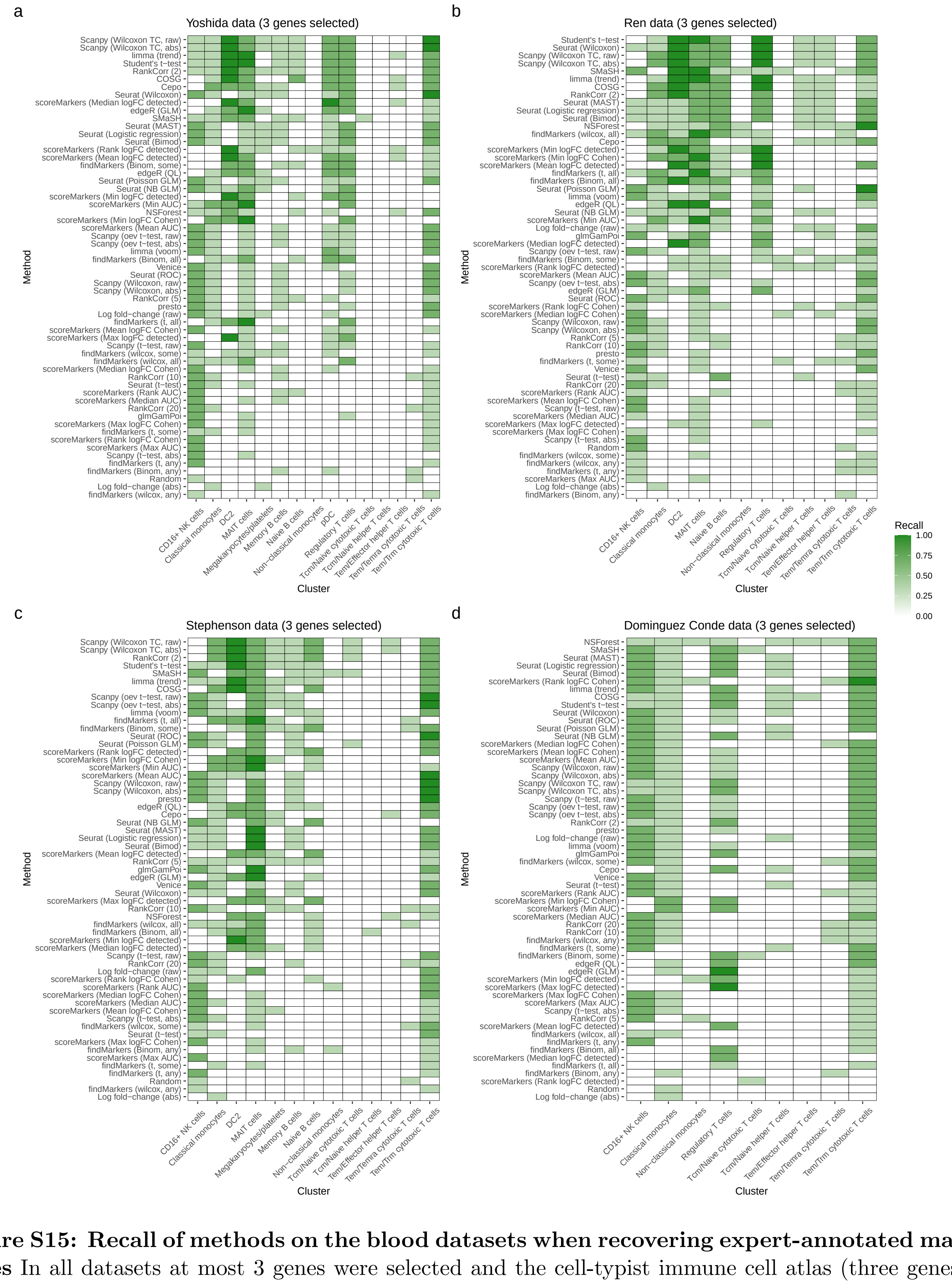

Figure S15: Recall of methods on the blood datasets when recovering expert-annotated marker genes In all datasets at most 3 genes were selected and the cell-typist immune cell atlas (three genes per cell-type) was used as the source of the expert-annotated marker genes. a) Yoshida dataset b) Ren dataset c) Stephenson dataset d) Dominguez Conde dataset

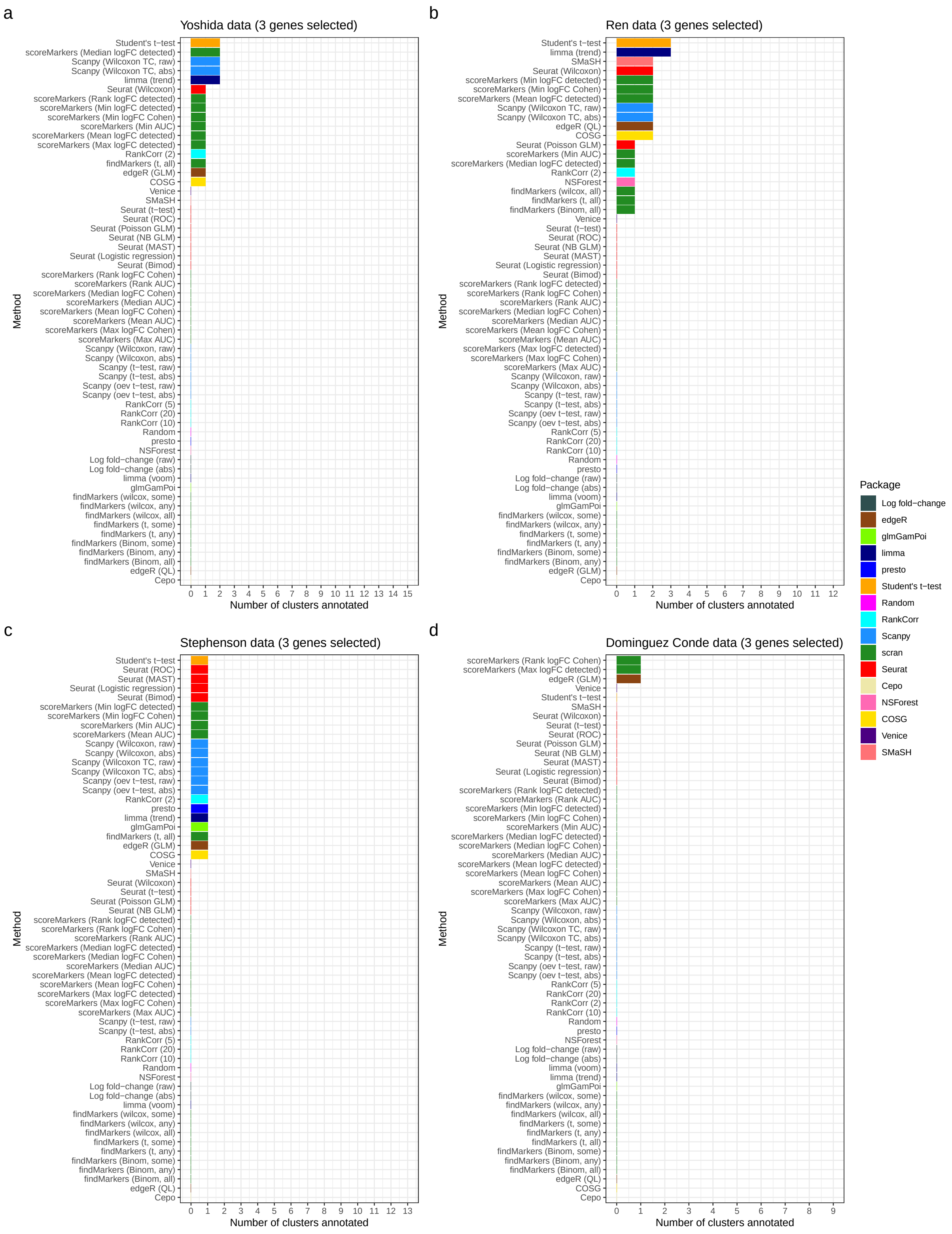

Figure S16: Number of clusters annotated by methods for the blood datasets using expert-annotated marker genes A cluster was taken to be successfully annotated if the method was able to recover all of its associated marker genes. In all clusters at most 3 genes were selected and the cell-typist immune atlas was used as the source of the expert-annotated marker genes a) Yoshida dataset b) Ren dataset c) Stephenson dataset d) Dominguez Conde dataset

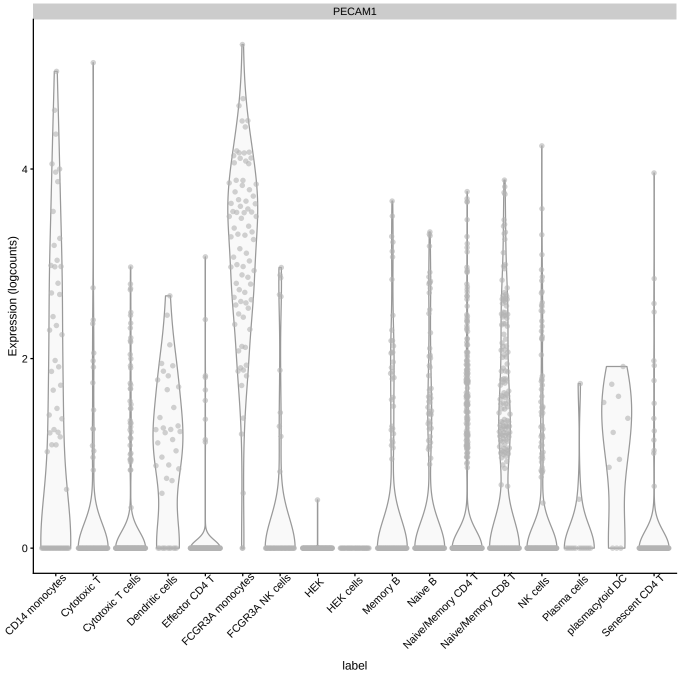

Figure S17: Expression of the *PECAM1* gene across clusters in the Smart-seq3 dataset

Table S4: Expert marker genes: Smart-seq3 dataset

Cell type	Marker gene
NK cells	NCAM1, KLRB1
Naive/Memory CD8 T	PECAM1
Cytotoxic T	GZMB, GZMA
Naive B	CD27, IGHM, IGHD, IL4R
Dendritic cells	KLF4, CD1C
CD14 monocytes	CD14
plasmacytoid DC	IL3RA, TLR7
Plasma cells	PRDM1, IRF4

Table S5: Expert marker genes: blood datasets

Cell type	Marker genes
Memory B cells	CR2, CD27, MS4A1
Naive B cells	IGHM, IGHD, TCL1A
DC2	CLEC10A, FCER1A, CD1C
CD16+ NK cells	GNLY, FCGR3A, NKG7
Megakaryocytes/platelets	CMTM5, ITGA2B, PF4
Classical monocytes	S100A9, CD14, S100A12
Non-classical monocytes	FCGR3A, C1QA, CX3CR1
MAIT cells	KLRB1, SLC4A10, TRAV1-2
Regulatory T cells	CTLA4, IL2RA, FOXP3
Tcm/Naive cytotoxic T cells	CD8A, CCR7, SELL
Tcm/Naive helper T cells	CCR7, SELL, CD4
Tem/Trm cytotoxic T cells	GZMK, CD8A, CCL5
Tem/Temra cytotoxic T cells	CX3CR1, GZMB, GNLY
Tem/Effector helper T cells	KLRB1, AQP3, ITGB1

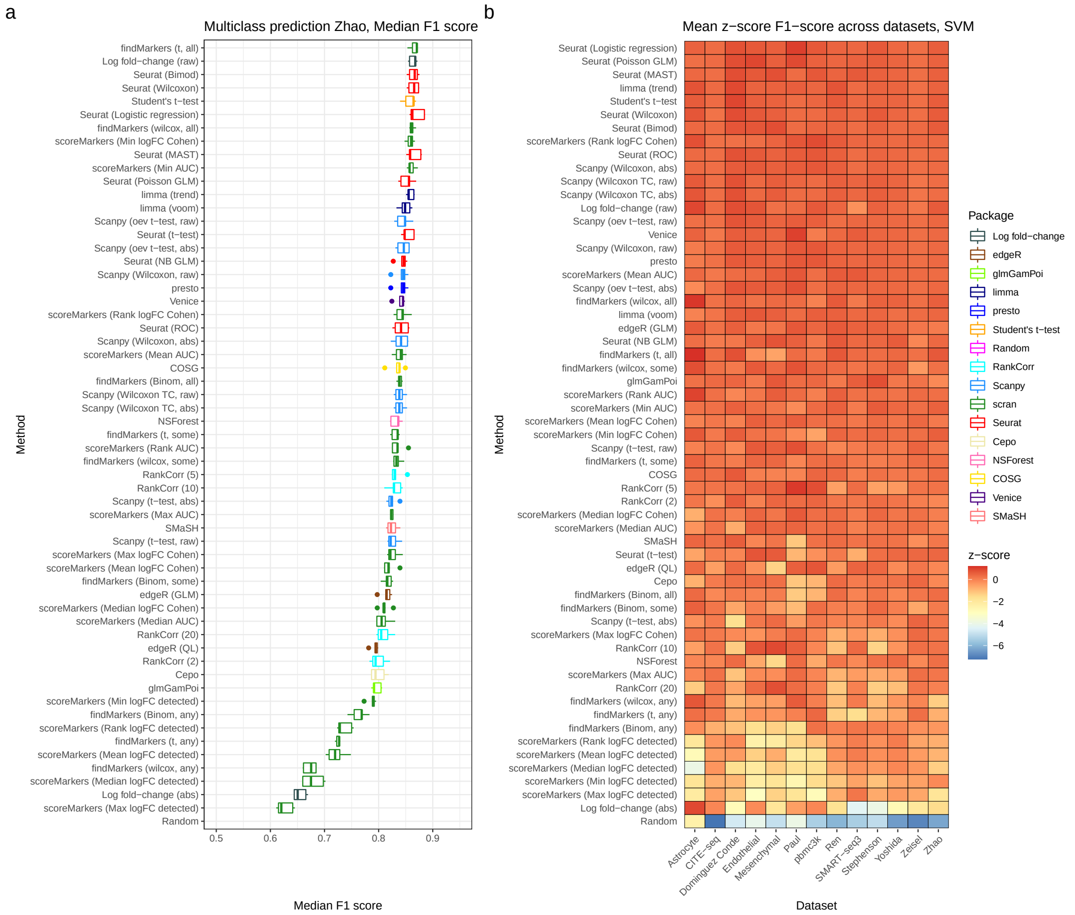

Figure S18: Predictive performance measured using the SVM classifier a) Median F1 score of the SVM classifier using genes selected by all marker gene selection methods in the Zhao dataset. Each point is the F1 score in one of the 5 folds. b) The z-score of the median F1 score of the SVM classifier (averaging across folds) in each dataset, for the classifier. Methods are ranked top to bottom by their mean z-score across datasets. Note that the Lawlor dataset is excluded from these results as the SVM classifier gave outlier results when run on it.

Table S6: Dataset cluster filtering

Dataset	Cluster
pbmc3k	B
Lawlor	Alpha
Zeisel	Oligodendrocytes
Paul	14Mo
Smart-seq3	Naive B
Endothelial	Endothelial - capillary
Astrocyte	Astro_HYPO
CITE-seq	2
Mesenchymal	Pericyte
Zhao	Naive B
Ren	Classical Monocytes
Stephenson	Classical Monocytes
Yoshida	Classical Monocytes
Dominguez Conde	Classical Monocytes

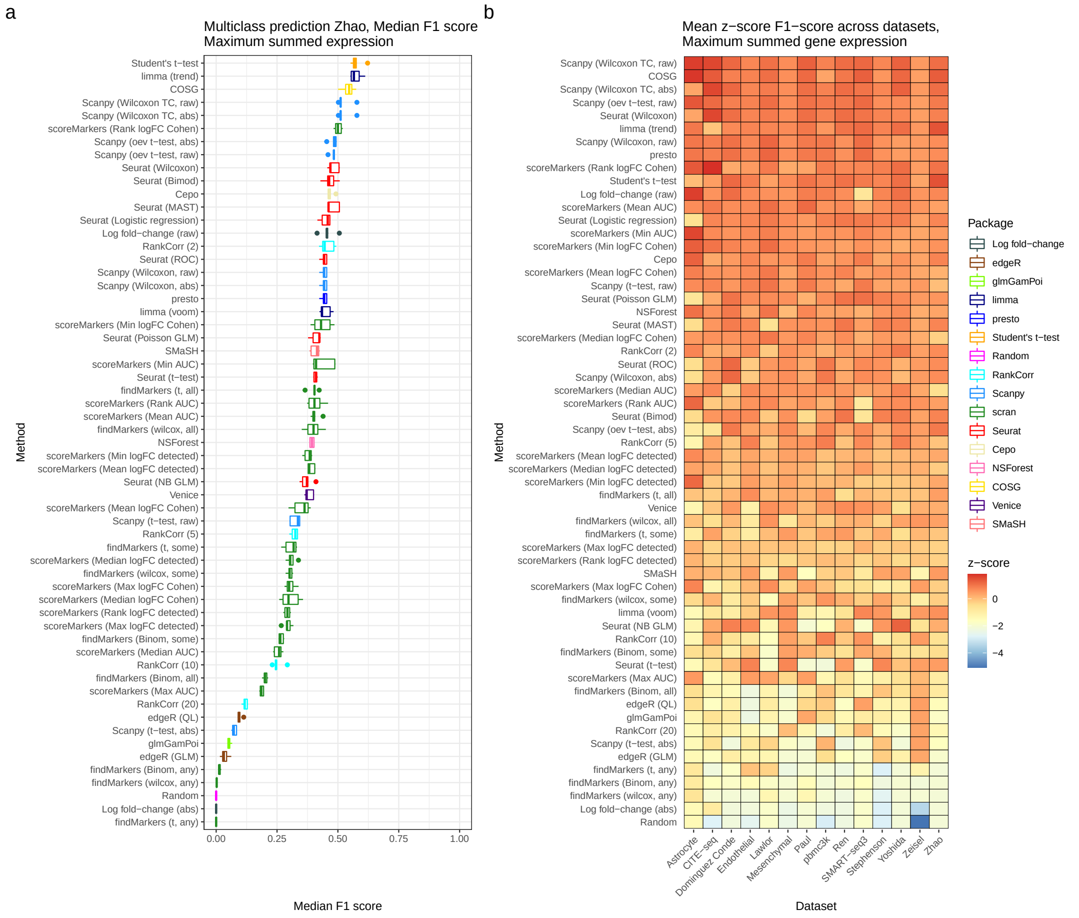

Figure S19: Predictive performance measured using the summed gene expression classifier a) Median F1 score of the maximum summed gene expression classifier using genes selected by all marker gene selection methods in the Zhao dataset. Each point is the F1 score in one of the 5 folds. b) The z-score of the median F1 score of the maximum summed gene classifier (averaging across folds) in each dataset. Methods are ranked top to bottom by their mean z-score across datasets.

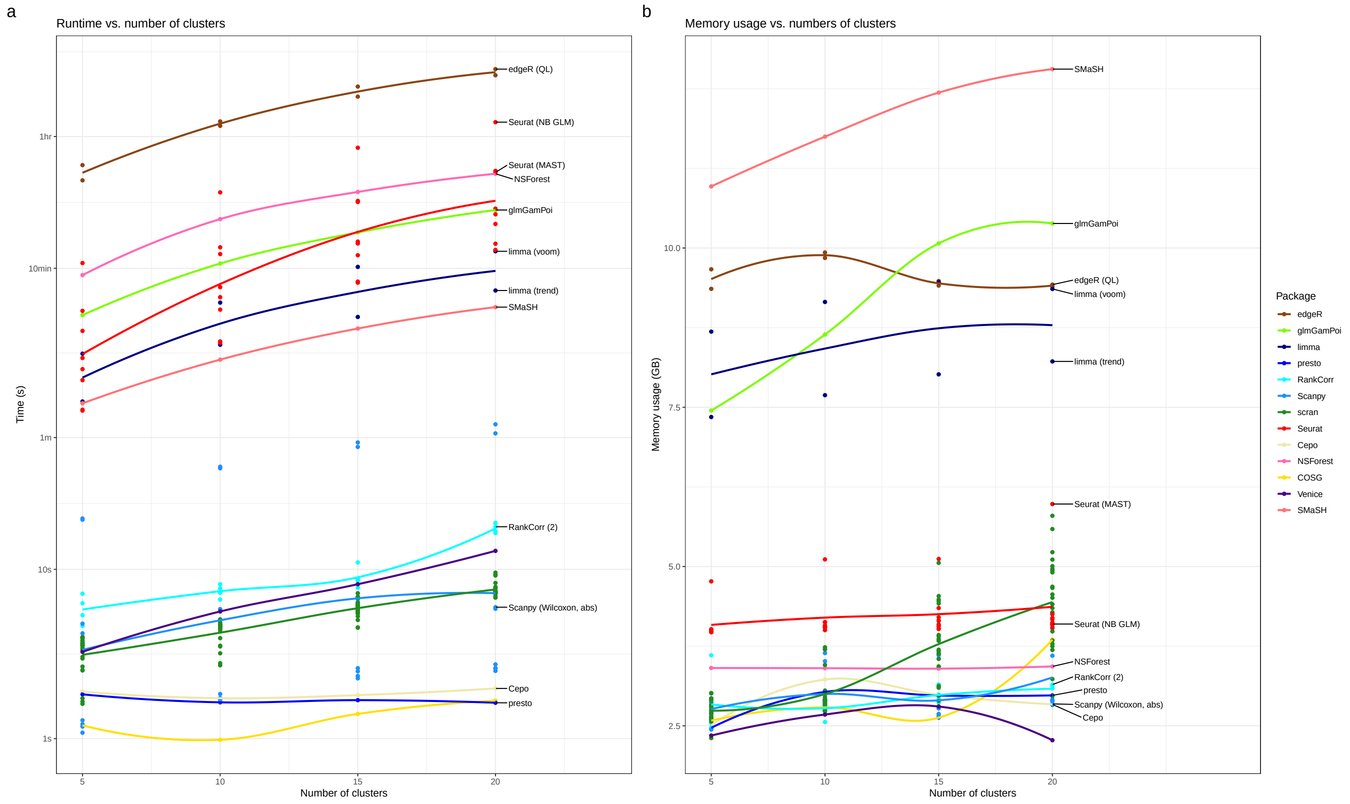

Figure S20: Time and memory usage of methods on simulated datasets with different numbers of clusters. All simulations had estimable parameters estimated from the pbmc3k dataset, DE factor location and scale parameters of 3 and 0.2, 20,000 cells and 2000 genes.

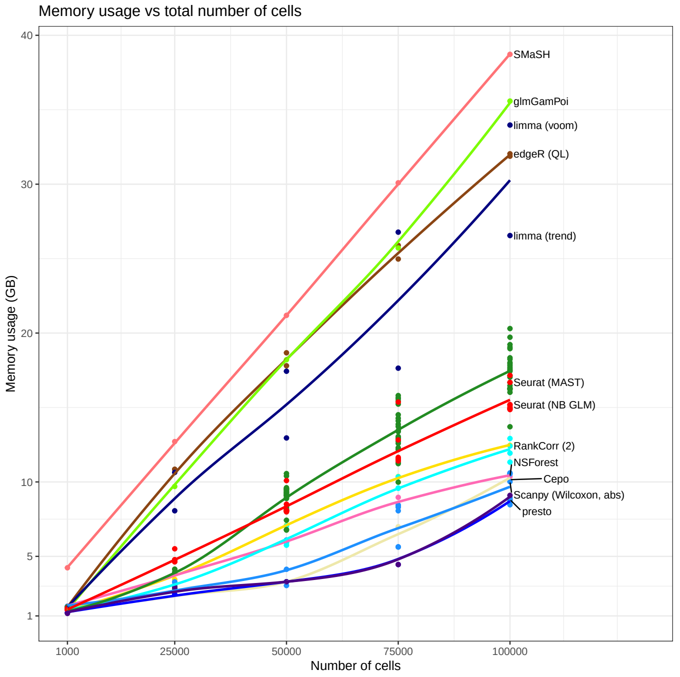

Figure S21: Memory usage of methods on simulated datasets with varying number of total cells All simulations parameters estimated from the pbmc3k dataset with DE factor location and scale parameters of 3 and 0.2 respectively. Datasets with 1,000, 25,000, 50,000, 75,000 and 100,000 total cells were simulated. All datasets had 5 clusters and 2000 genes.

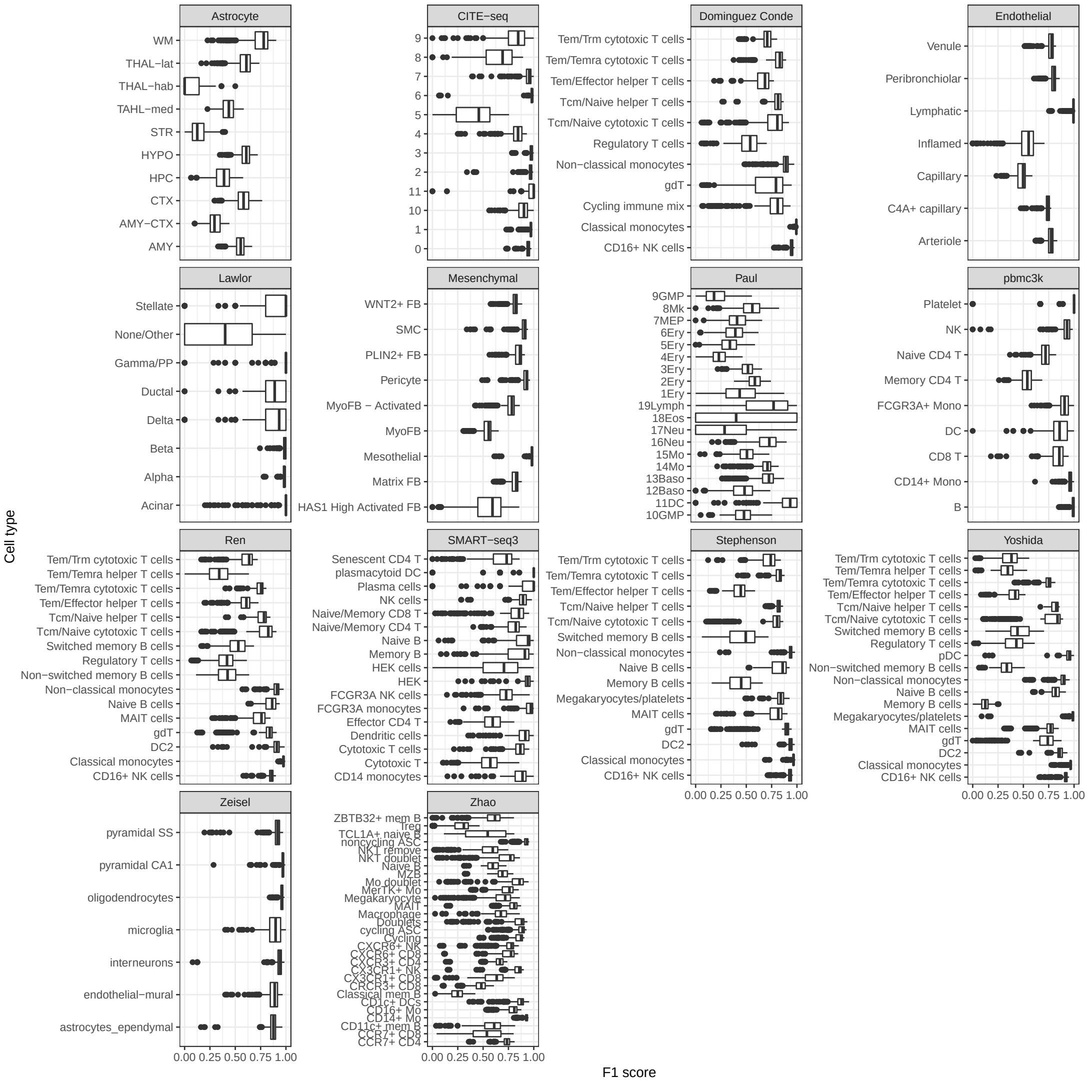

Figure S22: Predictive performance across cell-types for all methods Median F1 score of the KNN classifier for each method in each dataset visualised by cell-type and datasets. Across datasets cell-type has a large impact on predictive performance

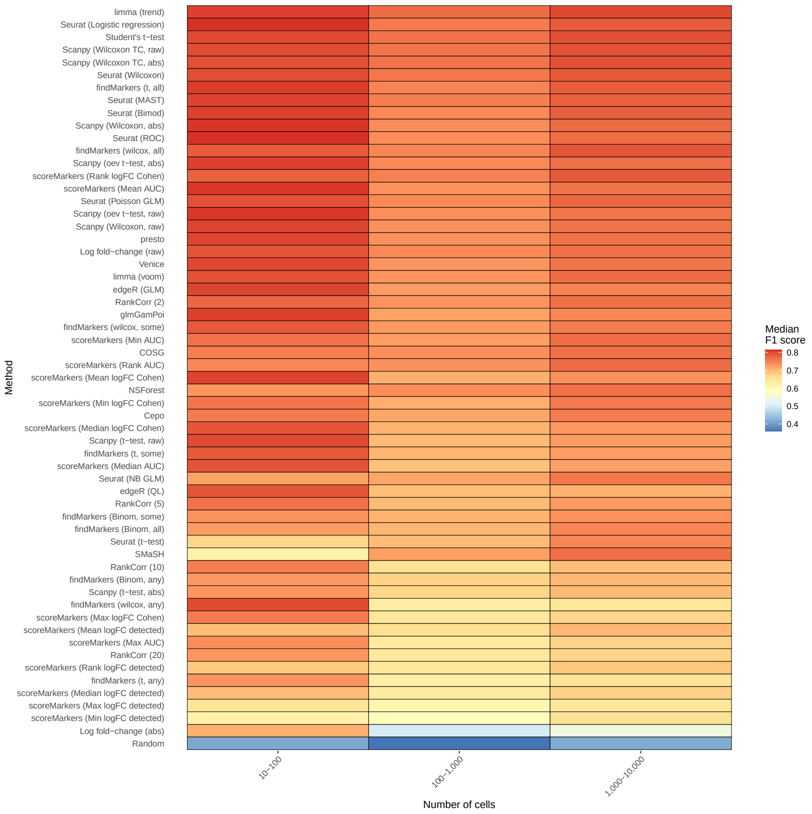

Figure S23: Predictive performance by the number of cells in the cluster Median F1 score of the KNN classifier for each method by the binned size of the cluster.

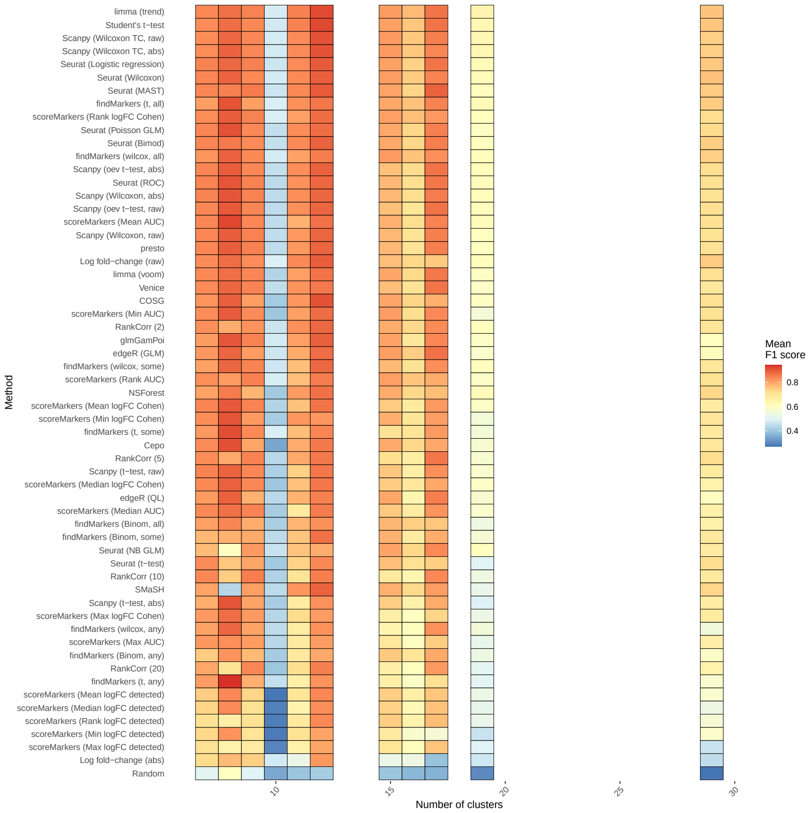

Figure S24: Predictive performance by the number of clusters in the dataset Median F1 score of the KNN classifier for each method by number of clusters in the dataset. Note that most dataset have a unique number of clusters.

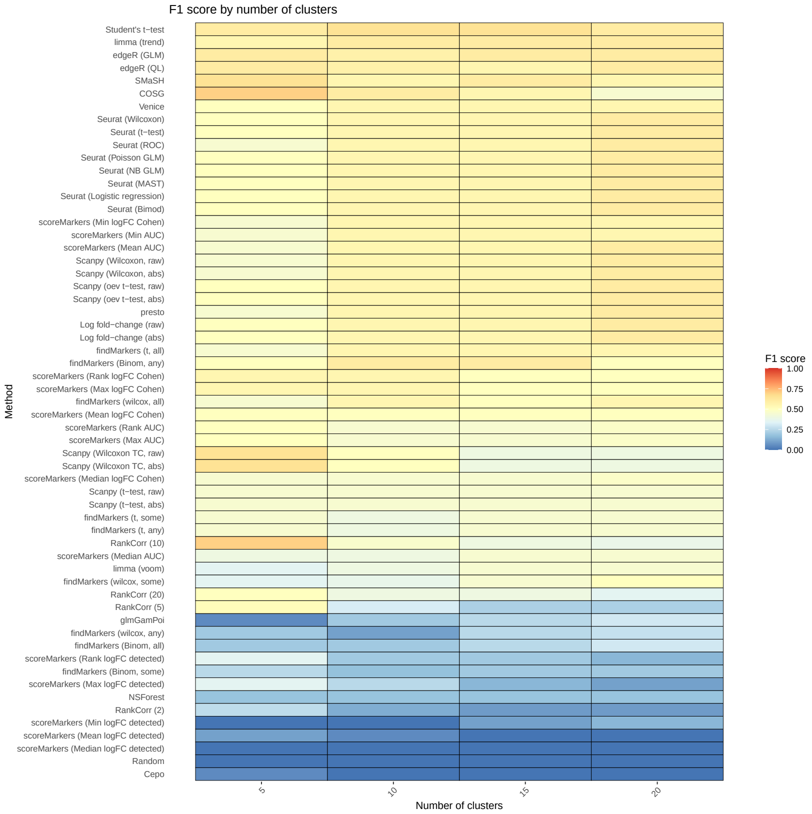

Figure S25: Recovery of simulated marker genes by the number of clusters in the simulated dataset Median F1 score for each method by the number of simulated clusters in the dataset.

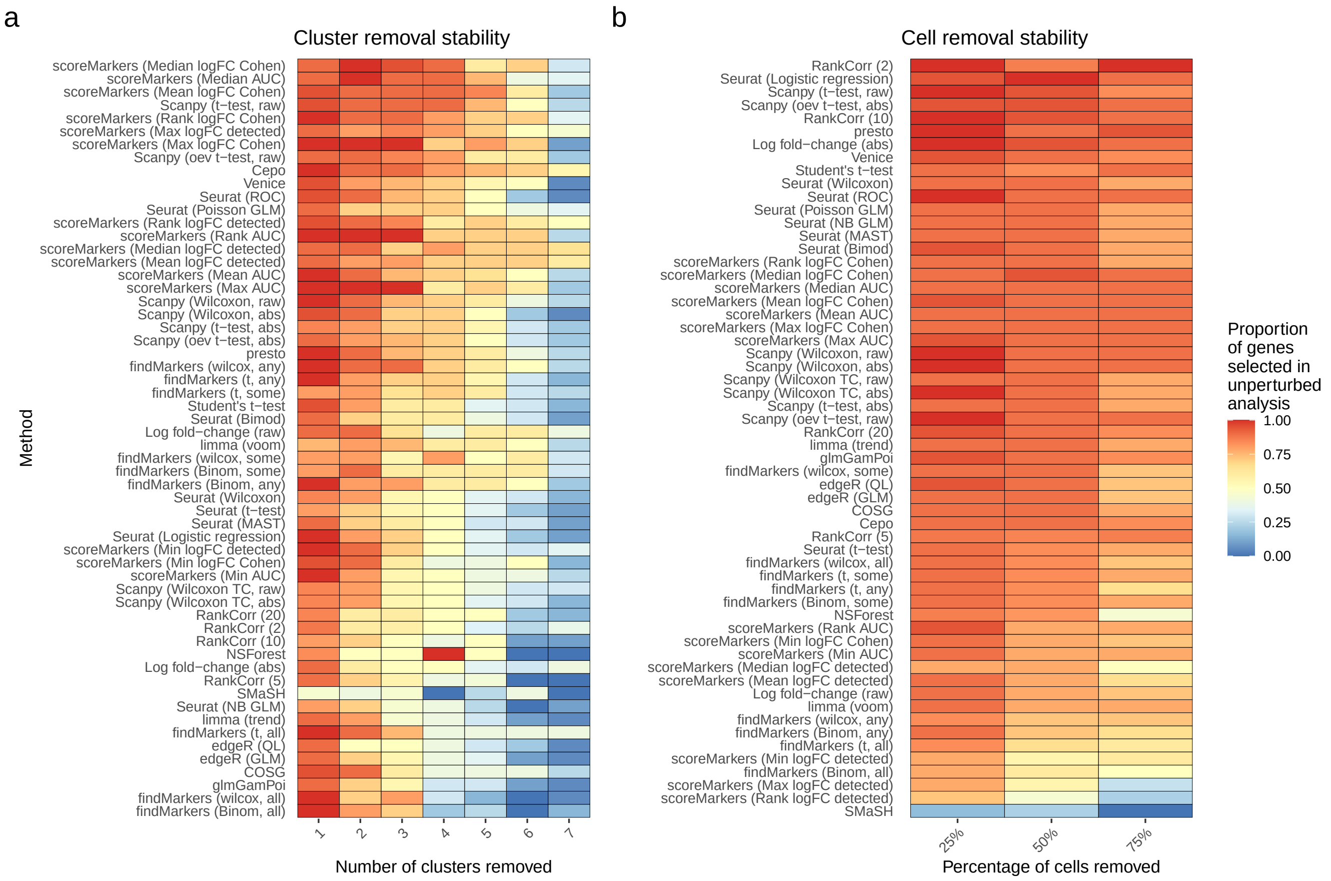

Figure S26: Performance of all methods on down-sampled data designed to assess method stability a) Simulations where increasing numbers of cluster are removed from the dataset. All methods show greatly reduced performance when the five or more clusters are removed b) Simulations where increasing proportions of cells are removed from the dataset. Method show generally worse performance as the number of cells decreases. In both cases the pbmc3k dataset is used as as the base dataset and the performance of methods is assessed as the the proportion of the at most ten top selected marker genes that are the same as for the analysis of the full dataset, averaged across the remaining clusters.

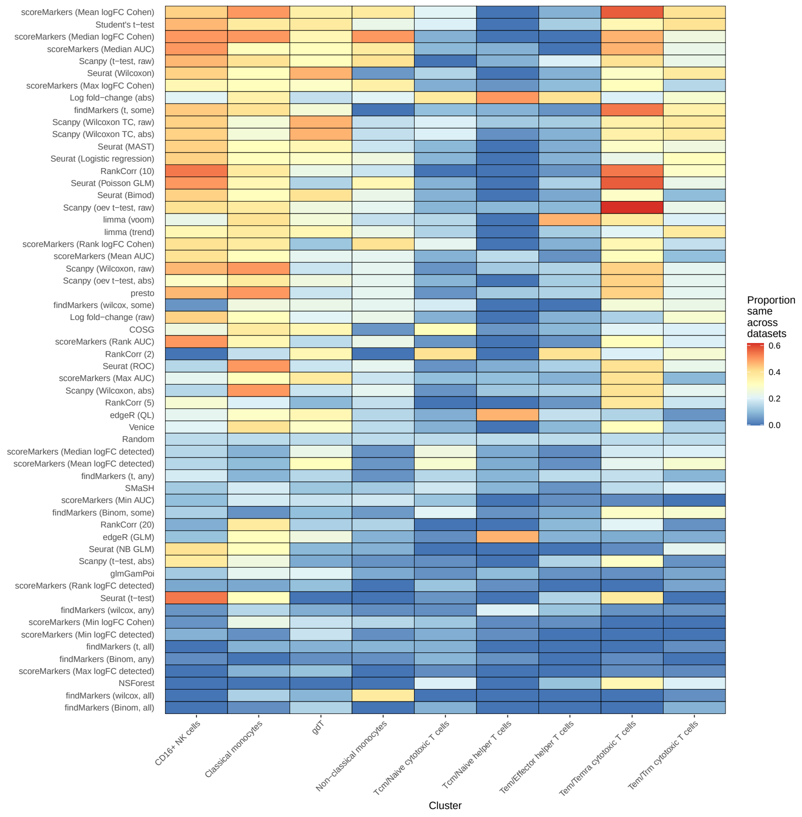

Figure S27: Concordance of methods across the 4 harmonised 'blood' datasets. Concordance is measured by the proportion of all the (at most) top 10 genes for each that were selected in all datasets. This analysis was performed on the clusters which appeared in all 4 datasets.

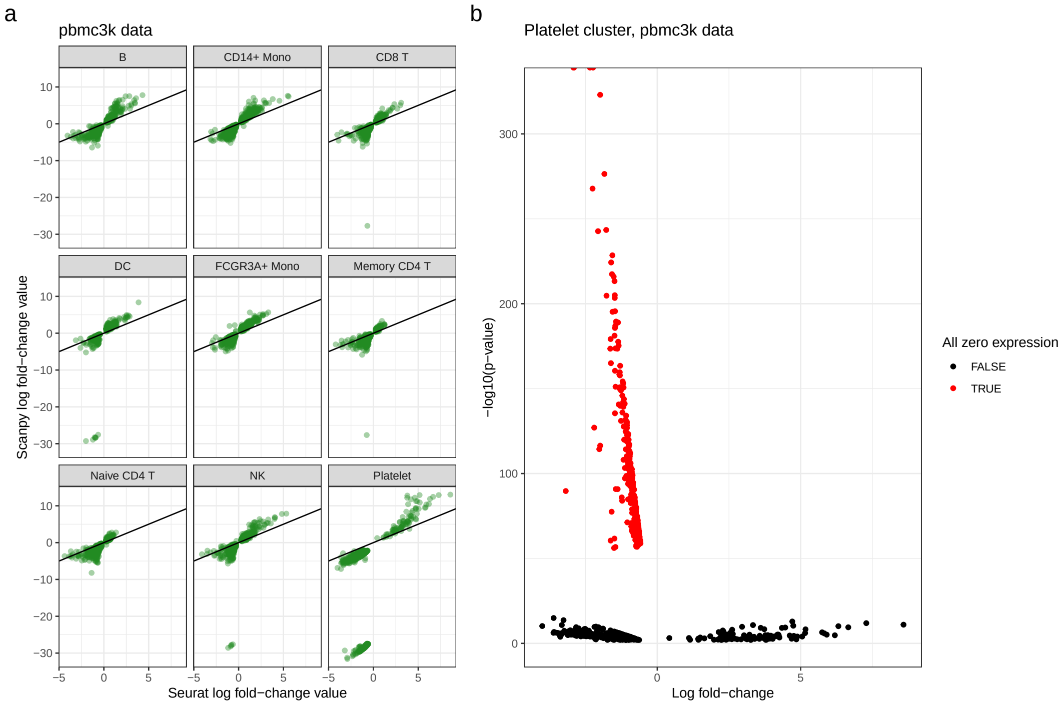

Figure S28: Consequences of exactly zero expression a) Scatter plots of Seurat and Scanpy calculated log fold-changes for each cluster in the pbmc3k dataset. The extreme values calculated by Scanpy for some clusters, such as the Platelet cluster, occur when there is exactly zero expression a particular cluster. b) Volcano plot of genes for the Seurat t-test method in the Platelet cluster pbmc3k. Genes are coloured by whether they show zero expression in the platelet cluster.
