# Supplementary material for: A comparison of marker gene selection methods for single-cell RNA sequencing data
Source: Genome Biol. 2024 Feb 26;25:56. doi: 10.1186/s13059-024-03183-0 (PMC10895860; doi:10.1186/s13059-024-03183-0)
Supplement: Supplementary file 2 — Additional file 2. Review history. [file 13059_2024_3183_MOESM2_ESM.docx]

Review History

**First round of review**

**Reviewer 1**

**Were you able to assess all statistics in the manuscript, including the appropriateness of statistical tests used?**

Yes, and I have assessed the statistics in my report.

**Comments to author:**

This is a well written and thorough analysis of marker selection methods in single cell RNA-seq. Its conclusions are perfectly reasonable. It spends at least some of its argument defending itself form the claim that marker selection is the same as differential expression. This is important since otherwise the paper is almost wholly duplicative of Soneson and Robinson in Nature Methods a few years ago. Despite the terminological argument, I don't see any difference between the two papers in terms of primary results, methods or conclusions. I think those conclusions are valid and warranted - and always due for repetition - but somewhat hard to justify being presented as novel research in Genome Biology. There are probably dozens or more of others that are relevant to the topic of marker selection, typically as a form of differential expression, and mostly drawing adjacent conclusions to Pullin and McCarthy.

An increase in data and modest alteration of the methods (yielding similar results) are all worthwhile contributions but the framing of this manuscript would need to be incredibly modest to be forthright. Or perhaps I am missing its essential contribution, which I would welcome having clarified. A simple update drawing confirmatory conclusions is also fine but then should really build on existing knowledge to clarify essential dependencies or arguments. I think this manuscript does not make a contribution of that class either.

**Reviewer 2**

**Were you able to assess all statistics in the manuscript, including the appropriateness of statistical tests used?**

Yes, and I have assessed the statistics in my report.

**Comments to author:**

The authors describe a computational benchmark of 56 approaches for marker gene selection using 10 real and 170 simulated datasets. The authors discuss different kinds of marker genes criteria such as genes in the same cluster from different samples, genes in different clusters coming from the same sample, or genes coming from a specific comparison of different conditions. Further, they highlight all-vs-rest, and pairwise comparison strategies. Overall, such a benchmark is relevant to identify the optimal tool for the dataset at hand. Here are some of my concerns and thoughts regarding the results presented in this paper. In particular, I would be very interested in finding out which cell types are easy to predict, and why. Are diverse datasets difficult for marker gene selection? do different methods agree at the cell-type level?

1. The size of the cluster - which methods are independent of the number of cells in the cluster? Are there methods that can be more accurate even from smaller clusters?
2. Which methods take into account both the number of cells expression those genes withing a cluster, and the level of expression? Do methods generally take the 'number of cells' expressing a marker gene into account? It would be good to know in case the experimental validation design.
3. Figure 1C shows some very interesting insights into the different methods tried here. There are some methods that show no overlap with other methods. E.g. the Lawlor data point at x=0 axis. This figure is too concise and does not clearly reveal which methods show high or low proportion of shared genes. Are these methods more or less consistent in their shared-genes irrespective of the dataset and the cluster. Again, here the number of cells in a cluster would be important to know.
4. How do authors deal with if the methods do not return 20 genes per cluster? Are the results obtained for each method obtained after multiple hypothesis correction?
5. Line 115 on page 5, how can down-regulated genes be used as marker genes for selection of a sub-population? Do the authors only focus on up-regulated genes or do they also attempt to include down-regulated genes in their comparisons?
6. Is it correct that the expert annotation is only done for 4 datasets out of the 10 real datasets? How were these 4 datasets selected?
7. Methods such as genesorter (Mahmoud Ibrahim, Rafael Kramann), AUCELL (Cole Trapnell), scGeneFit are not used for benchmarking. It is understandable that due to time and resource constraints not every method can be included, but would be nice to include some more ML based approaches.
8. Figure 3A and Table 2. Can the authors compare the performance of different methods for the same / similar cell-types across datasets. For example, fibroblasts or other cell types might occur in more than 1 dataset. For the same cell-types, do methods differ in their outcome based on the dataset used?
9. Line 389 on page 16 is a very relevant issue for computational methods and detection of marker genes for different resolutions. It would be nice to comment on which methods perform well specifically on high-resolution clustering.
10. Maybe I missed it, but the SMaSH method is not cited in the main text, 393 on page 16
11. Comet, scanpy's logistic regression, smash and venice methods could not be run. Its good to know that there were issues in making them run independently, however, it would be nice to figure out how some of those could still be included. E.g. by running them without snakemake pipeline? Or other solutions.
12. How are the self-implemented methods as described on page 20 different from generic methods available in scanpy and Seurat? I think these are just different ways to post-hoc select marker genes. Differences between these methods need to be described in more detail.
13. Figure 5c shows that the predictive performance of most features+method selection is pretty similar across datasets. In this case, MAST seems to be quite good. However, in figure 3 mast although being good, is ranked lower. What is reason for differences between these results.

**Authors Response**

**Point-by-point responses to the reviewers’ comments:**

Dear *Genome Biology* editors,

Thank you for the opportunity to revise and resubmit our paper “A comparison of marker gene selection methods for single-cell RNA sequencing data”.

We thank the editor and reviewers for their patience with our response, noting that Dr McCarthy has been on extended parental leave for much of the time since we received the reviewers’ comments.

The revised version of the manuscript includes a number of major changes in response to the reviewers’ insightful comments.

1. First, we have included a new section in the introduction of the paper that better articulates the differences between the manuscript and previous work benchmarking differential expression testing methods.
2. Second, we have added both the recent SMaSH and Venice methods to all comparisons.
3. Third, we have added four new real datasets. These datasets have associated expert-annotated marker genes, so that we now assess the performance of expert-annotated marker genes for a majority of datasets used in the paper.
4. Finally, we have added a new section to the paper which interrogates how dataset characteristics, such as cluster size and dataset diversity, affect the performance of marker gene selection methods.

In addition to these major changes we have also revised the text and added supplementary analyses in response to the reviewers comments.

Please see below for a full point-by-point response to the reviewers’ comments.

Yours faithfully,

Davis J. McCarthy and Jeffrey Pullin

**Reviewer #1:**

This is a well written and thorough analysis of marker selection methods in single cell RNA-seq. Its conclusions are perfectly reasonable. It spends at least some of its argument defending itself form the claim that marker selection is the same as differential expression. This is important since otherwise the paper is almost wholly duplicative of Soneson and Robinson in Nature Methods a few years ago. Despite the terminological argument, I don't see any difference between the two papers in terms of primary results, methods or conclusions. I think those conclusions are valid and warranted - and always due for repetition - but somewhat hard to justify being presented as novel research in Genome Biology. There are probably dozens or

more of others that are relevant to the topic of marker selection, typically as a form of differential expression, and mostly drawing adjacent conclusions to Pullin and McCarthy.

An increase in data and modest alteration of the methods (yielding similar results) are all worthwhile contributions but the framing of this manuscript would need to be incredibly modest to be forthright. Or perhaps I am missing its essential contribution, which I would welcome having clarified. A simple update drawing confirmatory conclusions is also fine but then should really build on existing knowledge to clarify essential dependencies or arguments. I think this manuscript does not make a contribution of that class either.

We thank the reviewer for their engagement with our work and critical assessment of the paper. However, they write that “perhaps I am missing its essential contribution, which I would welcome having clarified.” We would very much like the opportunity to clarify the novel contribution of our results, as we believe that Reviewer 1 has indeed missed the essential contributions of the paper.

Briefly, we would make a case for the novelty of our results on the following grounds:

- Foremost, marker gene selection and differential expression are not the same. Strictly, marker gene selection is a subset of differential expression, but marker gene selection used for the annotation of cells by cell type (our study) is conceptually and practically a very different task from the identification of all genes with statistically significant differences in expression between groups, with no consideration for whether or not detected DE genes are useful for distinguishing a given group from others (Soneson and Robinson, 2018; S&R2018). This is a somewhat subtle point that Reviewer 1 seems to have missed – in revisions we could easily take more space to explain this difference between marker gene selection and DE gene detection a la S&R2018.
- As marker gene selection and DE gene detection are different tasks, the results from S&R2018 are not portable to the challenge of marker gene selection. That specific fact motivated our study, because we recognised the necessity, urgency and value to the community of a rigorous benchmarking study *specifically* assessing the performance of methods for the selection of marker genes. Of course, many methods for marker gene selection are also more general DE detection methods, but that overlap in methods between our study and S&R2018 is irrelevant to the key fact that the marker gene selection *task* is distinct from the more general DE gene detection task.
- It is thus not fair to dismiss the differences between marker gene selection and DE gene detection as merely a “terminological” argument and it is unfair to suggest that our paper “is almost wholly duplicative” of S&R2018.
- Our results are novel and important because we designed and conducted a rigorous benchmarking of marker gene selection methods for the specific and distinct task of the selection of marker genes from scRNA-seq data. We took inspiration from some parts of the excellent and important S&R2018 study, but in no way did we duplicate that work – they assessed performance of DE methods on a related but different task. We may stand on the shoulders of giants, but with this work we forge our own path into uncharted terrain.

Beyond those comments, we would add:

- our study has a substantially different simulation setup to S&R2018, one that we designed very specifically for the distinct context and challenges of marker gene selection;
- we benchmark a variety of more modern ML methods;
- we have many new and specific analyses for assessing marker gene selection methods, using datasets particularly suited to the task (for example the use of many datasets with expert-annotated marker genes that provide a valuable point of comparison for computational methods), that are (beyond perhaps superficial resemblance) not at all like the analyses presented in S&R2018;
- we make very different recommendations about preferred methods for marker gene selection compared with S&R2018’s recommendations for the more general DE detection task (for example, we do not recommend edgeR due to different considerations, i.e less focus on FDR more on speed);
- our results assessing the performance of scanpy and Seurat options in widespread use today are both novel and highly important;
- we undertook very detailed, unique case studies scrutinising the inconsistencies found between scanpy/Seurat, which are both entirely novel and highly important and were sadly not mentioned by the reviewers.

Finally, we note that Reviewer 2 did not share Reviewer 1’s concern about the novelty of our results. Indeed, the community response to the pre-print of our paper does not at all support Reviewer 1’s contention that our results lack novelty. Our pre-print attracted enormous interest and discussion on Twitter on its release, and the pre-print has been tweeted by 242 people, has had >12,000 abstract views, >5,000 PDF downloads and receives an Altmetric score of 131, placing it in the top 5% of all research outputs scored by Altmetric (<https://www.biorxiv.org/content/10.1101/2022.05.09.490241v2.article-metrics).>

For all of these reasons, we must politely contest Reviewer 1’s assessment that our results lack sufficient novelty for publication in Genome Biology. The reviewer did, I argue, miss our work’s “essential contribution”. Happily, the community has recognised the importance and value of our work, and I remain convinced that this paper would be worthy – judged on novelty, importance, and scientific quality – of publication in Genome Biology.

To address the concerns raised by the reviewer, which indicate that we did not adequately communicate the value and novel contributions of our paper, we have substantially rewritten and extended the middle paragraphs of the introduction, adding more explanation of the differences in the tasks of marker gene selection and general DE gene and better, more detailed, and clearer justification of the need for our study specifically benchmarking marker gene selection methods. The revised paragraphs of the introduction are copied below:

Today, many marker gene selection methods are available. These methods range from

relatively simple differential expression based methods to those based on modern machine learning ideas. The most commonly used methods are implemented as part of the Seurat \citep{hao2021} and Scanpy \citep{wolf2018} analysis frameworks, which both implement a variety of method options. More recently, bespoke tools have also been developed, as marker gene selection continues to be an active area of method development \citep{aevermann2021, nelson2022, dai2022}. However, unlike other areas of scRNA-seq data analysis such as normalisation, differential expression analysis or trajectory inference \citep{cole2019, soneson2018, saelens2019}, no impartial and comprehensive comparison of the different marker gene selection methods is available. The lack of such a comparison leaves analysts unaware of the relative performance of different methods. Furthermore, because the most commonly used methods are implemented in larger analysis frameworks, no publications exist that describe in detail or justify their marker gene selection methods. Therefore, their widely used methods are not supported by publicly scrutinised evidence of their effectiveness.

The concept of the marker gene is closely related to that of the differentially expressed (DE) gene, but the two concepts are not synonymous. Strictly, marker gene selection is a subset of the identification of DE genes, but effective and useful marker genes have specific characteristics that are not shared by all DE genes. Broadly, we define a marker gene as a gene that can be used to distinguish between sub-populations of cells. Good marker genes typically exhibit a large difference in expression between cell types and, canonically, are strongly up-regulated in a cell type of interest, exhibiting high expression in that cell type and no or low expression in other cell types. Thus, a marker gene is a narrower, more specific concept than that of a DE gene, which is context dependent. In the analysis of scRNA-seq data, the term DE gene does not have a unique meaning as it refers generally to a gene that shows a statistically significant difference in mean expression in a specific comparison. DE genes can therefore refer to genes found in a comparison between cells in different clusters in the same sample \citep{soneson2018, finak2015} or in the same cluster between different samples \citep{crowell2020a, squair2021, zimmerman2021}, or to a comparison between arbitrary groups of cells \citep{megill2021}. When DE methods are used to identify marker genes a decision has to be made about how to map the idea (and desirable characteristics) of marker genes to a concrete between-groups comparison. Indeed, different methods use different concrete comparisons: Seurat and Scanpy use a `one-vs-rest' cluster comparison strategy, while scran employs a `pairwise' approach (see Methods for details). Significantly, both of these strategies are different to those compared in previous benchmarks of single-sample DE methods \citep{soneson2018, wang2019a} that focused on comparing two specific cell sub-populations. For example, the `one-vs-rest' strategy creates a situation with highly imbalanced sample sizes and increased biological heterogeneity in the pooled `other' group, both of which we would expect to pose significant challenges to DE methods. As such, marker gene selection used for distinguishing between sub-populations of cells is conceptually and practically a distinctly different task from the more general challenge of identification of all genes with statistically significant differences in expression in a given comparison context, with no consideration for whether or not detected DE genes are useful for distinguishing a given group of cells from others.

The realisation that marker gene selection and DE gene detection are distinctly different tasks motivated the current study. We identified that the conclusions from previous, often excellent, benchmarking studies of methods to detect DE genes from scRNA-seq data \citep{soneson2018,wang2019a} are not directly portable to the task of marker gene selection. Thus, dedicated benchmarking of marker gene selection methods is necessary given the centrality of marker genes in scRNA-seq analyses and the fact previous DE gene identification benchmarking efforts do not provide actionable advice for marker gene selection. Of course, many methods for marker gene selection are also more general DE gene detection methods, but while many methods can be used for both DE gene detection and marker gene selection we observe that their performance and suitability can differ greatly on the two tasks. Here, we designed and conducted a rigorous benchmarking of methods for the specific and distinct task of the selection of marker genes from scRNA-seq data, taking inspiration and some important lessons from previous DE gene detection benchmarking studies \citep{soneson2018, wang2019a}.

In this paper, we compared 59 methods for selecting marker genes in scRNA-seq data. The methods were benchmarked using 14 scRNA-seq datasets, encompassing a range of protocols and biological samples, as well as over 170 additional simulated datasets. We benchmark a variety of methods (applying both traditional statistical and modern machine learning approaches) that have been designed specifically for the task of marker gene selection and are not applicable to more general DE gene detection. This study has a substantially different simulation setup compared to DE gene benchmarking studies, one that we designed specifically for the distinct context and challenges of marker gene selection. More than half of the datasets used in this study are bolstered by expert identification of marker genes to which we can compare the marker genes selected by computational methods. This work uses new and specific analyses for assessing marker gene selection methods, compared with prior work benchmarking general DE gene detection methods. Specifically, methods were compared on their ability to recover simulated and expert-annotated marker genes, the predictive performance and characteristics of the gene sets they select, their memory usage and speed, and their implementation quality (\autoref{fig:conceptual-figure}b). To scrutinise the methodologies of the most commonly used methods, we created case studies based on the specific observations made during benchmarking. These case studies highlight several notable issues with, and inconsistencies between, the methods implemented in the Scanpy and Seurat packages. Importantly, we make different recommendations about preferred methods for marker gene selection compared with recommendations for the more general DE gene detection task from prior DE gene detection benchmarking work, providing immediate, actionable information for researchers relying on marker gene selection in their scRNA-seq data analyses.

**Reviewer #2**:

The authors describe a computational benchmark of 56 approaches for marker gene selection using 10 real and 170 simulated datasets. The authors discuss different kinds of marker genes criteria such as genes in the same cluster from different samples, genes in different clusters coming from the same sample, or genes coming from a specific comparison of different conditions. Further, they highlight all-vs-rest, and pairwise comparison strategies. Overall, such a benchmark is relevant to identify the optimal tool for the dataset at hand. Here are some of my concerns and thoughts regarding the results presented in this paper.

In particular, I would be very interested in finding out which cell types are easy to predict, and why.

We thank the reviewer for this interesting question. We have added analysis of the predictive performance of methods at the level of clusters within datasets. This analysis highlights that there is substantial variability in how easy different cell-types are to predict. We observed that cell-types that are very phenotypically distinct from other cells in a dataset, such as the platelet cluster in the pbmc3k dataset were more easy to predict. The relevant section of the revised manuscript is:

First, we visualised the predictive-performance of methods broken down by cell type.(\autoref{fig:cell-type-pred-perf}). The effect of cell type was larger than differences between methods in many datasets. Methods performed better on cell types that were phenotypically very distinct from other cells in the dataset, such as the platelet or B cell clusters in the pbmc3k dataset.

Are diverse datasets difficult for marker gene selection?

We thank the reviewer for raising this interesting question. We explored this question by assessing the effect of the number of clusters, a rough proxy for diversity, on method performance. In real datasets methods generally performed slightly worse as the number of clusters increased. In simulated datasets, however, increasing the number of clusters had no impact on method performance. Overall, the results suggest that diversity does not have a large impact on method performance.

As the number of clusters in the datasets increase methods generally performed worse, though the effect was small. This trend suggests that methods perform worse when datasets are more diverse. However, when methods were run on simulated datasets with different numbers of clusters, no strong effect of cluster size was observed (\autoref{fig:n-clusters-sim}). Taken together, these results suggest that diversity, at least as measured by the number of clusters, does not have a large impact on method performance. No methods displayed particularly strong performance in the high resolution scenarios where there are many clusters.

Do different methods agree at the cell-type level?

We thank the reviewer for raising this interesting question. Please see our response to the same point later in the review.

1. The size of the cluster - which methods are independent of the number of cells in the cluster? Are there methods that can be more accurate even from smaller clusters?

We have added an assessment of the predictive performance of methods by the (discretized) size of the cluster. This analysis shows that methods are generally more effective when clusters are smaller. However, the analysis also revealed that the SMaSH method performs poorly on clusters with fewer than 100 cells. The relevant text in the revised manuscript is:

Next, we visualised the predictive performance of all methods by the number of cells in the cluster (\autoref{fig:n-cells-pred-perf}) ... Generally, methods performed better on smaller clusters, possibly because they represent better defined biological cell types. However, unlike most methods, the SMaSH method performed much worse when the cluster size was less than 100.

2. Which methods take into account both the number of cells expression those genes withing a cluster, and the level of expression? Do methods generally take the 'number of cells' expressing a marker gene into account? It would be good to know in case the experimental validation design.

We have added the following text to the manuscript to clarify these important questions:

Most methods compare the overall expression difference between clusters for each cell. This comparison takes into account both the size of any expression difference and the number of cells in which an expression difference exists. The scran-binomial method, however, discretizes expression values as either expressed or not expressed, and therefore only takes the number of cells expressing a gene into account.

3. Figure 1C shows some very interesting insights into the different methods tried here. There are some methods that show no overlap with other methods. E.g. the Lawlor data point at x=0 axis. This figure is too concise and does not clearly reveal which methods show high or low proportion of shared genes.

We thank the reviewer for their comment that Figure 1c shows “very interesting insights”. However, we note that the figure only shows the overlap between the default methods in Seurat and Scanpy for each cluster in all real datasets. Indeed, Figure 2a, is intended to give an overall summary of which methods select the same genes across datasets. We have also added new supplementary analysis for Figure 2a in these revisions, described below.

Are these methods more or less consistent in their shared-genes irrespective of the dataset and the cluster.

To extend the analysis presented in Figure 2a we added a supplementary figure reproducing the dendrogram using output of methods run on a specific dataset, for 4 datasets. These results show that the pattern of concordance is consistent both between datasets and compared to the concordance of methods across all datasets. The relevant text in the revise manuscript is:

Calculating the dendrogram on the results for specific datasets showed qualitatively similar conclusions (\autoref{fig:per-dataset-dendros}).

Again, here the number of cells in a cluster would be important to know.

We additionally performed and included an analysis of the relationship between cluster size and overall method concordance, which showed no strong relationship. The relevant text in the revised manuscript is:

Also, the proportion of genes shared between methods did not show a relationship to the number of cells in a given cluster (\autoref{fig:intersect-n-cells}).

4.	How do authors deal with if the methods do not return 20 genes per cluster?

If a method does not return 20 genes per cluster we simply use all the genes the method returns. We have added the following sentence early in the article to clarify this point:

If a method returns less than 20 marker genes per cluster, we use all the genes the method returns.

It is difficult to compare methods that return a small number of genes (NSForest, RankCorr) to methods that rank all genes, and leaves the selection of the set of marker genes to the users (Seurat, scanpy etc). Our benchmarking attempts to compare the two types of methods fairly, using the following approaches.

*Use of precision*

Precision, and F1 score calculated using precision, is included as a metric in the assessment of simulated method’s performance. Methods that select a small number of marker genes should have a small number of false negatives, giving them high precision. Indeed, methods that select a small number of marker genes do have high precision. Therefore this metric will give an advantage to methods that select a small number of genes.

*Small set of marker genes used in predictive comparison*

In the predictive-performance comparison section, only the (at most) top 5 marker genes are used to compare methods. Therefore, methods that return only 2 or 3 marker genes are not placed at a large disadvantage compared to methods that rank all genes.

*Small expert marker genes sets*

For the lawlor and harmonized blood datasets, the expert marker gene sets contain one and three genes per cluster respectively. For these datasets we only select the same number of genes from each method as there are marker genes.

Are the results obtained for each method obtained after multiple hypothesis correction? Yes, we have added the following text to clarify this:

Among DE-based methods a variety of multiple-hypothesis correction methods are used (see Methods for details).

5. Line 115 on page 5, how can down-regulated genes be used as marker genes for selection of a sub-population? Do the authors only focus on up-regulated genes or do they also attempt to include down-regulated genes in their comparisons?

We thank the reviewer for raising this important point. As we describe in the text, while some methods, such as Scanpy, only select up-regulated genes, many methods, including Seurat, can select marker genes that are down-regulated in the particular cluster of interest. A down-regulated marker gene could be useful if the absence of expression for a gene characterizes a particular cell type. Such a situation, however, is rare as cell types in transcriptomic data are normally defined and understood based on the presence of the expression of one or more genes.

In our comparisons, we focus on up-regulated genes, but also benchmark methods based on all genes they return. Specifically, in our simulated data comparisons, we only simulate genes that are up-regulated, and hence filter the method’s output to only up-regulated genes. Briefly, we perform this step as simulating down-regulated marker genes is technically difficult using existing scRNA-seq simulation approaches, which parameterise their models by the presence of expression, and because we could not identify sufficient expert-curated down-regulated marker genes to ensure the simulations were realistic. However, in both the expert-annotated-based and simulation-based comparisons of methods we do not restrict the type of marker genes each method selects.

6. Is it correct that the expert annotation is only done for 4 datasets out of the 10 real datasets? How were these 4 datasets selected?

In the version of the paper sent for review only 4 out of 10 datasets had expert-annotated (i.e. expert selected) marker genes that we could source. While almost all papers have some expert-driven annotation of clustering results, many papers do not record the details of marker genes used in the annotation process. We were therefore only able to source ‘expert’ marker genes for 4 of the 10 datasets we used. We have now added an additional 4 datasets, which have expert-

curated marker genes for most of their clusters. Therefore, we now assess the performance of methods using expert-curated marker genes on a majority (8/14) of datasets used for comparison in the paper.

7.	Methods such as genesorter (Mahmoud Ibrahim, Rafael Kramann), AUCELL (Cole Trapnell), scGeneFit are not used for benchmarking. It is understandable that due to time and resource constraints not every method can be included, but would be nice to include some more ML based approaches.

Thank you for drawing our attention to those approaches. Please see our comments below on each approach

genesorter

Genesorter only returns a set of ‘marker genes’ that distinguish all cell-types, not a set of genes for each cell-type. It therefore does not fit into the paper’s scope of benchmarking methods that select cell-type specific marker genes.

AUCELL

The description of AUCELL says that it focuses on analyzing the “state of gene-sets”, not marker genes. For this reason we have chosen not to include it in the paper.

scGeneFit

The paper describing scGeneFit clearly identifies it as a method for selecting marker genes. However, closer examination of the method reveals it, like genesorter, is designed to select one set of genes for the whole dataset that best distinguish the observed clusters. The scGeneFit manuscript particularly highlights the utility of this type of gene set in designing gene panels for spatial transcriptomics experiments, where only a small number of genes can be assayed. Our manuscript benchmarks methods that select cell-type specific marker genes, so scGeneFit is out of scope.

To avoid confusion, we have added a short section to methods describing methods, including both genesorter, AUCELL and scGeneFit, that are related to but outside the scope of the paper. The relevant manuscript text is:

A small number of methods perform analysis tasks similar to, but not the same as, the task of selecting cell-type specific marker genes. These methods are therefore out of scope for the benchmarking performed in this paper.

First, AUCELL \citep{aibar2017} analyses geneset activity, not cell-type specific marker genes, in scRNA-seq data.

Second, a number of methods, including genesorter \citep{ibrahim2019}, scGeneFit \citep{dumitrascu2021}, SCMarker \citep{wang2019}, and scTIM \citep{feng2020}, aim to select a single subset of so-called marker genes that best preserve information about the overall cluster structure of the dataset. This aim is generally motivated by the need to reduce the dimensionality of the dataset or to create gene panels for spatial transcriptomics experiments where only a limited number of genes can be assayed. These sets of genes are not per-cluster and do not distinguish particular cell types, so these methods are out of scope for the benchmarking of cell-type specific marker gene selection methods presented in this paper.

8. Figure 3A and Table 2. Can the authors compare the performance of different methods for the same / similar cell-types across datasets. For example, fibroblasts or other cell types might occur in more than 1 dataset. For the same cell-types, do methods differ in their outcome based on the dataset used?

We thank the reviewer for raising this interesting question. We note that it is often difficult to compare cell-types across datasets, as different clustering and annotation approaches may have been applied. We therefore use four blood datasets that have harmonized cell-types (Xu 2023). This analysis shows that methods are only moderately consistent across datasets for the same cell type, with methods being especially inconsistent for particular cell-types. The relevant manuscript text is:

Finally, we assessed the concordance of methods when run on the same cell type in multiple datasets. To perform this analysis we used four Human Cell Atlas blood datasets datasets harmonised using recent automatic methods \citep{xu2023}. Methods were run, returning the (at most) top 10 genes, on all clusters that appeared in all 4 datasets. To summarise concordance, we calculated the proportion of all selected genes that were selected in all 4 datasets (\autoref{fig:blood-dataset-concordance}). Overall, methods were moderately concordant across datasets, with substantial variability between methods and clusters. The most concordant methods were the Student's t-test method and the \texttt{scoreMarkers()} methods that used Cohen's D. The three T cell subtypes showed substantially less concordance across methods. As methods for harmonisation of cell types are still emerging, these results may be sensitive to the details of the harmonisation strategy used.

9. Line 389 on page 16 is a very relevant issue for computational methods and detection of marker genes for different resolutions. It would be nice to comment on which methods perform well specifically on high-resolution clustering.

We agree that high-resolution clustering is an interesting scenario to investigate methods performance. We have added a comment that in our real data and simulated comparisons no methods perform particularly well, or poorly, iin this scenario.

10. Maybe I missed it, but the SMaSH method is not cited in the main text, 393 on page 16

Thank you for drawing our attention to this issue. We have now removed the reference to SMaSH in that section of the text due to its inclusion in the benchmarking.

11. Comet, scanpy's logistic regression, smash and venice methods could not be run. Its good to know that there were issues in making them run independently, however, it would be nice to figure out how some of those could still be included. E.g. by running them without snakemake pipeline? Or other solutions.

Thank you for raising this issue. We have now updated the manuscript to include both the smash and Venice methods. We have not included the COMET and Scanpy’s logistic regression methods for the reasons below.

*COMET*

Unlike all other methods benchmarked in the manuscript COMET does not run solely in a programming language session: it outputs intermediate files containing information about the marker genes. This feature makes it very difficult to run in the highly parallelized snakemake environment. In addition, previous (non-independent) benchmarking in the paper describing rankcorr, has shown that COMET is too computationally intensive to run reliably.

*Scanpy’s logistic regression method.*

This method does not return what its documentation says it does, and is not tested in Scanpy’s test suite. We therefore feel that it is not appropriate for users to run it. In addition, Seurat’s logistic regression method allows the performance of logistic regression to still be assessed in the benchmarking. Therefore we do not think it is appropriate to add Scanpy’s logistic regression method.

12. How are the self-implemented methods as described on page 20 different from generic methods available in scanpy and Seurat? I think these are just different ways to post-hoc select marker genes. Differences between these methods need to be described in more detail.

Thank you for this comment. We have substantially expanded the section that describes the self-implemented methods, highlighting the difference compared to the methods implemented in scanpy and Seurat. The updated section reads:

In addition to the existing methods we implemented several methods:

- Random: A random sample of genes are selected as marker genes. This method provides a 'negative control'; it illustrates the worst possible perfor mance in a given scenario.
- Log fold-change sorted by absolute value.

First, for each cluster a one-vs-rest log fold-change value is calculated (using Seurat's formula) for each gene. Second, genes are ranked by the absolute value of the log fold-change. Finally, the top $n$ (i.e. with $n = 20$) genes in the ranking are then selected as the marker genes for that cluster. This method is implemented to assess whether ranking by effect size (log fold-change) rather than p-value, as for all DE methods, is sufficient to detect marker genes. Ranking by absolute values ensure that both up- and down-regulated genes can be selected as marker genes.

- Log fold-change sorted by absolute value.

As above, expect that the genes are ranked by the absolute values of the log-fold change. Ranking by the absolute values means that both up-regulated and down-regulated genes can be selected as marker genes.

- Student's t-test: A one-vs-rest two sample Student's t-test is performed between the cluster of interest and all other clusters for each gene. The top $n$ marker genes ranked by the test's p-value are selected as marker genes. This method is implemented to assess whether Student's t-test may be more suitable for selecting marker genes compared to Welch's t-test. The t-tests implemented in scran, Scanpy and Seurat are Welch's t-test. Briefly, Student's t-test assumes that the two samples have the same variance, while Welch's t-test allows the variances to be unequal.

For the log fold-change methods we implement Seurat's formula for the log fold-change using the log-normalised counts as input.

13.	Figure 5c shows that the predictive performance of most features+method selection is pretty similar across datasets. In this case, MAST seems to be quite good. However, in figure 3 mast although being good, is ranked lower. What is reason for differences between these results.

Thank you for your question regarding the difference between the rankings of MAST in Figure 3 and 5. Figure 3 visualizes the ranking of methods based on their performance on simulated datasets. Briefly, we simulate datasets with known marker genes and then assess whether the methods can recover the known marker genes. In these comparisons MAST performs well, but is not in the top few methods. Figure 5, however, visualizes the ranking of methods based on their predictive performance. Methods are run to select 5 marker genes for each cluster in the real data sets. The expression data for only those genes is then used as input to a classifier for cluster status. If a method selects marker genes that discriminate between clusters well, the classifier should also perform well. In these comparisons MAST is the second best method. There are many reasons why MAST’s ranking in the two comparisons may be different. Ultimately, Figure 3 and Figure 5 visualize different approaches to ranking methods, so it is not surprising that their ranking of methods differs.

**Second round of review**

**Reviewer 1**

I don’t wish to get into a terminological disagreement about what it means to call something a “marker gene”. It’s a bit funny that the authors might be required to take the position that it’s something quite different from DE to conclude that their paper is quite different from previous work on DE. But then they do draw the conclusion that simple DE works best. I’m happy to simply put that question of novelty aside and address the manuscript on the merits of its results, which are otherwise quite reasonable. I think three moderate additions are needed:

1) Estimates of the aggregate performance of markers. Individual gene sets vary but does the summated of marker expression add up to something similar (or not) in terms of how it ranks cells? This is not dissimilar to the KNN/SVM-style analysis but more directly assesses the marker like properties.
2) More systematic evaluation of the impact of anything other than DE. This would either firm up the conclusion in favor of the Wilcoxon test or establish other features that are important. I don’t think this needs to be exhaustive across methods, but rather than simply evaluatiing features post-hoc, one could ask what optimal features on top of DE would be present. For example, the use of volcano plots implies fold change is of some independent value to the p-value along
3) I think the divide between real and simulated data is a little too large. Taking the real data and performing more direct perturbations, etc, (or downsamping) to show method use and stability would be helpful.

More broadly, the manuscript could also spend more effort in characterizing patterns in the obtained performance. The overall narrative is plausible but even if there are few patterns in the results (other than what works best), the methods themselves (or their underlying principles) could be more conveniently grouped.

**Authors Response**

**Point-by-point responses to the reviewers’ comments:**

Dear Genome Biology editors,

Thank you for the opportunity to revise and resubmit our paper “A comparison of marker gene selection methods for single-cell RNA sequencing data”.

This revised version of the manuscript includes a number of major changes in response to Reviewer #1’s remaining handful of comments.

First, we have included new estimates of the aggregate performance of marker genes, as requested.

Second, we added more systematic evaluation of other characteristics of marker genes beyond differential expression that highlight similarities and differences between marker gene selection methods.

Third, we have added a set of perturbation analyses using downsampling to bridge the gap between real and simulated datasets noted by the Reviewer.

Finally, we have followed the Reviewer’s request to characterise patterns obtained in method performance, which we hope improves readability and allows the reader to more easily gain insight into the methods and their comparative performance attributes.

Please see below for a full point-by-point response to the Reviewer #1’s comments, and descriptions of changes made to the manuscript main text and supplementary material.

Yours faithfully,

Davis J. McCarthy and Jeffrey Pullin

**Reviewer #1:**

I don’t wish to get into a terminological disagreement about what it means to call something a “marker gene”. It’s a bit funny that the authors might be required to take the position that it’s something quite different from DE to conclude that their paper is quite different from previous work on DE. But then they do draw the conclusion that simple DE works best. I’m happy to simply put that question of novelty aside and address the manuscript on the merits of its results, which are otherwise quite reasonable. I think three moderate additions are needed:

We thank the reviewer for the opportunity to respond to comments on the merits of the results of the paper. We have endeavored to complete the three moderate additions the reviewer has requested.

1) Estimates of the aggregate performance of markers. Individual gene sets vary but does the summated of marker expression add up to something similar (or not) in terms of how it ranks cells? This is not dissimilar to the KNN/SVM-style analysis but more directly assesses the marker like properties.

We thank the reviewer for raising this interesting question. It is certainly relevant to assess how aggregated marker gene expression performs as a classifier for the cell types present in a sample. To address this question, we have expanded the predictive performance (KNN/SVM analysis) section of the manuscript, adding a classifier that directly uses the summated expression of marker genes identified by each marker gene selection method. Precisely, this classifier first calculates the sum of the marker gene expression values for each cell and cluster. Each cell is then assigned to the cluster with the highest summed value for that cell. We assessed the classifier in the same way as the KNN/SVM classifier, calculating the F1 score for each cluster and ranking methods across datasets by the z-score median F1 score. Overall, the new ‘maximum sum’ classifier showed weaker predictive performance than the KNN/SVM classifiers but its ranking of methods when compared across datasets was similar to the other classifiers.

We have included the results for the “maximum sum” classifier in this revision in Supplementary Figure 18 (also shown immediately below for convenience).

We have added the following to the main text to describe these new results for the reader:

“””

Three different classifiers were tested: a KNN classifier, an SVM classifier, and a more direct alternative: classifying each cell as the cluster with the highest summed expression across that cluster's marker genes. (In general, the methods gave highly concordant results so we only visualise the results from the KNN classifier in the main text.) The KNN and SVM classifiers were trained using the marker genes pooled across cell types and assessed using 5-fold cross validation, while the summed max classifier was tested using the 5-fold test sets as it had no tunable parameters. While conceptually distinct from other comparison methods applied in this paper, this comparison was able to reproduce features seen in other comparisons, such as the difficulty of distinguishing between the Naive CD4 T and Memory CD4 T cells in the pbmc3k dataset (\autoref{fig:pred-perf}a). To assess the performance of the methods we calculated a median F1 score across clusters for each fold and dataset (\autoref{fig:pred-perf}b). The classifiers gave similar F1 scores, though the summed maximum expression classifier had generally lower performance (\autoref{fig:pred-perf}b, Additional file 1: Fig S18a, S19a). To summarise each method's performance across datasets we computed the z-score transformation of median F1 score within datasets, noting the low between-fold variability, and then ranked the methods by their mean z-scores (lowest first) across datasets (\autoref{fig:pred-perf}c, Additional file 1: Fig S18b, S19b). Using z-scores accounts for the wide variability in F1 scores observed across datasets, likely due to differences in the quality of their clusterings. The three classifiers gave similar rankings. Across datasets and classifiers the best performing methods are limma (trend), Seurat logistic regression, Wilcoxon-test methods, and t-test methods other than Seurat's t-test; the worst performing methods included Cepo, scran's \texttt{scoreMarkers()} methods, Seurat's t-test method, NSForest, absolute value log fold-change ranking, and scran's binomial test methods.

And added the following to the Methods to describe the process for the analysis:

“””

The maximum sum classifier was implemented from scratch. For this classifier, the expression values of the marker genes for each cluster were centred and scaled and then summed across genes. This process results in a vector of summed expression values across clusters for each cell. The cell is then classified as the cluster with the largest summed expression value. As the maximum summed expression classifier had no tunable parameters it was just assessed on the test sets.

Supplementary Figure 18 (new in this revision)

2) More systematic evaluation of the impact of anything other than DE. This would either firm up the conclusion in favor of the Wilcoxon test or establish other features that are important. I don’t think this needs to be exhaustive across methods, but rather than simply evaluatiing features post-hoc, one could ask what optimal features on top of DE would be present. For example, the use of volcano plots implies fold change is of some independent value to the p-value along

We thank the reviewer for raising the idea of directly comparing methods based on the features of the marker genes they select; we agree that such a comparison is useful and have made additions to the manuscript showing new results accordingly, which appear in an updated Figure 2 in the main text (shown below for convenience).

To perform this comparison we calculated a series of features, one set of which summarizes general features of the marker genes (overall gene mean, overall gene variance, cluster-specific mean, cluster-specific variance and proportion that are up-regulated; new Figure 2b) and one set of features that summarize the effect size of the marker genes (AUC, log fold-change and Cohen’s D statistic; new Figure 2c). We have included plots that visualize these two sets of features for all methods in the bottom panels of Figure 2 in the manuscript. We feel that the general features provide substantial information about methods and the selected genes but do not form a basis for ranking methods. On the other hand, as the reviewer highlights, it is desirable for methods to select genes with a large log fold-change and a large effect size in general, so in Figure 2c we rank the methods based on their average effect size measure.

The general features plot (Figure 2b) illustrates a number of interesting differences between methods. First, there are large differences in the proportion of up-regulated marker genes they select (discussed in the previous version of the manuscript, originally Figure 2a). Second, both between and within software packages there are large differences in the cluster-specific mean-expression for selected marker genes. The COSG and edgeR methods select genes with systematically lower expression, while scran’s scoreMarkers methods generally select marker genes with high expression in the cluster of interest. Within the set of scoreMarkers methods, however, methods that use binary expression values (‘detected’ expression) select marker genes with much lower mean expression. We feel this new figure presents a large amount of information about the characteristics of the marker genes that methods select, allowing readers to pick methods based on the characteristics of the marker genes (i.e., high gene mean, low variance) that they wish to select in their own data.

On the other hand, the effect-size features plot provides a direct comparison of the methods’ ability to select useful high-effect-size genes as marker genes. We measure effect size in three ways: log-fold change, AUC and Cohen’s D, which give distinct but broadly highly concordant rankings of methods. This comparison captures the idea that methods should select genes with the optimal feature of large effect sizes, not just genes that show highly significant DE. Across the ranking of methods, Scanpy’s methods and presto (Wilcoxon rank sum test) select the genes with the highest effect sizes, while scan’s scoreMarkers methods that use binarised expression and scran’s findMarkers any methods select genes with the smallest effect sizes. We also observed that in the marker gene setting AUC and and Cohen’s D statistics were highly concordant but less concordant with the raw log fold-change.

We have added the following to the main text to describe these new results for the reader: “””

Next, we examined the characteristics of the marker genes produced by the analysis. First, we calculated a series of metrics that summarise general properties of the selected marker genes, including the overall mean expression of the gene selected and the proportion of selected marker genes that were up-regulated (\autoref{fig:concordance-figure}b). In this context, up-regulated means that the gene is more highly expressed in the cluster of interest than in other clusters. These metrics were calculated for the at most top 5 genes selected by each method in the all real datasets and the median over clusters and datasets was visualised. These metrics highlight a number of patterns amongst the marker genes that the different methods select. Most methods select a majority of up-regulated marker genes. Indeed, some methods including including presto, scran's \texttt{findMarkers()} Wilcoxon and \texttt{scoreMarkers()} methods, and the Scanpy's raw methods select almost all up-regulated genes, while SMaSH and methods based on negative binomial GLMs, Welch's t-test and binomial tests select more down-regulated marker genes. In addition, the genes that methods select differ in terms of the mean and variance of their cluster-specific expression. For example, scran's \texttt{scoreMarkers()} methods that binarised expression ('detected' expression) select genes with systematically lower expression. On the other hand, Seurat's methods show systematically higher variance in the cluster of interest compared to scran's \texttt{scoreMarkers()} methods.

Second, we calculated measures of effect sizes for the selected marker genes. Ideally, methods would select genes which have a large difference in gene expression in the cluster of interest, compared to expression in all other clusters. To measure the effect size we used: AUC (i.e., area under the receiver operator characteristic curve using the expression data as a classifier for binary cluster status) and absolute values of Cohen's d statistic and the log fold-change, all in a one-vs-rest manner. These metrics were calculated for the at most top 5 genes selected by all methods. For visualisation the median was taken over clusters and datasets, and quantile normalisation was applied to make the metrics' distributions comparable. Methods are ranked by their median values over the three metrics (\autoref{fig:concordance-figure}c). Of the three metrics, Cohen's d and AUC are highly concordant. Presto and Scanpy's methods select genes with the largest overall effect sizes, while scran's \texttt{findMarkers()} `any' methods, Scanpy's t-test (abs), and ranking by the absolute value of the log-fold change have the lowest effect median effect sizes. The genes selected by ranking by the absolute value of the log fold-change generally have extremely low expression in the cluster of interest and low expression in all other clusters causing large log fold-changes but small differences in other effect sizes.

And added the following to the Methods to describe the process for the analysis:

“””

To assess the methods generally we calculated the following metrics:

\begin{itemize}

\item Cluster mean: the mean of log-normalised expression values in the cluster of interest

\item Cluster variance: the variance of log-normalised expression values in the cluster of

interest

\item Gene mean: the mean of all log-normalised expression values

\item Gene variance: the variance of all log-normalised expression values

\item Proportion up-regulated the proportion of selected genes that were up-regulated (i.e.

had a log fold-change greater than 0)

\end{itemize}

These metrics were calculated for the at most top 5 genes selected by each method in the all real datasets. To visualise the results the median over clusters and datasets was taken.

To assess the effect size of the selected genes we used the following metrics:

\begin{itemize}

\item AUC (i.e., area under the receiver operator characteristic curve using the expression

data as a classifier for binary cluster status)

\item the absolute value of Cohen's d statistic

\item the absolute value of log fold-change

\end{itemize}

All statistics were calculated in a one-vs-rest manner for the at most top 5 genes selected by each method in the all real datasets. To visualise the results the median over clusters and datasets was taken and quantile normalisation was performed to make the distributions of the three metrics comparable. In the figure, methods were ranked by the median of the quantile normalised values of the three metrics.

Figure 2 (panels b and c updated/replaced in this revision)

3) I think the divide between real and simulated data is a little too large. Taking the real data and performing more direct perturbations, etc, (or downsamping) to show method use and stability would be helpful.

We thank the reviewer for this insightful suggestion for bridging the gap between analyses based on simulated data and those based on real data. As noted previously, capturing all salient

7

features of real data with simulated data is deeply challenging, while limited “ground truth” in real datasets constrains the types of comparisons that can be made with real data. As such, we appreciate the suggestion to directly perturb real data with downsampling to delve further into method use and stability.

Following this suggestion, we have added additional perturbation experiments which use downsampling to assess method stability when removing clusters (or cell types) and cells from a real dataset. Briefly, the exemplar pbmc3k dataset is downsampled to decrease either the number of cells or the number of clusters (Figure S28; also shown below for convenience). Method stability is assessed by calculating the proportion of at most top 10 genes selected that are the same as the genes selected when the methods are run on the full dataset. These perturbations show that as both the number of cells and clusters decrease method performance decreases, but the decrease is larger when the number of clusters decreases, highlighting methods' general sensitivity to the cluster structure. Methods that had generally better performance in the benchmarking were more stable when the number of cells was reduced, but scran's \texttt{scoreMarkers()} methods showed good stability when the number of clusters was reduced, likely due to its use of a pairwise comparison approach.

Supplementary Figure 28 (new in this revision)

We have added the following to the main text to describe these new results for the reader:

“””

Next, to assess the effect of dataset characteristics on method stability we performed simulations where the number of clusters and cells were reduced by down-sampling the pbmc3k dataset (Additional file 1: S28). Method stability is assessed by calculating the proportion of at most top 10 genes selected that are the same as the genes selected when the methods are run on the full dataset. These simulations demonstrate that as both the numbers of cells and clusters were reduced method performance decreased compared to performance on the full dataset. These perturbations show that as both the number of cells and clusters decrease method performance decreases, but the decrease is larger when the number of clusters decreases, highlighting methods' general sensitivity to the cluster structure. Methods that had generally better performance in the benchmarking were more stable when the number of cells was reduced, but scran's \texttt{scoreMarkers()} methods showed good stability when the number of clusters was reduced, likely due to its use of a pairwise comparison approach.

And added the following to the Methods to describe the process for the analysis:

“””

\subsection*{Stability simulations}

To assess the stability of methods we performed simulations by down-sampling either the number of clusters or the number of cells in the exemplar pbmc3k dataset. Cells were down-sampled by randomly removing 25\%, 50\% and 75\% of all cells. Clusters were removed one-by-one until only two clusters remained. The ordering of clusters for removal was: Naive CD4 T, CD14+ Mono, Memory CD4 T, Platelet, CD8 T, FCGR3A+ Mono, NK, DC, B. All methods were run and the at most top 10 marker genes were retained. Method stability was assessed by calculating the proportion of genes that were the same as in the full pbmc3k dataset, taking the median over the clusters.

More broadly, the manuscript could also spend more effort in characterizing patterns in the obtained performance. The overall narrative is plausible but even if there are few patterns in the results (other than what works best), the methods themselves (or their underlying principles) could be more conveniently grouped.

We thank the reviewer for raising this suggestion and agree that grouping methods in benchmarking paper is important for readability. Indeed, we have sought to do this in several places in the manuscript. First, the supplementary material includes a comprehensive table of methods that readers can consult for further grouping, classification, and insight into the various methods benchmarked in this work. We have now added a reference to this table in the main text, at the start of the section Method Characteristics, which was accidentally omitted in the last submitted version. Second, in the section “Method Characteristics” we give an overview of methods and describe several groups methods fall into. Third, Figure 2a provides a visual representation of differences in methods in two ways, whether they return a fixed set or a ranking of genes, and whether they select up-regulated genes only. Finally, the Methods section gives a comprehensive overview of principles of the methods and important parameters.

**Reviewer #1:**
Looks great to me. No additional comments.
